# Artificial Intelligence in Patient Blood Management: A Systematic Review of Predictive, Diagnostic, and Decision Support Applications

**DOI:** 10.3390/jcm14238479

**Published:** 2025-11-29

**Authors:** Henrique Coelho, Fernando Silva, Marta Correia, Pedro Miguel Rodrigues

**Affiliations:** 1 CBQF—Centro de Biotecnologia e Química Fina—Laboratório Associado, Escola Superior de Biotecnologia, Universidade Católica Portuguesa, Rua de Diogo Botelho 1327, 4169-005 Porto, Portugal; mmcorreia@ucp.pt (M.C.); pmrodrigues@ucp.pt (P.M.R.); 2 Serviço de Hematologia, Unidade Local de Saúde de Vila Nova Gaia e Espinho, Rua Conceição Fernandes S/N, 4434-502 Vila Nova de Gaia, Portugal; 3 Departamento de Ciências Médicas, Campus Universitário de Santiago, Universidade de Aveiro, Agra do Castro, Edifício 30, 3810-193 Aveiro, Portugal; 70836@ulsra.min-saude.pt; 4 Serviço de Hematologia, Unidade Local de Saúde da Região de Aveiro, Avenida Artur Ravara, 3814-501 Aveiro, Portugal

**Keywords:** artificial intelligence, machine learning, deep learning, patient blood management, transfusion medicine, clinical decision support

## Abstract

**Background**: Patient blood management (PBM) is a patient-centered, evidence-based approach for optimizing anemia management, minimizing blood loss, and ensuring appropriate transfusion. Artificial intelligence (AI) provides powerful tools for prediction, diagnosis, and decision support across PBM, but current evidence remains emerging and not yet consolidated. **Objectives**: This review synthesizes AI applications in PBM, summarizing predictive, diagnostic, and decision support models; highlighting methodological trends; and discussing challenges for clinical translation. **Methods**: PubMed, Scopus, and Web of Science were searched from inception to 31 March 2025. Eligible studies reported AI models addressing the three established PBM pillars. Studies on transfusion safety and blood bank operations relevant to PBM were also included. Extracted data covered study characteristics, predictors, models, validation strategies, and performance. The findings were narratively synthesized given study heterogeneity. **Results**: A total of 338 studies were included, spanning anemia detection, bleeding risk stratification, transfusion prediction, transfusion safety, and inventory management. Deep learning (DL) predominated in image-based anemia detection, while ensemble and gradient boosting methods frequently outperformed baselines in bleeding and transfusion risk prediction. Recurrent and hybrid architectures proved effective for blood supply forecasting. Across domains, machine learning and DL models generally surpassed logistic regression, clinical scores, and expert judgment. Despite strong internal performance, external validation and clinical deployment remain limited. **Conclusions**: AI is advancing PBM by enabling earlier anemia detection, more accurate bleeding and transfusion prediction, and smarter resource allocation. Translation into practice requires standardized reporting, robust external validation, explainability, and workflow integration. Future work should emphasize multimodal learning, prospective evaluation, and cost-effectiveness.

## 1. Introduction

Patient blood management (PBM) has emerged as a global standard of care recognized by the World Health Organization (WHO) for optimizing transfusion practice [1]. By reducing unnecessary transfusions and correcting anemia or bleeding disorders before interventions, PBM lowers complication risks, accelerates recovery, and decreases healthcare costs [1]. At the system level, PBM also safeguards blood supply adequacy by promoting efficient use and minimizing wastage [1].

In parallel, artificial intelligence (AI) has rapidly advanced across healthcare, providing data-driven solutions for prediction, decision support, and resource management [2]. Within PBM, AI holds promise. Algorithms can integrate diverse patient data—including demographics, laboratory results, and imaging—to predict anemia and transfusion requirements, stratify bleeding risk, and personalize transfusion thresholds [2]. At the operational level, AI-based tools can forecast blood product demand, optimize inventory allocation, and streamline logistics, thereby reducing costs and enhancing supply resilience [2].

Integrating AI into PBM offers dual benefits: improved clinical outcomes and more efficient use of healthcare resources [2]. Nevertheless, despite a growing body of research, the field remains heterogeneous, and the translation of AI tools into routine practice is still limited [2]. A scoping review by Meier and Tschoellitsch synthetized available studies on AI in PBM up to October 2021, providing an important first overview of this emerging field [2]. Since then, the literature has expanded substantially, with new studies employing advanced machine learning (ML)/deep learning (DL) architectures and multimodal datasets [3].

The objective of this review is to map and critically appraise the current landscape of AI applications in PBM, building on previous scoping evidence and incorporating recent advances. We aim to highlight methodological trends, identify persisting gaps, and discuss the translational potential of AI-driven PBM strategies for clinical practice.

### 1.1. Current Landscape in PBM

PBM represents a comprehensive, evidence-based strategy tailored to optimize patient outcomes while minimizing unnecessary blood transfusions and their associated risks [4]. Key guidelines from professional organizations—including the American Association of Blood Banks (AABB) and the Society for the Advancement of Blood Management (SABM)—offer recommendations on transfusion thresholds, clinical indications, and alternatives to transfusion, thereby promoting consistency in clinical practice [5,6]. At a global policy level, the WHO published its *Guidance on Implementing Patient Blood Management to Improve Global Blood Health Status* in 2025 [7]. This landmark document urges healthcare systems to adopt PBM as an essential element of safe and sustainable care, particularly in resource-limited settings. It outlines a phase-wise implementation strategy, including system-level preparation, pilot program rollout, and national scale-up, and provides contextualized toolkits adapted to varying resource levels. The WHO emphasizes that PBM has the potential to enhance blood health, reduce anemia-related morbidity, and narrow global health inequities [7].

Despite these advances, several challenges persist. Variability in transfusion practice across institutions leads to inconsistent decision-making and, at times, suboptimal outcomes [8]. Determining appropriate transfusion thresholds can be complex and requires careful consideration of individual patient factors, underlying comorbidities, and contextual clinical circumstances [9]. Achieving the balance between avoiding unnecessary transfusions and ensuring adequate oxygen delivery remains a key clinical dilemma. Moreover, blood products are finite and costly resources. Securing a reliable supply, particularly under emergency or resource-constrained scenarios, is both essential and challenging. The COVID-19 pandemic, for instance, starkly illuminated the potential fragility of blood systems amid supply disruptions [10]. These multifaceted challenges underscore the need for ongoing research, structured implementation, and coordinated collaboration among healthcare stakeholders. PBM’s multidisciplinary and patient-centered approach could enhance the quality, safety, and efficiency of blood management practices, ultimately improving patient outcomes, reducing costs, and conserving essential resources. In this context, AI offers new opportunities to address persistent challenges in PBM by reducing practice variability, refining transfusion thresholds, enhancing decision support, and optimizing blood resource utilization, thereby complementing and strengthening evidence-based transfusion practices [11,12].

### 1.2. AI Models Relevant to PBM

AI applications in PBM have evolved rapidly, supporting anemia detection, bleeding risk stratification, transfusion prediction, transfusion safety, and blood supply optimization [12]. Across the literature, models can be grouped into five broad categories:

(i) Traditional machine learning models: Logistic regression (LR), decision trees (DT), support vector machines (SVM), naïve Bayes (NB), and k-nearest neighbors (KNN) remain widely applied to structured data. These are often benchmarked against clinical scores and are valued for their interpretability in practice [13].
(ii) Ensemble methods: Random forests (RF), gradient boosting (GB), AdaBoost, and voting classifiers (VC) frequently outperform single algorithms, particularly when applied to heterogeneous or imbalanced datasets [13].
(iii) DL architectures: Deep neural networks (DNN), convolutional neural networks (CNN), and recurrent models such as long short-term memory (LSTM) and gated recurrent units (GRUs) are well suited to high-dimensional and temporal data, including laboratory time series, imaging, and electronic health records (EHRs). Transformer-based models (e.g., BERT, ViT) and attention mechanisms are emerging for tasks such as radiomics, sequential pattern recognition, and analysis of clinical narratives [14].
(iv) Probabilistic and interpretable models: Bayesian networks, Gaussian processes, and physics-informed neural networks enable uncertainty estimation and offer greater transparency, critical in clinical decision-making contexts such as transfusion thresholds or rare blood group management [13].
(v) Hybrid and meta-learning approaches: Variational autoencoders, stacked generalization, and neuro-fuzzy systems facilitate multimodal integration of laboratory, imaging, and clinical data, aligning well with the multidimensional demands of PBM. In this review, hybrid models are defined as approaches that combine two or more distinct algorithms (e.g., ML with DL, or ensembles of heterogeneous methods) to leverage complementary strengths and improve overall performance [14].

Across categories, AI models consistently outperform traditional regression and established clinical scoring systems. Their ability to integrate diverse data sources and adapt across clinical domains highlights their translational potential for real-world PBM strategies [12].

### 1.3. Aims and Objectives

This review provides a comprehensive synthesis of AI applications in PBM, focusing on models that perform the following functions:

(i) Predict anemia, transfusion requirements, or bleeding risk;
(ii) Support transfusion decision-making;
(iii) Enhance transfusion safety;
(iv) Optimize blood bank operations.

For each included study, we summarized the PBM domain, sample size and case group characteristics, model inputs (variables and predictors), model types and implementation details, validation approach, and reported performance. This structured synthesis highlights methodological trends, clinical applications, and the translational potential of AI-driven strategies in PBM.

## 2. Materials and Methods

### 2.1. Eligibility Criteria

We included original studies that reported the development, validation, or application of AI models within PBM. Eligible domains encompassed the following: (i) prediction of anemia, transfusion requirements, or bleeding risk; (ii) transfusion decision support; (iii) transfusion safety; (iv) blood bank operations and management. Studies were excluded if they did not involve AI methods, were unrelated to PBM, focused solely on laboratory techniques without clinical application, were conducted on animals or in vitro, or were reviews, editorials, commentaries, or conference abstracts. Publications were restricted to English, Portuguese, and Spanish.

This review was not registered in PROSPERO because the protocol was finalized and data extraction completed prior to submission. However, it was conducted in accordance with PRISMA 2020 guidelines [15]. The full PRISMA checklist is provided in the Supplementary Materials.

### 2.2. Search Strategy

**Figure 1:**
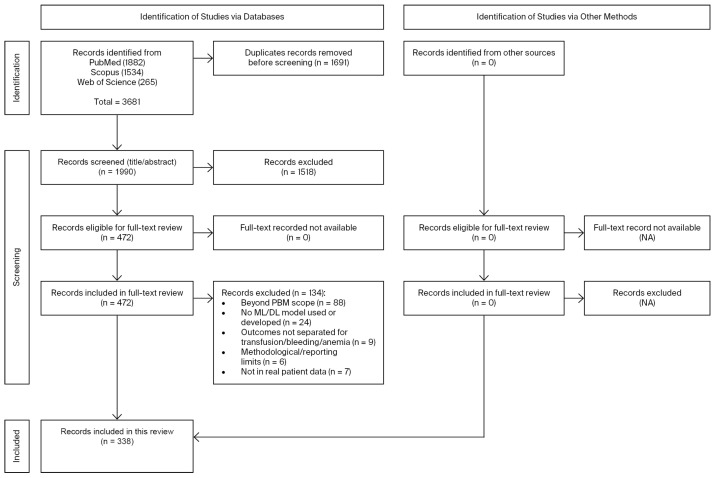
PRISMA flow diagram of study selection. Of 3681 records identified, 1990 were screened after duplicates were removed, and 338 studies met the inclusion criteria.

PubMed, Scopus, and Web of Science were searched from inception to 31 March 2025. The PubMed query was (“machine learning” [All Fields] OR “artificial intelligence” [All Fields]) AND (“anemia” [All Fields] OR “bleeding” [All Fields] OR “transfusion” [All Fields] OR “patient blood management” [All Fields]), retrieving 1882 records. Equivalent searches yielded 1534 in Scopus and 265 in Web of Science. In Scopus, the search was limited to articles. After removing duplicates (n = 1691), 1990 records remained. Title/abstract screening excluded 1514, leaving 472 for full-text assessment. Of these, 134 were excluded (beyond PBM scope: 88; no ML/DL model used or developed: 24; outcomes not separated for transfusion/bleeding/anemia: 9; methodological/reporting limitations: 6; no real patient data: 7). The study selection process is summarized in Figure 1. In total, 338 studies were included. No additional records were identified through other sources, and all full texts were available. The complete list of included studies, with full references, is provided in the Supplementary Materials, while Tables S1–S4 summarize the distribution of studies across anemia, bleeding, transfusion, and other PBM domains.

### 2.3. Study Selection

Records retrieved from the databases were imported into Zotero (v7.0.24, Corporation for Digital Scholarship, Fairfax, VA, USA), and duplicates were removed. Two reviewers independently screened titles and abstracts; full texts of potentially eligible articles were assessed against the predefined criteria. Disagreements were resolved through consensus or, when needed, by a third reviewer. The study selection process is illustrated in a PRISMA flow diagram (Figure 1).

### 2.4. Data Collection Process

Two reviewers independently extracted data using a piloted form. Extracted items included reference (first author, year), PBM domain, sample size and case group characteristics, model inputs and top predictors, AI model(s) used, validation strategy, and reported performance metrics. Discrepancies were resolved through consensus or by a third reviewer.

### 2.5. Risk of Bias

No single risk-of-bias tool was applied due to the heterogeneity of the models, outcomes, and validation strategies. Instead, two reviewers qualitatively assessed common risks in prediction modeling: small sample size, class imbalance, data leakage, handling of missing data, validation rigor, and adequacy of performance evaluation. Disagreements were resolved through consensus or by a third reviewer.

### 2.6. Synthesis

As part of this systematic review, we conducted a structured narrative synthesis due to substantial clinical and methodological heterogeneity across studies. Included studies were grouped by PBM domain: anemia detection or prediction, bleeding risk, transfusion prediction and decision support, transfusion safety, and blood bank operations. Within each domain, we summarized the study setting, data modality, model type, validation approach (internal, external, or temporal), and performance. When multiple models were tested on the same dataset, the most rigorously validated or best-performing model was prioritized to avoid redundancy. Calibration findings and explainability techniques were reported when available. Statistical summaries were performed using IBM SPSS Statistics, version 30.0 (IBM Corp., Armonk, NY, USA).

All 338 included studies are listed with full citation details in Supplementary Tables S1–S4. To maintain clarity and conciseness in the main text, only representative or flagship studies discussed in Section 3 are included in the reference list. A complete list of studies excluded at the full-text screening stage, along with reasons for exclusion, is provided in Supplementary Table S5.

## 3. Results: AI Applications in PBM

AI has been applied across the full spectrum of PBM, targeting both clinical decision-making and operational optimization. The studies reviewed here illustrate applications aligned with the three classical PBM pillars—optimizing red cell mass, minimizing blood loss and bleeding, and harnessing tolerance of anemia—as well as transfusion safety and blood bank operations. Evidence is organized into five domains: (i) anemia detection and prediction, where AI supports early diagnosis and proactive management; (ii) bleeding prediction, encompassing high-risk settings such as cardiology, gastroenterology, and critical care; (iii) prediction of transfusion requirements, including perioperative and ICU contexts; (iv) decision support and transfusion safety, where AI augments or surpasses clinical scores and enhances laboratory compatibility testing; and (v) blood bank operations and resource optimization, where AI improves demand forecasting, inventory management, and waste reduction. This structure reflects both the clinical and operational aspects of PBM, underscoring AI’s potential to improve patient outcomes while promoting sustainability in blood product use.

### 3.1. Anemia Detection and Prediction

AI has been increasingly applied to anemia detection and prediction using imaging, physiological signals, laboratory data, and multimodal approaches. DL applied to ocular and facial images achieved strong non-invasive performance. In a dataset of >5800 retinal images, InceptionV3 achieved 98% accuracy and an AUC (area under the curve) of 0.98 for anemia detection, with a mean absolute error (MAE) of 0.58 g/dL for hemoglobin (Hb) estimation, validated in an external cohort [16]. Stacked ensemble models trained on palmar or conjunctival images reported near-perfect discrimination, with AUCs ranging from 0.97 to 1.0 and accuracies close to 100% [17,18]. Similarly, CNNs applied to palmar, fingernail, and conjunctival images consistently achieved excellent results, with reported accuracies above 97% and AUCs up to 0.999 [19].

Signal- and physiology-based approaches have expanded the scope of non-invasive anemia assessment. CNNs trained on electrocardiogram features from nearly 40,000 patients achieved AUCs of 0.92 internally and 0.90 externally, outperforming LR [20]. Nailbed optical spectra analyzed with ANN correlated strongly with laboratory Hb (r = 0.987) [21]. Fingertip video-based CNNs and fusion models also showed promise: the best Fusion-1 model achieved an RMSE (root mean square error) of 0.63 and an MSE (mean square error) of 0.61, with sensitivity of 0.97 and specificity of 0.68 [22]. A multimodal CNN fusion model integrating fingertip video signals achieved accuracy of 98.4% and an AUC of 0.988, demonstrating excellent discriminatory ability in anemia detection [23].

Multimodal models have demonstrated added value for early prediction; in the UK Biobank, combining fundus images with demographic metadata improved discrimination compared with metadata alone (AUC 0.88 vs. 0.74) [24]. Recurrent neural networks (RNN) trained on laboratory time-series predicted iron deficiency anemia (IDA) up to six months before conventional diagnosis, with GRUs achieving an AUC of 0.89 [25]. In dialysis populations, GRU and LSTM models forecasted Hb trajectories with MAEs of 0.5–0.7 g/dL, values comparable to routine laboratory variability, supporting their potential role in proactive erythropoiesis-stimulating agent titration [26,27].

AI has also been applied to hematologic conditions with direct implications for PBM. Differentiating IDA from thalassemia is clinically critical, as the two require distinct management strategies. SVM and XGB (XGBoost, Extreme Gradient Boosting) models achieved AUCs of 0.88–0.96 and consistently outperformed classical discriminant formulas [28,29,30]. RF and CNN models achieved AUCs around 0.95, with one study reporting a 60% reduction in unnecessary genetic testing [31,32]. For sickle cell disease, CNN and SVM classifiers achieved accuracies ranging from ~90% to >95% using blood smears, digital scans, and microfluidic imaging [33,34,35].

### 3.2. Bleeding Prediction

In cardiology, AI models trained on national PCI (percutaneous coronary intervention) registries have consistently outperformed established clinical scores. In a dataset of more than 3.3 million PCI procedures, XGB improved bleeding prediction compared with the NCDR risk model (C-statistic 0.82 vs. 0.78) [36]. Validation in over 100,000 PCI cases confirmed superior performance, with AUCs of 0.887 for bleeding and 0.917 for transfusion, both exceeding LR [37]. Another large-scale study demonstrated that an AI-based bleeding risk model achieved an AUC of 0.873, outperforming the ACC-BR score [38]. Collectively, these findings highlight AI’s ability to enhance peri-procedural bleeding risk stratification in interventional cardiology.

In gastroenterology, AI methods have consistently outperformed guideline-based scores and expert assessment. Gradient boosting models predicted variceal bleeding in cirrhosis with AUCs of 0.94 in internal and 0.86 in external validation, surpassing endoscopic classification [39]. A multimodal stacking ensemble achieved an external AUC of 0.975, outperforming MELD and Child–Pugh scores [40]. DL applied to endoscopic images alone reached an external accuracy of 0.893, exceeding the performance of experienced endoscopists [41].

In critical care and surgery, DL models have been evaluated for bleeding surveillance and perioperative risk assessment. GRU-based RNN predicted ICU bleeding risk in patients on antithrombotic therapy more accurately and more rapidly than clinicians (AUC 0.83) [42]. In cardiothoracic surgery, analysis of more than 660,000 cases showed that NN achieved an AUC of 0.97 for postoperative bleeding [43]. Additional ML studies in ICU and surgical populations have further confirmed robust predictive performance in high-risk perioperative contexts [44].

Across multiple surgical specialties, ensemble methods have further demonstrated strong generalizability. In a dataset of 48,543 perioperative cases, Light Gradient Boosting Machine (LightGBM) models achieved an AUC of 0.933 for intraoperative bleeding, validated externally [45]. These findings underscore the ability of AI to generalize across heterogeneous surgical populations, supporting hospital-wide PBM strategies for perioperative bleeding risk prediction.

### 3.3. Prediction of Transfusion Requirements

In surgical populations, ensemble methods such as GB and LGBM consistently outperformed LR and established transfusion scores, achieving strong discrimination in very large datasets. In a study of over four million surgeries, GB reduced unnecessary type-and-screen orders by one-third, translating into measurable cost savings, while LGBM achieved an AUC of 0.93 in more than 100,000 procedures with external validation [46,47].

In intraoperative settings, DL has enabled real-time prediction of massive transfusion. Recurrent architectures such as GRUs anticipated transfusion needs up to ten minutes in advance, maintaining AUCs above 0.94 across both internal and external validation cohorts. These models demonstrate the feasibility of integrating streaming physiological and laboratory data into perioperative decision support [48,49].

In critical care, ML ensembles have been used to support transfusion decisions for patients with gastrointestinal bleeding. By combining LR with RF, these models achieved high sensitivity and strong performance across external validation cohorts of more than 4000 patients, reinforcing their potential for deployment in acute care settings [50].

### 3.4. Decision Support and Transfusion Safety

AI-based decision support systems have demonstrated superiority to traditional risk scores in transfusion-related decision-making. In upper gastrointestinal bleeding, XGB models achieved AUCs of around 0.90 and significantly outperformed established clinical tools such as Rockall, AIMS65, and the Glasgow Blatchford Score, with consistent performance after external validation. These findings suggest that AI can provide more accurate triage and intervention support than guideline-based scoring systems [51].

Causal inference methods using ML have also been applied to evaluate transfusion strategies and donor characteristics. Targeted maximum likelihood estimation and SuperLearner ensembles provided insights into differential risks associated with restrictive versus liberal transfusion strategies in acute myocardial infarction, the impact of donor sex on recipient outcomes, and the role of plasma-to-RBC ratios in trauma resuscitation. These applications highlight AI’s potential not only for prediction but also for estimating causal effects, thereby informing transfusion policy and clinical practice [52,53,54].

In transfusion safety, AI has been applied to genomic, phenotypic, and imaging data. RF and XGB models predicted blood group antigens in over 110,000 donors with near-perfect accuracy, substantially improving precision in donor–recipient matching. DL approaches enhanced RH genotyping concordance, while CNN ensembles automated Coombs test interpretation with >99% accuracy, surpassing expert immunologists. These advances demonstrate the feasibility of embedding AI into laboratory workflows to strengthen compatibility testing and reduce adverse events [55,56,57].

### 3.5. Blood Bank Operations and Resource Optimization

AI models have been increasingly applied to blood bank operations, where efficient demand forecasting and inventory management are central to PBM. GB and ensemble methods trained on large surgical datasets improved transfusion risk prediction and reduced unnecessary testing. In a study of more than four million procedures, gradient boosting reduced type-and-screen orders by one-third, generating substantial cost savings [46]. Similarly, in over 100,000 surgeries, LGBM achieved an AUC of 0.93 with external validation, supporting the generalizability of such models [47].

Time-series approaches have also shown strong performance in blood inventory management. A hybrid model combining seasonal trend decomposition with XGB reduced red cell inventory by 38%, eliminated urgent orders, and lowered costs by 43% [58]. For platelet demand, LSTM models tailored to hematology/oncology patients achieved high accuracy (AUC 0.98), supporting early identification of shortages in vulnerable populations [59]. Ensemble learning has also proven effective for daily RBC demand forecasting, improving alignment with actual usage and reducing supply chain risk [60].

AI has also been applied to identify inefficiencies and minimize wastage in blood product supply chains. Association rule mining and RNN uncovered drivers of product disposal and returns, offering actionable insights for optimizing blood product utilization. These studies illustrate AI’s role not only in predictive modeling but also in uncovering hidden patterns that inform stewardship and sustainability in blood banking [61,62].

### 3.6. Comparative Distribution of Model Performance

**Figure 2:**
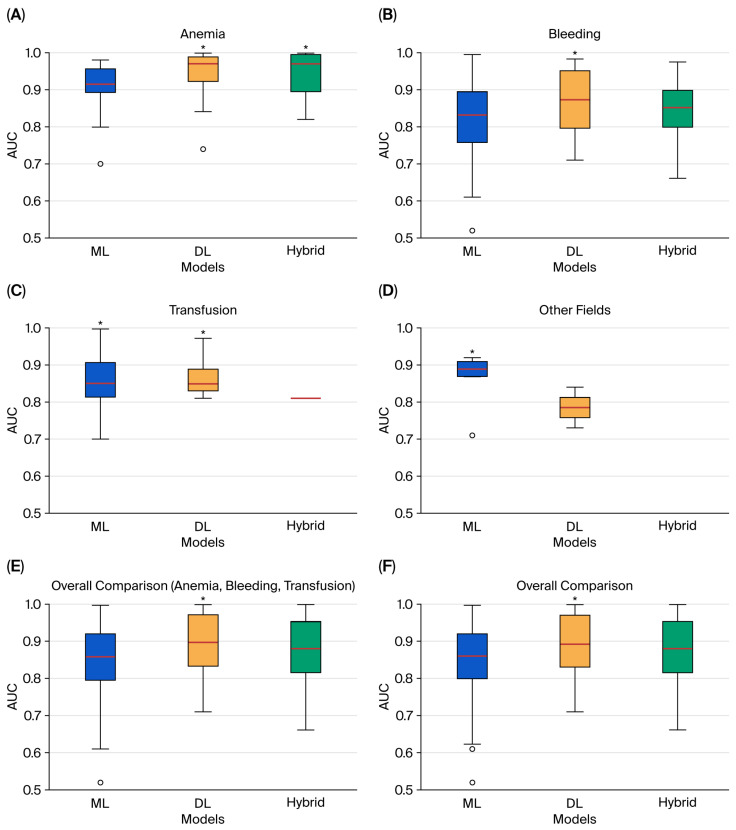
Comparative boxplots of best-performing AUC values for ML, DL, and hybrid models across domains: (**A**) anemia; (**B**) bleeding; (**C**) transfusion; (**D**) other fields; (**E**) overall comparison of anemia, bleeding, and transfusion; (**F**) overall comparison of all domains. Boxes represent the interquartile range, the line inside the box shows the median, and circles denote outliers. An asterisk (*) marks the model category with the highest median AUC.

The distribution of reported model performance is summarized in Figure 2, using boxplots of AUC values stratified by model category (ML, DL, and hybrid). To avoid redundancy, only the best-performing model from each study was extracted. Thus, the plots reflect the upper performance bound for each domain rather than the full range of algorithms tested.

In anemia detection and treatment, both ML (n = 50) and DL (n = 48) models were frequently applied, while hybrid approaches were less common (n = 16). DL and hybrid models consistently achieved higher AUCs, clustering near or above 0.95 with narrow interquartile ranges, while ML model showed greater dispersion. These findings align with the strong results of image-based DL architectures and ensemble methods highlighted in Supplementary Table S1.

In bleeding prediction, ML approaches were most common (n = 81), compared with DL (n = 20) and hybrid (n = 16). The distributions were more heterogeneous: ML performance displayed a wider spread and included several lower outliers (AUC < 0.80), while DL and hybrid models demonstrated higher medians and reduced variability, consistent with their role in perioperative and interventional risk prediction (Supplementary Table S2).

In transfusion prediction, ML again predominated (n = 62), compared with DL (n = 18) and hybrid (n = 2). DL models dominated among the top performers, while only a single hybrid model reached best-in-class status. ML models exhibited greater variability, suggesting performance was highly dependent on dataset quality and feature selection (Supplementary Table S3).

In the other fields category, which included transfusion safety and blood bank operations, the total number of models was limited (ML: 13; DL: 9; hybrid: 5). Here, no hybrid model was identified as the best performer. Both ML and DL achieved competitive AUCs, though variability was greater than in the anemia domain (Supplementary Table S4).

**Figure 3:**
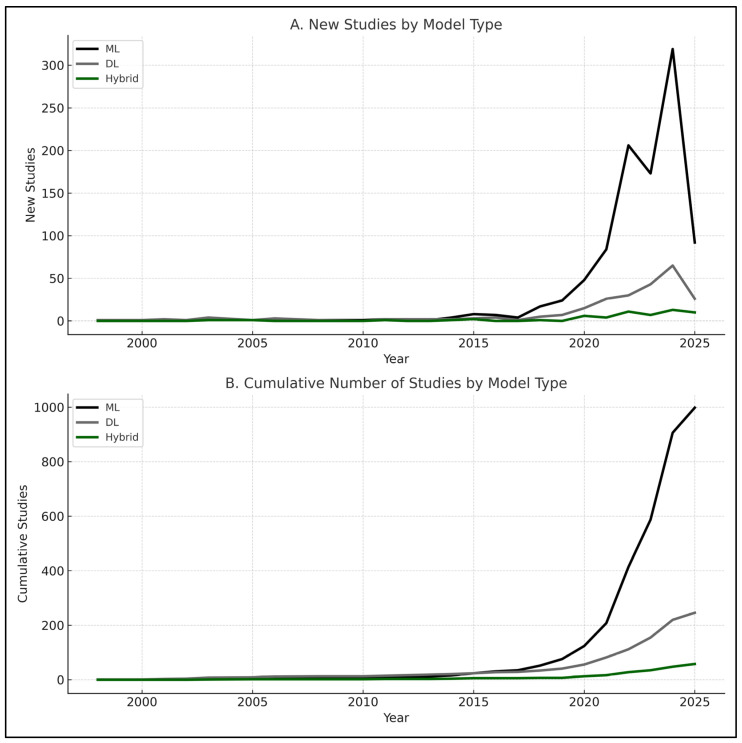
Trends in studies applying ML, DL, and hybrid models over time: (**A**) new studies per year by model type (1998–2025); (**B**) cumulative number of studies by model type (1998–2025). ML dominates in absolute numbers, while DL and hybrid show accelerated growth in recent years. *Note: 2025 data include publications up to March only*.

When aggregating across domains, the overall comparison plots confirmed these trends (Figure 2E,F). DL and hybrid models generally displayed higher central tendencies, while ML results covered a broader range, reflecting both their greater representation in the literature and their sensitivity to dataset heterogeneity. These findings emphasize that, across PBM domains, DL and hybrid approaches more often reached the highest performance levels, particularly in image-rich and multimodal tasks. At the same time, the imbalance in model counts—with ML overwhelmingly more common in bleeding and transfusion—helps explain the wider variability observed for ML in the boxplots. Trends in the publication of ML, DL, and hybrid PBM studies over time are shown in Figure 3.

Overall, AI applications in PBM have demonstrated robust potential across clinical and operational domains. From early anemia detection and bleeding risk stratification to real-time transfusion forecasting, decision support, and blood bank optimization, AI models frequently outperformed LR, clinical scores, and even expert judgment in selected settings. Large-scale studies with external validation highlight the translational promise of these approaches, while real-time and multimodal models illustrate the feasibility of integration into perioperative and critical care workflows. At the same time, evidence remains heterogeneous, with variable study quality, limited calibration reporting, and a paucity of prospective validations. These findings underscore the need for critical appraisal of current evidence, careful consideration of limitations, and identification of future directions, which are addressed in the following section.

## 4. Discussion and Future Directions

This review highlights both the progress and the limitations of AI applications in PBM. Most AI models included in this review were trained and validated exclusively on retrospective datasets. While such datasets allow scalable model development, retrospective designs introduce risk of bias, unmeasured confounding, and potential data leakage. As a result, high retrospective performance cannot be assumed to translate into real-world clinical benefit. To determine true utility and safety, future work must incorporate prospective, real-world evaluations—such as silent-mode deployment within EHR or pragmatic clinical trials—to assess how AI models behave in everyday PBM workflows. A further challenge relates to generalizability. Several high-performing AI models were developed using national or multinational datasets, yet such models may not perform equivalently when applied to smaller or demographically distinct institutions. Differences in laboratory methods, transfusion practices, case mix, and disease prevalence can significantly reduce model accuracy when transferred to local contexts. Therefore, external validation alone is insufficient; local calibration, transfer learning, or domain adaptation may be required before these models can be safely implemented in smaller centers. It is also important to emphasize that AI should not be regarded as a universal replacement for traditional statistical modeling. In settings characterized by limited sample size, low event prevalence, or restricted access to robust data infrastructures, classical approaches—such as logistic regression or guideline-based clinical scores—may remain more reliable, interpretable, and clinically appropriate. In such environments, AI may serve as a complementary tool rather than a primary decision support modality. Across domains, AI models frequently outperformed LR and established clinical scores when direct comparisons were performed. In interventional cardiology, XGB improved bleeding risk stratification in more than 3.3 million PCI procedures and outperformed the NCDR model [36], while Hamilton et al. [37] confirmed these findings in over 100,000 PCI cases with external validation. In upper gastrointestinal bleeding, XGB surpassed Rockall, AIMS65, and the Glasgow Blatchford Score [51]. In internal medicine, ML achieved superior discrimination compared with RIETE and VTE-BLEED scores in anticoagulated patients [63,64]. Large-scale studies with external validation further demonstrated generalizability, including transfusion prediction across more than 100,000 surgeries [47], perioperative bleeding in 48,543 procedures [45], and postoperative bleeding in over 660,000 cardiothoracic cases [43].

Several studies extended beyond prediction to address causal inference and transfusion safety. Portela et al. [54] and Nguyen et al. [53] applied targeted learning to evaluate restrictive versus liberal transfusion strategies and plasma-to-RBC ratios in trauma, while Hyvärinen et al. [56] and Chang et al. [55] used ML and DL for precise antigen prediction and RH genotyping, enhancing transfusion safety. Operational studies showed measurable system-level benefits: Lou et al. [46] demonstrated that GB reduced type-and-screen orders by 33% across more than four million procedures, and Li et al. [58] reported that XGB-based forecasting cut RBC inventory by 38% and costs by 43%. Collectively, these findings underscore AI’s ability to deliver both clinical and operational gains, reinforcing its potential to transform PBM practice.

The comparative boxplots provide an integrated view of model performance across PBM domains. By restricting the analysis to the best-performing model from each study, the figures illustrate the upper bound of achievable AUC. In anemia detection, DL models—tested in nearly equal numbers to ML—consistently clustered above 0.95, confirming their robustness in image-rich tasks. In bleeding prediction, the predominance of ML studies explains the wider variability observed, while DL and hybrid approaches reached higher and more stable medians. In transfusion prediction, DL again outperformed ML, with only a single hybrid model achieving best performer status. For other fields, where study numbers were modest, no hybrid model was the top performer. Taken together, these distributions show that DL and hybrid methods more often achieved the highest and most consistent AUCs, whereas ML displayed broader variability, reflecting both methodological diversity and their numerical predominance across domains. This predominance of ML, despite higher AUCs reported for DL and hybrid in some domains, likely reflects its simplicity, lower computational requirements, and greater interpretability. In addition, ML approaches were introduced earlier in PBM research, leading to a larger accumulated body of studies compared with newer DL and hybrid methods.

Despite these advances, several limitations were consistently observed. Many models were developed on relatively small datasets or highly imbalanced case–control groups, limiting generalizability. Examples include anemia detection in a rare cohort [65,66] and bleeding prediction in orthopedics, where only 293 bleeding cases were reported among >35,000 procedures [67]. Postpartum hemorrhage studies also often involved <2% prevalence, reducing model stability. Larger, multicenter datasets are needed to ensure representativeness, particularly for low-prevalence outcomes.

Validation strategies also limited confidence in many studies. A large proportion relied solely on internal hold-out or k-fold cross-validation [17,18,19]. While these methods mitigate overfitting, they cannot replace independent testing. Stronger designs incorporated external validation, such as Khan et al. [16] for retinal imaging in anemia, Shi et al. [45] for perioperative bleeding, Zapf et al. [47] for transfusion forecasting, and Abbasi et al. [43] for cardiothoracic bleeding. Systematic external and temporal validation should become standard practice.

Benchmarking was also inconsistent. Several models were reported without comparison to LR or established clinical tools, limiting interpretability. Where such comparisons were made, AI consistently outperformed conventional approaches. Notable examples include Mortazavi et al. [36] and Hamilton et al. [37] against NCDR scores, Shung et al. [51] against Rockall and AIMS65, and Mora et al. [63] against RIETE and VTE-BLEED. Ensuring consistent benchmarking against LR and specialty-specific tools will be crucial for clinician confidence.

Performance reporting was heterogeneous. Some studies reported only accuracy, which can be misleading in imbalanced datasets, while others provided AUC, sensitivity, specificity, or F1-score. Calibration, essential for clinical adoption, was rarely assessed; few studies reported Brier scores. Adoption of transparent reporting of a multivariable prediction model for individual prognosis or diagnosis/AI and the prediction model risk-of-bias assessment tool/AI reporting standards will improve transparency and comparability.

Almost all studies were retrospective. Only a few incorporated prospective validations, such as Mora et al. [63] in anticoagulated patients and Almeida et al. [66] in cytomorphology. Furthermore, most papers stopped at model development and did not explore integration into workflows, EHR, or transfusion services. Prospective, pragmatic trials and pilot implementations are urgently needed to demonstrate real-world utility, workflow feasibility, and clinician acceptance.

Explainability was another recurring gap. DL models applied to imaging and time-series data often lacked interpretable outputs. Few applied SHapley Additive exPlanations or Local Interpretable Model-agnostic Explanations to identify drivers of predictions. For PBM, where decisions carry immediate transfusion consequences, explainability is critical for clinician trust and regulatory approval.

Taken together, these limitations highlight both the promise and immaturity of AI in PBM. Addressing them will require larger, more representative datasets, systematic external and prospective validation, consistent benchmarking against established tools, and greater emphasis on explainability and workflow integration. Future research should also explore multimodal and federated learning to improve generalizability while preserving data privacy, and cost-effectiveness analyses to establish system-level value. By advancing along these lines, AI can evolve from proof-of-concept models into clinically embedded tools that enhance safety, efficiency, and sustainability in PBM.

## 5. Limitations of This Review and Conclusions

This review has several limitations that should be acknowledged. Although we conducted comprehensive searches in PubMed, Scopus, and Web of Science, relevant studies indexed elsewhere or unpublished work may have been missed, introducing potential publication bias. Only studies published in English, Portuguese, and Spanish were included, which may have excluded evidence in other languages. While we systematically extracted study characteristics and performance metrics, heterogeneity in reporting limited direct comparisons across studies. For example, some reported only accuracy, while others provided AUC, sensitivity, specificity, or calibration metrics, precluding formal meta-analysis or pooled synthesis. In addition, our scope was limited to studies that explicitly reported AI applications in PBM; related work in adjacent domains may not have been captured. Finally, although we applied structured search and screening methods, this was not a formal systematic review, and risk-of-bias assessments were conducted qualitatively rather than with standardized scoring tools. Although AI demonstrates a consistently strong retrospective performance across PBM domains, the absence of prospective clinical validation remains a critical limitation. Most models have not yet been assessed in real-time clinical settings, and their performance in smaller or heterogeneous local cohorts is largely unknown. These gaps restrict the immediate translational impact of current AI applications in PBM and highlight the need for multicenter, prospective, and workflow-integrated evaluations before clinical adoption.

Despite these limitations, this review provides the most comprehensive and up-to-date synthesis of AI applications in PBM to date. Across anemia detection, bleeding risk stratification, transfusion forecasting, decision support, and blood bank operations, AI models frequently outperformed LR, established clinical scores, and even expert judgment. Large-scale studies with external validation demonstrated strong generalizability, and several reports showed tangible system-level benefits such as reduced type-and-screen orders, lower inventory requirements, and cost savings. At the same time, current evidence remains heterogeneous, limited by small or imbalanced datasets, inconsistent validation, and scarce prospective evaluation. Future research should prioritize multicenter and prospective studies, standardized reporting, explainability, and integration into clinical workflows. By addressing these challenges, AI has the potential to transform PBM into a more precise, efficient, and sustainable practice, improving both patient outcomes and resource stewardship.

## Data Availability

All data analyzed in this systematic review were extracted from previously published studies. The full list of included articles and extracted variables is provided in the Supplementary Materials (Tables S1–S5). No new datasets were generated. Additional information is available from the corresponding author upon reasonable request.

## Supplementary Materials

The following supporting information can be downloaded at https://www.mdpi.com/article/10.3390/jcm14238479/s1, Table S1: Optimization of red cell mass [16,17,18,19,20,21,22,23,24,25,26,27,28,29,30,31,32,33,34,35,65,66,68,69,70,71,72,73,74,75,76,77,78,79,80,81,82,83,84,85,86,87,88,89,90,91,92,93,94,95,96,97,98,99,100,101,102,103,104,105,106,107,108,109,110,111,112,113,114,115,116,117,118,119,120,121,122,123,124,125,126,127,128,129,130,131,132,133,134,135,136,137,138,139,140,141,142,143,144,145,146,147,148,149,150,151,152,153]; Table S2: Minimization of blood loss and bleeding [36,37,38,39,40,41,42,43,44,45,63,64,67,154,155,156,157,158,159,160,161,162,163,164,165,166,167,168,169,170,171,172,173,174,175,176,177,178,179,180,181,182,183,184,185,186,187,188,189,190,191,192,193,194,195,196,197,198,199,200,201,202,203,204,205,206,207,208,209,210,211,212,213,214,215,216,217,218,219,220,221,222,223,224,225,226,227,228,229,230,231,232,233,234,235,236,237,238,239,240,241,242,243,244,245,246,247,248,249,250,251,252,253,254,255,256,257,258,259,260,261]; Table S3: Harness and optimize physiological reserve of anemia [37,46,47,48,49,50,211,222,237,262,263,264,265,266,267,268,269,270,271,272,273,274,275,276,277,278,279,280,281,282,283,284,285,286,287,288,289,290,291,292,293,294,295,296,297,298,299,300,301,302,303,304,305,306,307,308,309,310,311,312,313,314,315,316,317,318,319,320,321,322,323,324,325,326,327,328,329]; Table S4: Other fields of indication [51,52,53,54,55,56,57,58,59,60,61,62,330,331,332,333,334,335,336,337,338,339,340,341,342,343,344,345,346,347,348,349]; Table S5: TabList of Abbreviations; PRISMA Checklist [349,350,351,352,353,354,355,356,357,358,359,360,361,362,363,364,365,366,367,368,369,370,371,372,373,374,375,376,377,378,379,380,381,382,383,384,385,386,387,388,389,390,391,392,393,394,395,396,397,398,399,400,401,402,403,404,405,406,407,408,409,410,411,412,413,414,415,416,417,418,419,420,421,422,423,424,425,426,427,428,429,430,431,432,433,434,435,436,437,438,439,440,441,442,443,444,445,446,447,448,449,450,451,452,453,454,455,456,457,458,459,460,461,462,463,464,465,466,467,468,469,470,471,472,473,474,475,476,477,478,479,480,481,482,483].
