# Supplementary material for: Artificial Intelligence in Patient Blood Management: A Systematic Review of Predictive, Diagnostic, and Decision Support Applications
Source: J Clin Med. 2025 Nov 29;14(23):8479. doi: 10.3390/jcm14238479 (PMC12693012; doi:10.3390/jcm14238479)
Supplement: Supplementary file 1 [file jcm-14-08479-s001.zip › jcm-3968312-supplementary.pdf]

Table S1. Optimization of Red Cell Mass.

Reference (Year)	PBM Application	Sample Size (case group)	Model Variables Top Predictors (bold)	Models	Model Validation	Key Findings
**Anemia detection and prediction**						
Acharya *et al*. (2020)[68]	Non-Invasive estimation of Hb	1,583 women<br><br>(NA)	Demographics: age, pregnancy status. Others: PPG signals at four wavelengths (590 nm, 660 nm, 810 nm, and 940 nm).	Stacking Regressor (combining LASSO, RidgeR, EN, AdaBoost, SVR)	Hold-out<br>Training: 80%<br>Testing: 20%<br><br>5-fold CV	Stacked regressor (PCC = 0.81, RMSE = 1.353 g/dL) outperformed individual models, achieving high accuracy for non-invasive Hb estimation with minimal misclassification risk (1.78%).
Aghajanian *et al*. (2024)[69]	Predict post-delivery Hb levels	1,974 patients<br><br>(NA)	**Demographics:** age, BMI. Clinical: **gestational age**, gravidity, **parity**, SAH, GHT, GDM, previous deliveries. Procedure: progesterone use, delivery indication and type, placenta orientation. Lab: **pre-labor Hb**, **PLT**, **FIB**.	ANN, LinearR, SVM, XGB, RT, Ensemble models	Hold-out<br>Training: 80%<br>Testing: 20%<br><br>5-fold CV	ANN provided the best predictive performance (RMSE = 0.62).
AlAgha *et al*. (2018)[70]	Identifying β-thalassemia carriers	45,498 participants<br><br>(1,138 with β-thalassemia carriers)	Complete blood count parameters: RBC, Hb, HCT, MCV, MCH, MCHC, RDW, PLT, WBC.	KNN, DT, MLP, NB	10-fold CV	NB performed best (sensitivity 98.81% and specificity 99.47%).
Alagu *et al*. (2022)[33]	Detect sickle cell anemia in RBCs	900 blood smear images<br><br>(450 with sickle cells)	Deep features (n=2048) extracted from InceptionV3; feature subset selection via MO-BGWO; final input: optimized feature vectors for classification.	InceptionV3, SVM, KNN	Hold-out<br>Training: 80%<br>Testing: 20%<br><br>4-fold CV for KNN; trial-and-error	SVM + MO-BGWO and Gaussian kernel achieved the highest accuracy (96%) and outperformed InceptionV3 classifier (91%), showing superior

					tunning for SVM; no mention for DL.	precision, sensitivity, specificity, and F1-score.
Almeida *et al*. (2023)[66]	Detect disease-associated cytomorphologies and predict hematological diseases	362 blood smear images<br><br>(304 with MDS+ megaloblastic anemia + IDA)	Lab: **WBC**, **Hb** and **PLT** count; WBC and **RBC** morphometric features (**cell area**, perimeter, major and minor axis lengths, eccentricity, aspect ratio, circularity, convexity, solidity, **centroid distance variability**), color intensity, texture features, nuclear-specific features for **WBC** (nuclear area, **perimeter**, **convexity**, solidity, eccentricity, and centroid distance variability).	DL: DenseNet-121, U-Net (for quality control and cell segmentation). ML: XGB, EN (for RBC filtering and disease prediction), custom Morphotype Analysis (for cytomorphology discovery).	Hold-out<br>Training: 80%<br>Testing: 20%<br><br>5-fold CV<br><br>EV (n=63)	Morphotype Analysis and EN Regression were the best models (AUC ~0.90 in internal and 0.86-0.90 in EV).
Amendolia *et al*. (2002)[71]	Detect thalassemia carriers	304 participants<br><br>(44 α-carriers, 9 β-carriers)	Lab: Hb, HCT, RBC, MCV.	ANN	Hold-out<br>Training: ~65%<br>Testing: ~35%<br><br>Leave-One-Out CV	**ANN** achieved **94% accuracy, 92% sensitivity,** and **95% specificity**, outperforming the traditional MCV <77 rule.
An *et al*. (2021)[65]	Detect anemia and Hb variants using microchip electrophoresis	46 participants<br><br>(37 with anemia and /or SCD)	Relative intensity time series extracted from raw image videos of microchip electrophoresis.	ANN	Hold-out<br>Training: 31.5%<br>Testing: 63%<br>Validation: 5.5%	ANN achieved 0.55 g/dL MAE for Hb, 100% sensitivity, 92.3% specificity for anemia, and 100% accuracy for SCD detection.
Appiahene *et al*. (2023)[72]	Detect anemia in children using conjunctiva images	710 images<br><br>(424 anemic)	**Image-derived visual features** — such as color intensity, texture, shape, and shading patterns.	VGG16, ResNet50, DenseNet121, Vision Transformer, ConvNeXtBase	5-fold CV	ViT achieved best AUC (0.841) for classification and lowest MAE (1.50 g/dL) for Hb estimation.
Appiahene *et al*. (2023)[18]	Detect anemia using images of children's palm	710 children	RBG pixel palm images. Demographics: age, sex. Lab: Hb.	RF, DT, NB, SVM, ANN, Ensemble	Hold-out<br>Training: 70%<br>Testing: 20%	Stacking Ensemble (NB + RF) achieved 99.98% accuracy, 100% specificity,

		(not explicitly stated)		(Stacking, Voting, Bagging, Boosting)	Validation: 10%<br><br>5-fold CV	precision, recall, F1 score, and an AUC of 1.0).
Appiahene *et al*. (2023)[73]	Detect IDA using images of children's palm	527 images<br><br>(304 anemic)	CIELAB color space values (L*, a*, b*), RGB values.	CNN, KNN, NB, SVM, DT	Hold-out<br>Training: 70%<br>Testing: 20%<br>Validation: 10%<br><br>10-fold CV	NB achieved the highest accuracy (99.96%).
Arjmand *et al*. (2010)[74]	Detect thalassemia	28 participants<br><br>(15 with thalassemia)	Component scores derived from preprocessed ^{1}H NMR spectra of blood serum.	PCA+LDA	Leave-One-Out CV	PCA + LDA diagnosed thalassemia with high accuracy, correctly classifying all test samples in three of four sets and misclassifying only one sample overall.
Asare *et al*. (2023)[19]	Detect IDA using fingernails, palm and conjunctiva images	710 images<br><br>(424 anemic)	CIELAB color space values (L*, a*, b*), RGB values.	NB, CNN, SVM, KNN, DT	Hold-out<br>Training: 70%<br>Testing: 20%<br>Validation: 10%<br><br>10-fold CV	CNN outperformed other models (accuracy 99.12%); palm was the most reliable body region for anemia detection.
Asare *et al*. (2024)[75]	Detect IDA in children using conjunctiva images	2635 images<br><br>(1824 with anemia)	CIELAB color space values (L*, a*, b*), RGB values. Demographics: age, sex. Lab: Hb.	CNN, NB, KNN, SVM, DT	Hold-out<br>Training: 70%<br>Testing: 20%<br>Validation: 10%<br><br>10-fold CV	CNN achieved the highest performance (accuracy 98.45%, AUC 0.999)
Ayyıldız *et al*. (2020)[76]	Distinguish IDA from β-thalassemia	342 patients	Demographics: gender. Lab: RBC, Hb, **HCT**, **MCV**, MCH, **MCHC**, **RDW**.	SVM, KNN	**k-fold cross-validation**	KNN achieved the best classification performance (accuracy 95.3%, F1 score 0.957).

		(190 with IDA, 152 with β-thalassemia)				
Azarkhish *et al*. (2011)[77]	Diagnose IDA and predict serum iron levels	203 patients<br><br>(99 with IDA)	Demographics: age. Lab: RBC, **MCV**, **MCH**, **MCHC**, **Hb-to-RBC ratio**.	ANN, ANFIS, LR	Hold-out<br>Training=73%<br>Testing=27%	ANN performed best for diagnosing IDA, achieving 96.3% accuracy and an AUC of 0.982.
Banerjee *et al*. (2023)[21]	Estimate Hb in neonates	1,935 neonates<br><br>(65 anemic)	Whole optical spectrum data from neonatal nail bed, processed spectral signals, absorbance at specific wavelengths for Hb.	ANN	Hold-out<br>Training: **~48%**<br>Testing: ~52%	ANN showed strong correlation with standard lab tests (r = 0.987 for Hb estimation).
Barnhart-Magen *et al*. (2013)[78]	Differentiate thalassemia minor from other blood disorders (IDA and MDS)	526 patients<br><br>(185 with **α- or β-**thalassemia minor)	Lab: Hb, **MCV**, **RDW**, **RBC**, MCH, PLT.	ANN	Not explicitly stated	ANN achieved 96.7% specificity and 100% sensitivity for diagnosing thalassemia minor versus controls and MDS, and 90% sensitivity when distinguishing it from IDA.
Chakraborty *et al*. (2022)[79]	Estimation of clinical severity of anemia	1,516 women<br><br>(1,091 anemic)	13 features derived from photoplethysmographic signals, including 9 ratio-of-ratios between wavelength pairs and 4 attenuation values.	Ensemble classifier (SVM, LR, RF, boosted tree)	Hold-out<br>Training: 70%<br>Testing: 15%<br>Validation: 15%<br><br>30-fold Monte Carlo CV	Ensemble model detected anemia with 92%/84% sensitivity/specificity and severe anemia with 76%/74%.
Chen *et al*. (2016)[80]	Detect anemia in conjunctiva images	100 images<br><br>(50 from patients with anemia)	Imaging: Pixel Value in the Middle from Green channel, Binarized High Hue Ratio, entropy.	SVM, ANN	10-fold CV	SVM achieved the best performance (sensitivity 78%, specificity 83%, Kappa 0.61).

Chen *et al*. (2019)[81]	Detect anemia in retinal vessel OCT images	571 images (from 40 patients)<br><br>(316 images from 24 patients with anemia)	**Image-based features extracted from retinal OCT images, including statistical features (m**ean gray value, mode of gray value, mean of retinal nerve fiber layer, blood vessel brightness and shadow) and **texture features (l**ocal binary pattern, histogram-based features, entropy measures).	LDA	Leave-One-Out CV	LDA classifier achieved 83.58% accuracy.
Chen *et al*. (2022)[82]	Detect anemia in conjunctiva images	1,065 patients<br><br>(not explicitly stated)	**Palpebral conjunctiva image segmentation, color features (High Hue Ratio, Green Channel Intensity), texture and brightness features (Histogram-based Features, LBP Features), medical Indicator (Hb level).**	DT, LinearR, SVM, KNN, RFR, BTR, CNN (Mask R-CNN, MobileNetV3+SE, MobileNetV2, ShuffleNetV2, SqueezeNet, ResNet-CBAM, B-CNN)	Hold-out<br>Training: 80%<br>Testing: 20%<br><br>10-fold CV	MobileNetV3+SE performed best (R^2 = 0.512, EVS = 0.535, MAE = 1.521).
Christensen *et al*. (2025)[31]	Identifying *α*-thalassemia carriers	201 individuals<br><br>(carriers: 146; non-carriers: 55)	**Demographics: sex. Lab: Hb, HCT, RBC, MCV, MCH, MCHC, RDW, WBC, NEU%, LYM%, PLT, HbA, HbA2, HbF.**	LR, KNN, SVC, NB, MLP, DT, XGB, RF, CNN	Hold-out<br>Training: 85%<br>Testing: 15%	CNN achieved the highest performance (AUC 0.95, sensitivity 0.80, and specificity 0.86); ML models consistently outperformed classical discriminant formulas.
Çil *et al*. (2020)[83]	Discrimination of β-thalassemia trait and IDA	342 patients<br><br>(152 with β-thalassemia trait; 190 with IDA)	Demographics: gender. Lab: Hb, HCT, RBC, MCV, MCH, MCHC, RDW.	ELM, RELM, SVM, KNN, LR	Hold-out<br>Training: 70%<br>Testing: 30%	**RELM achieved the highest classification accuracy (95.59% for mixed gender, 96.3% for females).**
Çuvadar *et al*. (2023)[84]	Hb estimation from conjunctival images	388 participants	Demographics: age, gender, height, weight, BMI. Image	CNN+MLP	Not explicitly stated	CNN+MLP achieved a R^2 = 0.86, RMSE = 0.68 g/dL,

		(not explicitly stated)	data: conjunctival images captured via smartphone camera.			MAPE = 3.4%, and bias = 0.26 g/dL.
Darshan *et al*. (2024)[85]	Predict anemia based on hematological markers	2,039 participants<br><br>(not explicitly stated)	Demographics: **gender**. Lab: Hb, RBC, **HCT**, **MCV**, MCHC, **MCH**, RDW, **SDRDW**, **TSD**, **SDTSD**, WBC, NEU, LYM, MO, EOS, BASO, PLT, MPV, PCT, PDW, **VitB12**, **folate**, **ferritin**.	LR, DT, RF, KNN, CatBoost, XGB, Stacked Ensemble	Hold-out<br>Training: 80%<br>Testing: 20%	Stacked model and XGB delivered better overall performance with 99% accuracy, precision, and recall, and an AUC of 0.97.
Darshan *et al*. (2024)[86]	Detect anemia	364 patients<br><br>(208 anemic)	Demographics: age, **sex**. Lab: **RBC**, MCV, MCH, **MCHC**, RDW, PLT, **Hb**, **HCT**, WBC.	RF, KNN, SVM, NB, XGB, CatBoost, ANN	Hold-out<br>Training: 80%<br>Testing: 20%	KNN and XGB achieved the highest accuracy (98%), but RF had highest recall (98%).
Darshan *et al*. (2025)[87]	Distinguishing IDA from AA	500 patients<br><br>(IDA: 266; AA: 234)	Demographics: **age**, gender. Lab: **Hb**, **HCT**, **MCV**, **MCH**, **MCHC**, **RBC**, RDW, **WBC**, **PLT**, **PDW**, MPV, **NEU**, **LYM**, EOS, **procalcitonin**.	LR, RF, DT, KNN, AdaBoost, XGB, CatBoost, LGBM, ANN, Stacked Ensemble	Hold-out<br>Training: 80%<br>Testing: 20%<br><br>5-fold CV	**LGBM and Stacked Model achieved the highest accuracy (96%).**
Das *et al*. (2022)[88]	Non-invasive β-thalassemia trait screening in antenatal women	2,942 women<br><br>(665 with β-thalassemia trait)	Demographics: age, ethnicity. Lab: **Hb**, **MCV**, **MCH**, **RBC**, **RDW**.	RF, SVM, LR, KNN, DTC, ETC, BaggingC, MLP, LDA, AdaBoost, GBC, ELM	Hold-out<br>Training: 70%<br>Testing: 30%	**ELM and GBC were the top-performing models (AUC 0.920),** with ELM having the highest Youden's Index (0.838).
Das *et al*. (2023)[22]	Hb estimation from nail-bed color changes	220 participants<br><br>(158 anemic)	Demographics: age, gender. Deoxygenation and reoxygenation times and slopes, their ratios, red channel intensity values from key video frames.	MLR, PR, SVR, RidgeR, BRR, LASSOR, SGDR, GBR, ENLR, LGBMR, KNNR, DTR, RFR, MLP, Fusion models (MLP+ML regressors)	10-fold CV and Leave-One-Out CV	Fusion-1 model performed best (RMSE: 0.63, MSE: 0.61, Bias: 0.01); Sensitivity: 0.97, Specificity: 0.68.
Das *et al*. (2024)[89]	Detect SCD	196 images<br><br>(NA)	Pixel data from microscopic images, including **saturation channel**; class labels for five	ACDSSNet (model based on modified DeepLabV3+, with	10-fold CV	ACDSSNet achieved a high performance (accuracy 98.21%,

			cell types: DSO, RGE, Sickle cell, Elongated, Background (including non-sickle cells, WBCs, PLTs, and image background).	MobileNetV2/ResNet50 as feature extractors)		specificity 99%, Dice Similarity Coefficient 0.9547).
Dejene *et al*. (2022)[90]	Predict the level of anemia among pregnant women	29,104 women<br><br>(~9,111 anemic)	**Demographics: age**, **household members**, region, place of residence, **occupation**, **partner's education level**, **wealth index**, religion, BMI. Clinical: history of pregnancy, history of **contraceptive use**, history of abortion, iron and vitamin intake, drug for malaria use. Others: **source of drinking water**, wealth index, occupation, antenatal care visits.	RF, XGB, DT, CatBoost	Hold-out<br>Training: 80%<br>Testing: 20%<br><br>K-fold CV	CatBoost achieved the best performance (accuracy 97.6%. precision 97.6%).
Diaz-del-Pino *et al*. (2023)[91]	Detection of hematological diseases (including several types of anemia)	4,061 hemograms<br><br>(not explicitly stated)	**Demographics: age, sex. Lab: Hb, HCT, MCV, MCH, MCHC, RBC, WBC, NEU%,** LYMP% MO%, EOS%, BASO%, PLT, MPV, RDW, Large unstained cells%, Immature granulocytes%, nucleated RBC%.	RF, SVM, XGB, TabNet, BloodNet	5-fold CV	**BloodNet performed best (accuracy 96.4%, precision 98%, recall 96%, AUC 0.993).**
Dimauro *et al*. (2023)[92]	Detect anemia in eye conjunctiva images	221 images<br><br>(42 anemic)	Demographics: age, sex. Features from conjunctiva images: RGB color means, hue, brightness, entropy, texture features.	RUSBoost, RF, SVM, KNN	Hold-out<br>DL Models:<br>Training: 70%<br>Testing: 15%<br>Validation: 15%<br><br>Other models:<br>Training: 70%	**RUSBoost performed best** (sensitivity = 0.66–0.79, specificity = 0.74–0.91).

					Testing:30%	
Douglass *et al*. (2022)[34]	Identification of SCD	36,000 images<br><br>(18,000 with sickle cell disease)	**Opto-biological signatures** from a CMOS image sensor, transformed using **Local Binary Pattern preprocessing** to enhance classification.	CNN (AlexNet, VGG19, ResNet-50, SqueezeNet)	3-fold CV	**AlexNet with Local Binary Pattern preprocessing** achieved the best classification performance (accuracy **88.7%, AUC 0.96)**
Erten *et al*. (2022)[93]	Discrimination of β-thalassemia and IDA	First dataset: 159 participants<br><br>(53 IDA; 53 β-thalassemia)<br><br>Second dataset: 1,883 participants<br><br>(264 IDA; 47 β-thalassemia)	Hb, **MCV**, **MCH**, **Ferritin**, **UIBC**, RDW, **HbA2**, HbF.	MGSVM, SVM, CoarseT, DT, RF, KNN, ANN, LR, Discriminant Analysis, NB	Hold-out<br>various split ratios tested (90:10, 80:20, 70:30, 60:40, 50:50)<br><br>10-fold CV	MGSVM was the best-performing classifier for the balanced dataset (97.48% accuracy, 97.53% F1-score), while the CT classifier achieved the highest accuracy for the larger, imbalanced dataset (99.73%).
Feng *et al*. (2022)[32]	Identifying **α**-thalassemia carriers	1,213 patients<br><br>(497 carriers)	Demographics: pregnancy status. Lab: **RBC,** HCT, **MCV**, **RDW**.	RF, DT, KNN, LDA, SVM, LinearSVC, GB, SGD, Bernoulli NB, Gaussian NB, AdaBoost, XGB, MLP, LR	Hold-out<br>Testing: 70%<br>Training: 30%<br><br>EV (n=399)	RF identified alpha-thalassemia carriers with an AUC of 0.957, 87.9% sensitivity, and 92.1% specificity, raising PPV from 41% to 94% and cutting genetic testing needs by 60%.
Foy *et al*. (2023)[94]	Quantitation of RBC morphology to assist in diagnosis and improve clinical decision-making	338,557 images<br><br>(not explicitly stated)	**RBC features** (area, perimeter, circularity, solidity, aspect ratio, convexity).	SVM	Hold-out<br>Training: 67%<br>Testing: 33%<br><br>EV (n=56,832)	SVM accurately quantified RBC morphologies with a mean AUC of 0.93, strong expert correlation (R^2 = 0.76), and improved thrombotic

						microangiopathies diagnostic specificity.
Fu *et al*. (2024)[95]	Screening of anemia and thalassemia	297 samples<br><br>(not explicitly stated)	**Chromatographic Data:** Peak patterns of hemoglobin species (HbA1c, HbA0, HbF, HbA2, and total Hb).	ResNet1D	Hold-out<br>Training: 70%<br>Testing: 20%<br>Validation: 10%	ResNet1D achieved >90% accuracy and >96% sensitivity for each disease; significantly outperformed the mIEF-only method (69.33% accuracy for multiple diseases).
Garduno-Rapp *et al*. (2024)[25]	Predict IDA before conventional diagnosis	30,603 patients<br><br>(7,850 with IDA)	Hb, Ferritin, TSAT, Fe, TIBC, UIBC, MCH, MCHC, MCV, RDW, HCT, RBC, PLT, WBC, NEU, LYMP, MO, EOS, BASO, blasts, myelocyte, metamyelocyte, MPV, Retic, ALB, bil, ALP, ALT, AST, creatinine, BUN, Ca, K, Na, Cl, AG, TP, RLYM, glucose.	ANN, LSTM, GRU	Hold-out<br>Training: 60%<br>Testing: 20%<br>Validation: 20%<br><br>5-fold CV	GRU performed best (AUC 0.89, accuracy 83%, sensitivity 75%, and specificity 85%) and predicted IDA up to 6 months prior to conventional diagnosis using lab data.
Göl *et al*. (2025)[96]	Predict anemia in geriatric patients	404 patients<br><br>(not explicitly stated)	Demographics: age, **gender**. Clinical: **smoking**, alcohol use, DM, SAH, hyperlipidemia, cardiovascular disease, thyroid disorders, Parkinson's disease, rheumatologic diseases, gastrointestinal disorders, asthma, hematologic diseases, Alzheimer's disease, epilepsy, depression, osteoporosis. **Nutrition and lifestyle factors** and **physical activity measures**. Hemogram and biochemistry tests.	J48 DT, RF, NB, BayesNet, Decision Table, DTNB	10-fold CV	J48 DT performed best (accuracy **97.77%, precision 0.98, AUC of 0.98).**

Hennek *et al*. (2016)[97]	Enhance IDA diagnosis using AMPS and ML	152 blood samples<br><br>(not explicitly stated)	**Image-derived red intensity features** from AMPS tests — a visual proxy for RBC density distribution.	LR	Monte Carlo CV	Combining AMPS with LR improved IDA diagnosis to an AUC of 0.90, with 90% sensitivity and 77% specificity.
Jain *et al*. (2020)[98]	Detect anemia from eye conjunctiva	3202 images<br><br>(not explicitly stated)	Mean red (μr) and green (μg) pixel intensities from the conjunctival region of eye images.	ANN	Hold-out<br>Training: 60%<br>Testing: 20%<br>Validation: 20%	ANN achieved 97% accuracy, 99.21% sensitivity, and 95.42% specificity.
Kabootarizadeh *et al*. (2019)[99]	Discriminate β-thalassemia trait and IDA	268 patients<br><br>(148 with IDA; 120 with β-thalassemia trait)	Lab: RBC, Hb, MCV, MCH.	MLP	Hold-out<br>Training: 70%<br>Testing: 15%<br>Validation: 15%	MLP outperformed traditional indices, achieving 92.5% accuracy, 93.1% sensitivity, and 92.3% specificity.
Kato *et al*. (2024)[100]	Predict Hb level in conjunctiva images	150 patients (300 images)<br><br>(1**0 anemic)**	RGB values of palpebral conjunctival images (non-CNN model); CNN-extracted image features (**lower half and marginal edges of the conjunctiva**); exposure time; aspect ratio.	CNN, MLR	5-fold CV	CNN outperformed MLR in predicting continuous Hb values (r = 0.45 vs. 0.38), while MLR showed better anemia detection (AUC = 0.82 vs. 0.74).
Kay *et al*. (2023)[101]	Detect anemia on pulmonary CT angiography	100 patients<br><br>(50 with anemia)	Demographics: age, gender, BMI. Lab: Hb, Imaging: CT attenuation in different cardiovascular locations (right atrium, **right ventricle**, left atrium, **left ventricle**, ascending aorta, **descending aorta**, pulmonary artery, aortic arch).	XGBT	Hold-out<br>Testing: 76%<br>Training: 24%<br><br>10-fold CV	XGBT achieved 83% sensitivity, 92% specificity, and 88% accuracy.

Khan *et al*. (2025)[16]	Detect anemia and estimation of Hb level in retinal images	5,830 images (2,265 participants)<br><br>(not explicitly stated)	Demographics: age, gender. Lab: Hb. Retinal fundus images (**vessel density**, **vessel tortuosity**, vessel thickness).	CNN (VGG16, ResNet50, InceptionV3)	Hold-out<br>Training: 80%<br>Testing: 20%<br><br>EV (n=255 images)	InceptionV3 achieved 98% accuracy, 99% sensitivity, 97% specificity, and an AUC of 0.98 for anemia detection, while for Hb estimation, it had a MAE of 0.58 g/dL.
Kim *et al*. (2019)[102]	Screening hematologic disorders (IDA, RETIC, HS, and DM)	1,028 RBCs<br><br>(IDA:99, HS:86, RETIC:166, DM:240)	**Morphological** (Sphericity, Surface Area), Chemical (Hb Content, Hb Concentration), **Mechanical** (Membrane Fluctuation).	NN	Each disease group had 40 randomly selected RBCs as test set; remainder used for training	**NN achieved >98% classification accuracy** for hematologic disorders.
Kitaw *et al*. (2024)[103]	Predict anemia in pregnant women	11,174 women<br><br>(10,882 anemic)	**Demographics: a**ge, region, residence, **wealth status**, **education**, occupation, household size, water source, toilet type**, mosquito net**, religion, media access (TV, **frequency of reading newspapers**), marital status, **BMI**. **Clinical: p**regnancy duration, birth history, **termination history**, ANC visit frequency. **Lab:** Hb, Anemia classification (severe, moderate, mild, non-anemic).	MLPNN, XGB, NB, DT, RF, KNN	Hold-out<br>Training: 80%<br>Testing: 20%<br><br>K-fold CV<br><br>EV (n not provided)	RF performed best (accuracy 97%).
Kurstjens et al. (2022)[104]	Predict ferritin levels in anemic patients	12,009 patients<br><br>(JBH-S: 3,797 and ~285 with low ferritin; Medlon-R:	*Demographics: age, sex. Lab: Hb,* ***MCV****,* ***MCH****, RBC, PLT, WBC,* ***CRP****.*	RF<br>(2 versions: JBH-S and Medlon-R)	Hold-out<br>JBH-S<br>Training: 70%<br>Testing: 30%<br>Medlon-R<br>Training: 80%	RF outperformed lab specialists (AUC 0.90-0.92).

		8,201 and ~497 with low ferritin)			Testing: 20%<br><br>EV (n=191; ~19 with low ferritin)	
Kwon *et al*. (2020)[20]	Detect anemia by EKG	39,872 patients<br><br>(1,561 anemic)	Demographics: age, sex, BMI. Clinical: HR. Lab: Hb. EKG features (**QRS complex**, **T-wave axis**, QT interval, PR interval, **P-wave**).	LR, CNN, MLP	Hold-out<br>Training: 80%<br>Testing: 20%<br><br>EV (n=4,665)	CNN outperformed LR (AUC 0.923 internal, 0.901 external vs. 0.85).
Lachover-Roth *et al*. (2024)[28]	Detect α thalassemia carriers	1,334 blood count samples<br><br>(291 with α-thalassemia mutation)	Lab: RBC, Hb, **MCV**, **MCH**, MCHC, RDW, HbF, HbA2.	SVM	Not explicitly stated	SVM achieved 99.33% sensitivity and 99.93% negative predictive value; outperformed all 16 other formulas.
Laengsri *et al*. (2019)[105]	Discriminate thalassemia trait and IDA	186 patients<br><br>(40 with IDA and 146 with thalassemia trait)	Lab: **RBC**, **Hb**, HCT, MCV, MCH, **MCHC**, **RDW**.	KNN, DT, RF, ANN, SVM	Hold-out<br>Training: 80%<br>Testing: 20%<br><br>5-fold CV<br><br>EV (n=37; functioned as test set)	**SVM** outperformed all existing discrimination indices, achieving **95.59% accuracy, and AUC of 0.98.**
Lin *et al*. (2024)[106]	Non-Invasive detection of anemia	319 patients<br><br>(**~102 with anemia**)	Demographics: **age**, **gender**. Image: conjunctiva, **palm**, fingernail. **Body Part Tag**: one-hot vector (3D).	BPANet (ResNet50-based CNN model)	5-fold CV applied to the 218-patient retrospective dataset for IV; 4-fold CV used on the 101-patient prospective dataset for EV	BPANet achieved high performance (F1-score up to 0.828, accuracy 0.849).
Lobo *et al*. (2020)[26]	Predict Hb trends in ESRD patients	1,972 patients<br><br>(NA)	Demographics: age. Clinical: pre- and post-dialysis weight, time on dialysis, dialysis	LSTM	Hold-out<br>Training: 68%<br>Testing: 25%	LSTM had MAEs close to the clinical variability of

			session duration, number of dialysis sessions per week, **ESA** and **iron dose**. Lab: **ALB**, Ca, CO_2, **creatinine**, ESR, **ferritin**, folate, eGFR, HbA1C, HCT, **Hb**, iron, K, MCV, Na, protein catabolic rate, P, **TSAT**, parathyroid hormone, INR, RDW, transferrin, urea reduction rate, vancomycin.		Validation: 17%	Hb measurements (around ±0.5 g/dL).
Martínez-Martínez *et al*. (2014)[107]	Predict Hb levels in HD	13,011 patients<br><br>(NA)	Clinical: **dry weight**, dialysis treatment parameters, **ESA dose**, **iron dose**. Lab: **Hb**, **CRP**, ALB, WBC, NEU, **inflammation index**, **ferritin**, **TSAT**.	LinearR, RT, Bagging, SVM, MLP	Hold-out<br>Training: 66%<br>Testing: 34%	Bagging, SVM, and MLP performed best with MAE around 0.66 g/dL.
Masala *et al*. (2013)[108]	Differentiate normal subjects, α- and β-thalassemia carriers	304 participants<br><br>(α- and β-thalassemia carriers: 81/37)	Lab: **RBC**, Hb, HCT, **MCV**, **HbA2**.	RBF, PNN, KNN	Hold-out<br>Training: ≈64.5%<br>Testing: ≈35.5%	RBF achieved 100% accuracy for β-thalassemia and 93% specificity, 91% sensitivity for α-carriers.
Matović *et al*. (2021)[109]	Detect anemia by NIRS in HD	35 patients<br><br>(NA)	NIRS data from spent dialysis fluid and anemia-related blood parameters: Hb, HCT, RBC, Fe, TIBC, **Ferritin**, MCH, MCHC, MCV.	ANN	Hold-out<br>Training: 85%<br>Testing: 15%	ANN using NIRS data demonstrated high accuracy, achieving R > 0.90 for all anemia-related parameters.
Meitei *et al*. (2022)[110]	Predict child anemia	21,000 children<br><br>(10,460 anemic)	Demographics: **religion**, **caste/tribe**, **wealth index**, residence state and location. Child's characteristics: **age**, sex, size at birth, breastfeeding status, recent illness. Maternal factors: **age**, **education level**, **anemia** status, BMI. Others:	Ridge, LASSO, EN	Hold-out<br>Training: 80%<br>Testing: 20%	LASSO performed best (accuracy 64.3%, sensitivity 62.2%, specificity 66.3%, AUC >70%).

			household size, sanitation, drinking water safety, electricity access, type of cooking fuel, flooring material.			
Memmolo *et al*. (2022)[111]	Screening hereditary anemias	~8500 RBC images<br><br>(~7150 with hereditary anemias)	Morphological and phase-contrast features of RBCs: shape, volume, Hb content, membrane fluctuations, and holographic imaging parameters.	SVM, DT, KNN, Discriminant analysis, Ensemble methods	Hold-out<br>Training: ~90–93%<br>Testing: ~7–10%	SVM achieved 84.3% accuracy in distinguishing healthy from anemic RBCs and 69.5% accuracy in differentiating five hereditary anemia types.
Mitani *et al*. (2020)[24]	Detect anemia using eye conjunctival images	11,308 participants (114,205 images)<br><br>(439 with anemia)	**Fundus Images:** retinal features, **optic disc**, **blood vessel** structures, **macula**, **color** and spatial features. Metadata: **age**, **sex**, race, ethnicity, **smoking**, **BP, pulse rate**, height, weight, BMI.	CNN, LinearR, LR	Hold-out<br>Training: 70%<br>Testing: 20%<br>Validation: 10%	The combined model CNN (fundus images) + LinearR (metadata) performed better than either model alone, achieving an AUC of 0.88.
Mo *et al*. (2023)[112]	Predict thalassemia using RBC indices	8,693 instances<br><br>(4,454 with a positive genetic test for thalassemia)	Demographics: **age**, sex. Lab: RBC, Hb, MCV, MCH, MCHC, HCT, **RDW**, PLT, WBC.	DNN	10-fold CV	DNN outperformed MCV- and MCH-based screening, achieving AUC 0.960 and 10–11% higher accuracy.
Moreno *et al*. (2024)[113]	Detect anemia using eye conjunctival images	500 images<br><br>(not explicitly stated)	Image-derived descriptors (color channel stats, spatial coordinates).	SLIC-GAT, BR, SVR	Hold-out<br>Training: 70%<br>Testing: 30%<br><br>5-fold CV	SLIC-GAT achieved the lowest L1 error (0.7284 g/dL), outperforming ML methods.
Mulijono *et al*. (2024)[114]	Detect anemia using eye conjunctival images	500 images (4000 post-augmentation)	**Image-derived features** from conjunctival images of the eye.	SVM, MobileNetV2, MobileNetV2 + SVM	Hold-out<br>Training: 70%<br>Testing: 30%<br><br>5-fold CV	MobileNetV2 + SVM achieved 93% accuracy, 91% sensitivity, and 94% specificity, outperforming other methods.

		(Not explicitly stated)				
Muthalagu *et al*. (2018)[115]	Detect anemia using eye conjunctival images	127 images<br><br>(23 anemic)	**Image-derived color features from the conjunctiva,** including **mean RGB values**, **HSI color scores**, and **ratios of hue and saturation.**	FFNN, ENN	Not explicitly detailed	ENN achieved the best performance (**sensitivity** 77% and **specificity 96.11%).**
Muyama *et al*. (2024)[116]	Detect anemia using EHRs	70,000 synthetic EHRs<br><br>(60,000 with anemia)	Demographics: gender. **Lab: RBC**, **Hb**, HCT, **MCV**, **Retic**, segmented NEU, **ferritin**, iron, **TIBC**, TSAT, creatinine, cholesterol, copper, ethanol, folate, glucose.	DRL (DQN, DDQN, Dueling DQN, Dueling DDQN, DQN-PER, DDQN-PER, Dueling DQN-PER, Dueling DDQN-PER), DT, RF, SVM, XGB, FFNN	Hold-out<br>Training: 80%<br>Testing: 20%<br>Validation: 10%	**Dueling DQN-PER** achieved the **best performance** (accuracy 96.64, F1-score 96.5, AUC 0.98).
Navya *et al*. (2025)[117]	Detect IDA in peripheral blood smear images	386 images<br><br>(249 images with IDA)	Pixel-level image data: RBC morphology and size. DL feature extraction: RBC **shape** and **size**, **color variations**, spatial density and overlap.	DDOD-RetinaNet, YOLOv5, YOLOv7, YOLOv8, Faster R-CNN, RetinaNet	Hold-out<br>Training: 70%<br>Testing: 10%<br>Validation: 20%<br><br>5-fold CV	**DDOD-RetinaNet perfomed best (mAP@0.5:** 93.4%).
Pan *et al*. (2023)[118]	Predict IDA in premenopausal women one year after sleeve gastrectomy	407 women<br><br>(44 developed IDA)	**Demographics: age,** BMI, weight, waist/hip circumference. Clinical: SBP, DBP, SAH, DM, medications (antidiabetic, antihypertensive, lipid-lowering agents). Lab: WBC, **Hb**, ALT, AST, GGT, BUN, **creatinine**, uric acid, cholesterol, HDL, LDL, glucose, HbA1C, triglycerides, **fasting C-peptide** and insulin, folic acid, VitB12, iron, **ferritin**.	SVM	Hold-out<br>Training: 70%<br>Testing: 30%<br><br>10-fold CV	**SVM showed good performance (AUC=0.858 (training) and AUC 0.799 (validation).**

Pellicer-Valero *et al*. (2020)[27]	Predict Hb concentration in HD patients	110,758 patients<br><br>(NA)	**Demographics: age, gender, country. Clinical: ESA doses, iron therapy, dialysis sessions per week, time since hospitalization, transfusion and dialysis. Lab: Hb, Hb delta, MCV, TSAT**, ferritin, CRP, Na, P, Ca, ALB. Others: **Hospitalization & Transfusion Events.**	LSTM, Phased LSTM, GRU, GRU-D, ARX-3	Hold-out<br>Training: 71%<br>Conventional Test: 10%<br>Prospective test: 9%<br>Validation: 10%	**GRU performed best (MAE 0.676, RI 20.32%).**
Petrović *et al*. (2020)[119]	Diagnostic support of SCD	2695 labeled RBCs<br><br>(700 sickle-shaped RBCs)	121 features per cell, including 41 **shapes**, 18 color, and 62 **texture** variables.	SVM, DT, RF, ET, GB, KNN, MLP	Hold-out<br>Training: 70%<br>Testing: 30%<br><br>10-fold CV	GB and RF were the best-performing models, achieving F-measures of 93.5% and 93.36% and SDS-scores of 95.18% and 95.05%, respectively.
Pullakhandam *et al*. (2024)[120]	Detect IDA using CBC data	19,975 individuals<br><br>(972 with IDA)	**Demographics: age, sex, pregnancy status. Lab: Hb, HCT, RDW, MCV, MCH, LYM, MO.**	GB, LR, RF, KNN, NB, XGB	Hold-out<br>Training: 80%<br>Testing: 20%<br><br>RFE with CV<br><br>EV (n=502; used as a test set)	**GB performed best (AUC 0.87).**
Qadah *et al*. (2024)[121]	Improve anemia diagnosis using CBC data	364 patients<br><br>(not explicitly stated)	Demographics: sex. Lab: **RBC**, **Hb**, **HCT**, **MCV**, MCH, **RDW**, WBC, PLT.	LR, RF, XGB, SVM, NN, KNN, DT, NB, AdaBoost	Hold-out<br>Training: 80%<br>Testing: 20%	XGB achieved the best AUC (0.945); XGB and RF had the highest accuracy (0.863), recall (0.96), and precision (0.89).
Ranganathan *et al*. (2006)[122]	Estimate Hb levels using color analysis	2007 blood samples<br><br>(NA)	Color-coded values of blood samples (RGB values) as input, and Hb values obtained using the cyanmethemoglobin method as the desired output.	ANN	Not explicitly stated	ANN accurately estimated Hb levels, confirming a strong link between blood color and Hb concentration.

Rizzuto *et al*. (2021)[123]	**Discriminate healthy from hemolytic anemia patients**	32 participants<br><br>(21 with hemolytic anemia)	Image features (extracted from time-lapse microscopy videos of RBCs).	DL (AlexNet) + SVM	Leave-One-Out CV	The model correctly classified healthy individuals versus RHHA with 91% efficiency, and among RHHA subtypes with 82% efficiency.
Rustam *et al*. (2022)[124]	Predict **β-thalassemia carriers** using **CBC**	5,066 individuals<br><br>(2,051 **β-thalassemia trait)**	Demographics: age, sex. Lab: RBC, Hb, HCT, MCV, MCH, MCHC, RDW, PLT, WBC.	DT, GBM, AdaBoost, SVM, RF, ETC, LR, LSTM, GRU, CNN, CNN-LSTM	Hold-out<br>Training: 90%<br>Testing: 10%	RF and ETC achieved the highest accuracy (96%).
Salma *et al*. (2025)[125]	Predict childhood anemia in Bangladesh	2,278 children<br><br>(not explicitly stated)	Child: **age**, sex, **breastfeeding status**, iron supplementation, VitA supplementation, parasite treatment. Parental: **age** and **education**. Household and environmental: **children under 5**, **toilet facility**, **source of drinking water**.	GB, RF, XGB, SVM, NB, KNN, Bagging	Hold-out<br>Training: 70%<br>Testing: 30%<br><br>10-fold CV	GB performed best (accuracy 87.46%, AUC 0.91, F1 score 0.90, precision 95.35%).
Sani *et al*. (2024)[35]	Diagnose SCD	621 images<br><br>(Hb SS: 249; Hb AS: 262)	Image-based features from mIEF digital scans.	ResNet34	Hold-out<br>Training: 70%<br>Testing: 10%<br>Validation: 20%	ResNet34 achieved 90.1% test accuracy, with 0.912 precision, 0.897 recall, and 0.904 F1 score, matching HPLC performance while improving Hb variant specificity at a fraction of the cost.
Saputra *et al*. (2023)[126]	Predict and classify different types of anemia	190 patients<br><br>(IDA: 104; HbE: 41; **β-thalassemia trait**: 24;	Lab: RBC, **Hb**, **HCT**, **MCV**, **MCH**, MCHC, **RDW**.	ELM, RF, KNN, SVM	Hold-out<br>Training: 67%<br>Testing: 33%	ELM outperformed all models with 99.2% accuracy, 99.3% precision, 98.4% sensitivity, and 98.8% F1-score in

		combination: 21)				classifying four anemia types.
Saputra *et al*. (2024)[127]	Improve anemia diagnosis	128 samples<br><br>(IDA: 104; **β-thalassemia carriers: 24)**	Lab: **Hb**, **HCT**, **RBC**, **MCV**, MCH, MCHC, **RDW**.	RF, NB, SVM, applied with SMOTE-MRS	10-fold CV	SMOTE-MRS achieved 97.3% accuracy, 99% recall, 96.8% precision, 97.9% F1 score, and 99.4% AUC.
Sarsam *et al*. (2022)[128]	Early anemia recognition	1,738,759 tweets<br><br>(not explicitly stated)	**T**weet content, **anemia-related symptoms** and causes, emotions (**fear**, **sadness**, anger, joy), sentiment intensity scores, thematic topics, emotion-symptom associations.	SMO, RF, Bagging, OneR	10-fold CV	SMO achieved 98.96% accuracy in identifying anemia-related content when emotional features were included.
Schipper *et al*. (2024)[29]	Identify hemoglobinopathies and distinguish them from IDA	5,908 patients<br><br>(not explicitly stated)	**Lab: Hb, MCV, MCH, MCHC, RBC, RDW, PLT, Retic.**	LR, XGB	Hold-out<br>Training:84%<br>Testing: 16%<br><br>5-fold CV<br><br>EV (n=2,656)	XGB outperformed LR across all categories: AUCs: 0.91 (α-thalassemia), 0.89 (β-thalassemia), 0.88 (structural variants), and 0.97 in differentiating thalassemia from IDA.
Sehar *et al*. (2025)[17]	Detect anemia using eye conjunctiva images	764 images<br><br>(439 with anemia)	**Image-derived features** including **color intensity, CIELAB channel means**, and **segmented conjunctival regions.**	SVM, KNN, NB, DT, Regression model, GoogLeNet, Voting Ensemble, Stacking Ensemble	Hold-out<br>Training: 70%<br>Testing: 20%<br>Validation: 10%<br><br>10-fold CV	**Stacking Ensemble** achieved the best performance (**AUC = 0.97** and **F1-score = 91%).**
Su *et al*. (2024)[129]	Predict post-chemotherapy anemia in osteosarcoma	410 patients<br><br>(194 with anemia)	**Demographics: age, gender, marital status, nationality, height, weight, BMI. Clinical: BP, smoking, alcohol, tumor-related (surgery type and**	LR, MLR, RF, SVM, LASSO, intersection model (composite model)	Hold-out<br>Training: 70%<br>Testing: 30%<br><br>10-fold CV	SVM had highest AUC in training (0.903), but the **intersection model had the most relevant performance with** AUC

			**status, primary tumor site, TNM stage, Ki-67 expression), treatment details (chemotherapy, radiotherapy, targeted therapy, immunotherapy). Lab: WBC, RBC, Hb, PLT, LYM, MO, NEU, AST, ALT, ALP, LDH, GGT, creatinine, BUN, uric acid, CrCl, TP, ALB, TRF, K, Na, Cl, Ca, PT, APTT, TT, FIB, DD, CRP, ESR, Procalcitonin, C3, C4, IgG, IgA, IgM, lymphocyte subtypes, CEA, AFP, CA-125, CA-153, CA-199, SCC-Ag, Cyfra21-1, TSH, T3, T4, FT3, FT4, TG, total cholesterol, HDL, LDL.**			= **0.802** on the validation set.
Terzi *et al*. (2022)[130]	Diagnose IDA	516 patients<br><br>(359 IDA-positive)	Demographics: age, gender. Lab: **Hb**, HCT, MCV, MCHC, RBC, RDW, **Fe**, **ferritin**, UIBC.	XGB+Boruta	CV performed (folds' number not specified)	XGB+Boruta achieved outstanding performance (AUC 0.999, ACC 0.990, MCC 0.968).
Tesfaye *et al*. (2024)[131]	Predict childhood anemia	7,795 children<br><br>(4,691 with anemia)	Socio-demographic and household variables: place of residence, **maternal age**, **maternal education**, **maternal anemia**, **employment status**, **wealth index**, source of drinking water. Child-health variables: **age**, sex, **morbidity**, **stunting**, **wasting**, deworming treatment.	LR, RF, DT, KNN	Hold-out<br>Training: 80%<br>Testing: 20%	LR performed best (AUC 0.69, accuracy 66%, sensitivity 82%, specificity 42%).

Uçucu et al. (2024)[132]	Distinguish β-thalassemia minor from IDA	396 patients<br><br>(216 IDA; 180 β-thalassemia minor)	Demographics: age, sex. Lab: **RBC**, Hb, HCT, **MCV**, MCH, **RDW**, Fe, TIBC, ferritin, HbA2, HbA.	ANN, DT	10-fold CV	ANN performed best (AUC 0.995, sensitivity 99.5%, specificity 99.4%, F1 score 0.995), outperforming traditional indices.
Valles-Coral *et al*. (2024)[23]	Detect IDA through finger-tip video images	909 participants<br><br>(287 anemic)	Fingertip video-derived features (**mean red channel intensity** and its **temporal variation**). Biometric data (age, sex, height, weight, **BMI**, **HR**, abdominal circumference, **SpO_2**, Hb). Symptoms (**fatigue**, **weakness**, palpitations, shortness of breath, **dizziness**, chest pain, **cold extremities**, headache).	CNN (ResNet18)	Hold-out<br>Training: 70%<br>Testing: 10%<br>Validation: 20%	CNN achieved high performance: accuracy 98.4%, precision 98.3%, sensitivity 98.4%, F1 score 98.35%, AUC 0.988.
Vohra *et al*. (2022)[133]	Stratifying anemia severity	364 patients<br><br>(256 mild, 92 moderate, 16 severe anemia)	Demographics: **age**, sex. Lab: **Hb**, MCV, MCH, **MCHC**, RBC, WBC, **PLT**, **PCV**, RDW.	MLP, LR, DT, RF, SVM, NB	Hold-out<br>Training: 80%<br>Testing: 20%<br><br>10-fold CV	MLP achieved the best overall performance with 94.21% accuracy and AUC up to 0.992.
Wang *et al*. (2025)[30]	Identify and differentiate thalassemia and IDA	1,819 participants<br><br>(IDA: 460; Thalassemia: 801)	Demographics: age, sex, pregnancy status. Lab: RBC, **Hb**, HCT, **MCV**, **MCH**, MCHC, **RDW-SD**, RDW-CV, PLT, MPV, PDW.	XGB, RF, SVC, NB, LR, KNN	Hold-out<br>Training: 70%<br>Testing: 30%<br><br>5-fold CV<br><br>EV (n=378)	XGB performed best (AUC 0.956, F1-score 93.64%).
Yagmur *et al*. (2024)[134]	Classify five different types of anemia	15,300 patients<br><br>(all anemia types: 5,553)	Demographics: gender. LAB: WBC, NEU, LYM, MO, EOS, BASO, RBC, **Hb**, **HCT**, **MCV**, **MCH**, MCHC, **RDW**, PLT,	TreeBagger, TreeBagger + CSO, CSA, JAYA (with/without SMOTE)	10-fold CV	TreeBagger+CSA+SMOTE achieved the highest accuracy (99.92%).

			MPV, **PCT**, PDW, **iron**, TSAT, **ferritin**, **folate**, **VitB12**.			
Yilmaz *et al*. (2012)[135]	Diagnose IDA using standard lab values	2,600 blood samples<br><br>(122 with IDA)	Lab: RBC, Hb, HCT, MCV, MCH, MCHC.	FFN, CFN, DDN, TDN, LVQ, PNN	Hold-out<br>Training: 77%<br>Testing: 23%	DDN achieved 97.6% sensitivity and 99.16% accuracy.
Zemariam *et al*. (2024)[136]	Predict anemia in youth girls	5,642 blood samples<br><br>(1,435 anemic)	Demographics: **age**, **region**, **religion**, **marital status**, **educational status**, occupation, **place of residence**, sex of the household head. Household and socioeconomic: **wealth index**, **family size**, **media exposure**, **toilet facility**, **source of drinking and water**, **altitude** of residence. Clinical: smoking, pregnancy, **contraceptive use**.	RF, XGB, LGBM, DT, SVM, KNN, NB, LR (all with and w/o SMOTE)	Hold-out<br>Training: 80%<br>Testing: 20%<br><br>10-fold CV	RF-SMOTE performed best (AUC 0.82, accuracy 74%).
Zhang *et al*. (2022)[137]	Predict anemia in emergency department using facial images	316 patients (6,993 images)<br><br>(217 anemic)	Facial image data only, captured in a controlled environment using a tablet; top predictors: **image features from the eyes and lips**.	InceptionV3, ResNet50, DenseNet121, EfficientNetB0, MobileNet	Hold-out<br>Training: 70%<br>Testing: 30%<br><br>5-fold CV	InceptionV3 performed best (accuracy 84.02%, sensitivity 92.59%, specificity 69.23%), outperforming experienced emergency physicians in anemia detection.
Zhang *et al*. (2023)[138]	Distinguish thalassemia trait from IDA	798 patients<br><br>(248 with thalassemia trait)	Clinical: **age**, sex, **pregnancy status**. Lab: **RBC**, **RDW-SD**, RDW-CV, **Hb**, **MCV**, **MCHC**.	RF, LinearSVC, MLP, LR	5-fold CV<br><br>EV (n=236)	RF achieved AUC of 0.942, accuracy 91.53%, sensitivity 91.91%, and specificity 91.00%, outperforming all other models in EV

Zhang *et al*. (2025)[139]	Screen for anemia using facial spectral reflectance and ML	156 patients<br><br>(78 with anemia)	Spectral reflectance values at 8 facial sites (glabellum, forehead, nose, jaw, right zygomatic, left zygomatic, right cheek, left cheek) across 400–700 nm wavelengths (**glabellum 750nm**, **right cheek 520nm**, **right zygomatic 570nm**, **jaw 570nm**, **left cheek 610nm**).	LR, DT, SVM, RF, KNN, ANN, NB, XGB, AdaBoost, LGBM	Hold-out<br>Training: 70%<br>Testing: 30%<br><br>10-fold CV	SVM achieved highest accuracy (0.875), AUC (0.974), and average precision (0.964).
**Anemia treatment**						
Barbieri *et al*. (2015)[140]	Predict long-term Hb response to ESA/iron therapy in HD	4,135 patients<br><br>(NA)	Demographics: height. Clinical: dry body weight, pre-dialysis weight, **OcmKt/V**, therapy (**darbepoetin**, **iron**). Lab: **Hb**, **ferritin**, **ALB**, **WBC**.	MLP	Hold-out<br>Training: 66%<br>Testing: 17%<br>Validation: 17%	**ANN** provided **clinically accurate predictions** of Hb levels with a **MAE around 0.57 g/dL, minimal bias,** and **90–93% of predictions within ±1 g/d.**
Barbieri *et al*. (2016)[141]	Optimize anemia management in HD	752 patients<br><br>(NA)	Demographics: sex, height. Clinical: dry weight, predialysis weight, Kt/V, medications (**ESA**, iron), **change in Hb** over the past month. Lab: **ferritin**, **TSAT**, **WBC**, **CRP**, **MCV**, **MCH**, **ALB**, Ca, P, Na, K.	ANN	ANN was trained on **170,000 past records** and validated on **>40,000,** entirely separate from the study sample.	ANN within the ACM improved on-target Hb (from 70.6% to 76.6%, up to 83.2% when followed), reduced Hb variability (SD from 0.95 to 0.83 g/dL), and decreased ESA use by 25%.
Barbieri *et al*. (2016)[142]	Predict long-term Hb response to darbopoietin and iron in HD	1,558 patients<br><br>(NA)	Demographics: age, gender, height. Clinical: pre- and post-dialysis weight, duration on dialysis, comorbidities (DM, IHD, HF, PAD, stroke, chronic respiratory disease), dialysis parameters (treatment modality, vascular access type,	ANN	Hold-out<br>Training: 60%<br>Testing: 40%	ANN predicted Hb at 3 months with a mean absolute error of ~0.75 g/dL; 89% of predictions were within ±1.5 g/dL.

			duration, sessions per week, **eKt/V**, spKt/V), anemia therapy (**darbepoetin**, **iron**). Lab: Hb, **ferritin**, **TSAT**, ALB, P, **CRP**.			
Brier *et al.* (2011)[143]	Improve anemia management in HD	60 patients<br><br>(NA)	ESA doses, Hb levels over time, iron status indicators, biomarkers (oncostatin M receptor β, cysteine/histidine-rich 1).	ANN, LR	Not explicitly stated	**ANN** outperformed LR in predicting Hb response to ESA dosing.
Bucalo *et al.* (2018)[144]	Improve anemia management in HD patients	650 patients<br><br>(431 were managed with the ANN-based model)	Demographics: sex, height. Clinical: **dry weight**, dialysis modality and frequency, **ESA dose**, iron dose, vascular access type. Lab: **Hb**, **Ferritin**, **TSAT**, MCV, MCH, CRP, **ALB**.	ANN	No	ANN improved Hb control, reduced ESA use and variability, and lowered adverse events.
Gabutti *et al.* (2006)[145]	Predict EPO dose in HD	432 patients<br><br>(NA)	**Demographics: age, sex, weight. Clinical: DM, cardiomyopathy, ACE inhibitor, EPO dose, EPO administration route, iron dose,** Kt/V. **Lab: Hb, creatinine, BUN, pH,** P, Ca, ALB, **ferritin**, PTH, CRP.	ANN, LinearR	Hold-out (two-step data splitting process)<br>1st split:<br>25%: validation<br>75%: model development<br><br>2nd split (within 75%):<br>Training: 50%<br>Testing: 40%<br>CV: 10%	**ANN significantly outperformed LinearR and nephrologists**, achieving higher sensitivity in predicting EPO dose (78% vs. 44%) and better detection of underdosing (48% vs. 25%) with greater specificity (92% vs. 83%).
Gaweda *et al.* (2005)[146]	Personalized EPO dosing in anemia management	186 patients<br><br>(NA)	**EPO dose, Hb, TSAT.**	Hybrid model (Q-learning +RBF+Fuzzy)	Monte Carlo CV	Hybrid model individualized EPO dosing effectively, maintaining Hb within 11–12 g/dL with lower variability and

						comparable or better performance than standard protocols.
Gaweda *et al*. (2008)[147]	Predict Hb response to EPO in HD	186 patients<br><br>(NA)	**EPO dose, Hb, TSAT.**	LinearR (applied to Fuzzy classification, Hard classification and Population-based)	Monte Carlo CV	**Fuzzy** achieved the lowest prediction error (MSE **= 1.20 ± 0.07).**
Gaweda *et al*. (2008)[148]	Predict and control EPO administration in the anemia of HD	186 patients<br><br>(NA)	**EPO dose, Hb, Hb levels from the 2 previous months.**	ANN	Hold-out<br>Training: 70%<br>Testing: 30%	ANN maintained Hb at 11.6 ± 0.4 g/dL, reduced variability (0.60 vs. 0.75 g/dL), and improved target control (P = 0.02) without increasing EPO dose.
Guerrero *et al*. (2003)[149]	Personalizing EPO dosing in HD and predicting Hb levels	110 patients<br><br>(NA)	Demographics: age, weight. Treatment: **iron dose**, **EPO dose**, **EPO administrations per week**, EPO isoform, Lab: **Hb**, **ferritin**.	MLP, Elman Recurrent NN, FIR Network, Ensemble models	Hold-out<br>Training: 70%<br>Testing: 30%<br><br>CV performed (folds' number not specified)	MLP achieved the best performance with MAE ≈ 0.15 g/dL and over 97% of predictions within ±0.5 g/dL.
Kang *et al*. (2024)[150]	Optimize anemia management in HD	12,993 samples<br><br>(NA)	Demographics: **age**, **sex**. Clinical: dialysis duration, dialysis metrics (SBP, DBP, HR, **dry weight**, **total ultrafiltration volume**), RBC **transfusion events**, medications: **oral or IV iron**, **ESA**. Lab: **Hb**, **ALB**, **WBC**, PLT, BUN, creatinine, Na, K, Cl, uric acid, Ca, P, PTH, glucose, TP, AST, ALT, ALP, total and HDL-cholesterol, triglycerides, **iron**, TIBC,	LinearR, XGB, MLP, LSTM, GRU, GRU-GNL, GRU-AM	Hold-out<br>Training: 70%<br>Testing: 20%<br>Validation: 10%	GRU-AM achieved R^2 = 0.60 for Hb, 0.78 accuracy in ESA dosing, and 0.99 in transfusion alerts, closely aligning with expert decisions.

			ferritin, HBsAg, HbsAb, HCVAb.			
Martín-Guerrero *et al*. (2003)[151]	Predict Hb response and individualize EPO dosing in HD	110 patients<br><br>(NA)	Demographics: **age**, **weight**. Clinical: EPO dose, EPO administrations/month, **EPO isoform**, iron dose. Lab: **Hb**, HCT, ferritin.	PD-SVR, SVR, MLP	Not explicitly stated	PD-SVR performed best (RMSE = 0.48 g/dL, r = 0.89, and <0.25 g/dL prediction error in 83.33% of cases).
Yun *et al*. (2021)[152]	Predict Hb and recommend ESA doses in HD to optimize anemia management	466 patients (12,907 dialysis sessions)<br><br>(NA)	Demographics: age, sex. Clinical: dry weight, pre/post-dialysis weight, urea reduction rate, **ESA dose**. Lab: **Hb**, MCV, MCH, MCHC, RDW, **PLT**, ALB, **iron**, urea reduction ratio.	GRU-RNN, XGB, MLP, LR	Hold-out<br>Training: 80%<br>Testing: 10%<br>Validation: 10%	GRU-RNN achieved high Hb prediction accuracy (MAE = 0.59 g/dL) and significantly improved ESA dose precision, outperforming real-world physician dosing.
Zhang *et al*. (2023)[153]	Predict Hb response to EPO treatment in renal anemia	291 patients<br><br>(NA)	Clinical: **EPO dose**. Lab: **Hb**, **RBC production rate**, Hb/RBC, **time-delay dynamics of erythropoiesis**, EPO elimination rate, RBC lifespan, HCT, Clearance and conversion rates.	PINN, ARX, NARX	Not explicitly stated	PINN performed the best (lowest RMSE).

Table S2. Minimization of Blood Loss and Bleeding.

Reference (Year)	PBM Application	Sample Size (case group)	Model Variables Top Predictors (bold)	Models	Model Validation	Key Findings
**Cardiology**						
Alsayegh *et al*. (2022)[154]	Predict in-hospital bleeding in ACS patients and assess the impact of admission anemia	4,044 patients<br><br>(119 with bleeding)	Demographics: **age**, sex, country. Clinical: smoking, alcohol use, comorbidities (SAH, DM, dyslipidemia, **CKD**, **CVD**, TIA, cancer), in-hospital events (**HF**, infarction, bleeding, stroke, PCI). Lab: **Hb**.	RF, GBM, SVM, LR	Hold-out<br>Training: 80%<br>Testing: 20%<br><br>10-fold CV	RF predicted in-hospital bleeding best (AUC of 0.76, 70.6% accuracy, 57.8% sensitivity, and 72.1% specificity).
D'Ascenzo *et al*. (2021)[155]	Predict adverse events (including major bleeding) following an acute coronary syndrome	19,826 patients<br><br>(562 experienced major bleeding)	Demographics: **age**, sex. Clinical: DM, SAH, hyperlipidemia, **PAD**, MI, PCI, CABG, stroke, **previous bleeding**, **malignancy**, STEMI presentation, **LVEF**, multivessel disease, complete revascularization, medications (Beta blockers, ACE/ARBs, statins, **anticoagulation**, PPI). Procedure: vascular access site, PCI with drug-eluting stent. Lab: **eGFR**, **Hb**.	AdaBoost, NB, RF, KNN	Hold-out<br>Training: 80%<br>Testing: 20%<br><br>EV (n=3444)	AdaBoost performed best, achieving an AUC of 0.86 in the EV for predicting major bleeding.
Hamilton *et al*. (2023)[37]	Predict complications after PCI (including bleeding and transfusion)	107,793 procedures<br><br>(2,592 transfused; 942 experienced bleeding)	**Demographics: age, sex, race, height, weight. Clinical:** DM, HF, prior PCI, prior CABG, CVD, PAD, lung disease, smoking, **frailty**, **LVEF**, clinical status, cardiovascular instability, ventricular support, cardiac arrest, stress test results, PCI indication, Cath	XGB, LR	Hold-out<br>Training: 60%<br>Testing: 40%<br><br>3-fold CV<br><br>EV (n=56,583)	XGB had excellent discrimination: AUC 0.917 (transfusion) and 0.887 (bleeding); outperforming LR for all outcomes in internal and EV.

			lab indication. Lab: **Hb**, total cholesterol, HDL, **creatinine**.			
Kou *et al*. (2025)[156]	Predict GI bleeding in acute MI	3,656 patients<br><br>(1,506 with GI bleeding)	**Demographics: age, sex, occupation, marital status. Clinical: CHD, SAH, DM, smoking, alcohol. Others: hospital LOS. Lab: RBC, Hb, HCT, myoglobin, NT-proBNP, Tbil, FIB, AG, ALB, ALB/GLO, ALP, AST, ApoB, ApoA1, APTT, APTTR, PT, TT, INR, WBC, NEU, BASO, EOS, MO, Na, Ca, ChE, Cl, P, creatinine, CysC, Dbil, Ibil, glucose, GGT, GLO, HDL, LDL, total cholesterol, HCY, MCH, MCHC, MCV, PLT, RDW, triglycerides, TBA, uric acid.**	LR, KNN, SVM, DT, RF, XGB, NN	Hold-out<br>Training: 80%<br>Testing: 20%<br><br>10-fold CV<br><br>EV (n=1,746)	**RF performed best (AUC 0.77 internal, and AUC 0.75 external).**
Mortazavi *et al*. (2019)[36]	Predict major bleeding after PCI	3,316,465 procedures<br><br>(149,724 experienced major bleeding)	**Demographics: age**, **sex**, BMI. Clinical: DM, SAH, **CKD**, PAD, lung disease, **NYHA**, LVEF, previous PCI, previous CABG. Procedure: **STEMI status**, PCI lesion characteristics, **shock**, **urgency of procedure**. Lab: **Hb**, **eGFR**, creatinine.	XGB, LASSO-regularized LR	5-fold CV	XGB outperformed traditional NCDR models, achieving a C-statistic of 0.82 (vs. 0.78 for the full NCDR model), and identified 3.7% more high-risk bleeding cases.
Niimi *et al*. (2022)[157]	Predict adverse events (including bleeding) after PCI	22,958 patients<br><br>(16,484 with bleeding)	Demographics: age, **sex**, **BMI**. Clinical: DM, HF, PAD, COPD, MI, **PCI urgency**, **STEMI**/NSTEMI presence, cardiogenic shock, use of intra-aortic balloon pump, **anticoagulant use**. Lab: **Hb**, creatinine, **eGFR**.	LR, XGB	Hold-out<br>Training: 75%<br>Testing: 25%<br><br>3-fold CV	XGB modestly improve prediction for bleeding compared to LR (AUC 0.79 vs. 0.75).
Rayfield *et al.* (2020)[38]	Predict bleeding after PCI	15,603 patients<br><br>(281 with bleeding)	Demographics: age, gender, BMI. Clinical: DM, CKD, PCI access site, shock status, cardiac arrest within 24 hours, **ACS**, **PCI timing and**	BCT (used to develop AI-BR)	Hold-out<br>Training: 75%<br>Testing: 25%	AI-BR outperformed the ACC-BR model in predicting post-PCI

			**indication**, **ischemic changes on EKG**, **culprit lesion treatment**, **number of native vessels treated**. Lab: Hb, creatinine, eGFR.		10-fold CV	bleeding risk (AUC 0.873 vs. AUC 0.764).
**Cardiothoracic**						
Abbasi *et al*. (2023)[43]	Predict major postoperative complications, including bleeding, in cardiac surgery	662,772 patients<br><br>(19,109 experienced bleeding)	Demographics: age, gender, race, BMI. Clinical: SAH, DM, CAD, MI, medication use (antiplatelets, anticoagulants). Procedure: CPB time, aortic cross-clamp time, circulatory arrest duration. Postoperative: **mechanical ventilation duration**, **transfusions**, **new-onset arrhythmias**, **anoxic brain injury**, **ICU length of stay**.	NN, PFI, LR	Hold-out<br>Training: 80%<br>Testing: 10%<br>Validation: 10%	NN achieved high predictive performance for hemorrhage (AUC 0.97).
Ahmadzadeh *et al*. (2025)[158]	Predict IVH in very low birth weight infants	387 infants<br><br>(189 with IVH)	Demographics: maternal age, **sex of the infant**. Clinical: mode of delivery, endotracheal intubation in the delivery room**, birth weight**, **gestational age at delivery**, **APGAR**.	DNN-A, SVM, DT, LR, NB, XGB	Hold-out<br>Training: 70%<br>Testing: 30%<br><br>5-fold CV	DNN-A achieved highest performance (AUC 0.87, accuracy 0.871).
Dong *et al*. (2025)[159]	Predict GI bleeding after CABG	13,399 patients<br><br>(803 with GI bleeding)	Demographics: **age**, sex, BMI. Clinical: GI bleeding history, PCI, **LVEF**, SAH, AF, DM, coagulation disorders, **HF**, **anemia**, **CKD**, CVD, PVD, VHD, GI ulcer, gastritis, hyperlipidemia. Lab: **Hb**, HCT, RBC, **PLT**, **WBC**, AST, ALT, Tbil, **ALB**, **LDH**, creatinine, BUN, **INR**, PT, APTT.	XGB, RF, LR, NB, MLP, SVM	5-fold CV<br><br>EV (n=3041)	XGB performed best (AUCs from 0.781 to 0.851 across three cohorts), and outperformed traditional bleeding risk scores.
Gao *et al*. (2022)[160]	Predict major bleeding after CABG	1,045 patients<br><br>(74 experienced major bleeding)	**Demographics: age, gender, height, weight, BSA. Clinical: smoking, angina, MI, arrythmia, previous surgery, DM, hyperlipidemia, SAH,**	CIRF, SGBT, XGB, RF, LR	Hold-out<br>Training: 70%<br>Testing: 30%	**CIRF had the highest predictive accuracy (AUC 0.831), outperforming TRUST**

			**kidney failure, dialysis, chronic pulmonary disease, CHF, anemia, PVD, venous disease, CVD, PTCA, CAD, previous thrombolysis, CHD family history, medications (statin, anticoagulant, antiplatelet), TRUST and WILL-BLEED scores. Surgical procedure: operation time, blood loss, intraoperative transfusion, CPB, urine output, Hb decrease. Lab: RBC, WBC, PLT, PDW, MPV, PLT-large cell ration, thrombocytocrit, Hb, TP, ALB, K, Na, Ca, glucose, BUN, creatinine, eGFR, HSCRP, NT-proBNP, PT, INR, postoperative creatine and NT-proBNP. Others: LOS.**		5-fold CV	**(AUC 0.629) and WILL-BLEED (AUC 0.557) scores.**
Harm *et al.* (2024)[161]	Predict major bleeding in CAD	595 patients<br><br>(16 experienced bleeding)	**Demographics: age, sex, BMI. Clinical: chronic coronary syndrome, unstable angina, STEMI, NSTEMI, SAH, hyperlipidemia, DM, smoking, obesity, AF, previous CABG, MI, LVEF, medications (statins, ezetimibe, AAS, clopidogrel, ticagrelor, prasugrel, cangrelor, anticoagulants, ACE inhibitor, aldosterone agonists, ARBs,** β-blockers, diuretics). **Lab: total cholesterol, LDL, HDL, triglycerides, HBA1c, renal function, PLT, PLT aggregation, PLT lipidomics.**	LASSO	10-fold CV	**LASSO** achieved high predictive accuracy for **bleeding events (AUC 0.804).**
Hu *et al.* (2024)[162]	Predict high bleeding risk in valve replacement patients on warfarin	2,970 patients<br><br>(131 with high bleeding risk - INR ≥ 4.5)	Demographics: age, sex, height, weight, **BSA**. Clinical: AF, CHD, SAH, DM, CVD, NYHA, previous bleeding, medications (amiodarone, aspirin, triazole antifungals, acarbose, levothyroxine, antibiotics). Lab: ALT, AST, GGT, LDH, **creatinine**, ALB, Hb, **PLT**, **WBC**, T4, T3, **TSH**, genetic variables (CYP2C9 and VKORC1-1639 gene variants).	LR, SVM, RF, XGB, DNN	Hold-out<br>Training: 80%<br>Testing: 20%<br><br>10-fold CV<br><br>EV (n=594)	XGB performed best (AUC 0.882 internally and 0.853 externally), outperforming traditional bleeding scores (ORBIT, ATRIA).

Huang *et al*. (2015)[163]	Predict postoperative bleeding in CPB	52 patients<br><br>(Not explicitly stated)	**Lab: PLT, thromboelastograph-reaction time, DD, PT, thromboelastograph-coagulation index**.	ANN	Hold-out<br>Training: 75%<br>Testing: 25%	ANN stratified CPB patients into bleeding risk categories with 69.2% exact match and 92.3% accuracy within one grading difference.
Hui *et al*. (2023)[164]	Predict bleeding after cardiac surgery	2,000 patients<br><br>(343 experienced moderate to massive bleeding)	Demographics: age, BMI. Clinical: smoking, DM, SAH, dyslipidemia, dialysis, renal transplant, CVD, PVD, lung disease, CHF, MI, NYHA, CCS angina classification, endocarditis, medications (immunosuppression, anticoagulation antiplatelet use - aspirin, clopidogrel, ticagrelor, tirofiban, eptifibatide), previous cardiac interventions, LVEF. Intraoperative: surgeon, surgery type, **CPB duration**, cross-clamp time, temperature management, cardiogenic shock, transfusions, fluid administration, **blood gas parameters**. Postoperative/ICU: MAP, **noradrenaline dose**, **Hb**, lactate, pH, FIB, INR, lactate, creatinine, Ca, **ALB**.	EVC, LR, RF, XGB, DNN	Hold-out<br>Training: 80%<br>Testing: 20%<br><br>5-fold CV	EVC outperformed all models (AUC 0.797, AUPRC 0.452) for Dyke-defined bleeding, surpassing the Papworth Bleeding Risk Score (AUC 0.471).
Kim *et al*. (2021)[165]	Predict bleeding in mechanical valve replacement	598 patients<br><br>(143 experienced major bleeding)	**Demographics: age, sex, BMI. Clinical: medical history (GI bleeding, MI, major bleeding, CAD, SAH, HF, DM, cancer, hepatic disease**, pulmonary embolism, AF, **stroke**), medications (antibiotics, acetaminophen, amiodarone, antihistamines, **antiplatelet agents**, digoxin, herbs, NSAIDs, statins, **steroids**). **Surgery procedure: surgery type, maze operation, operation time.** Lab: **Hb**, PLT, **creatinine**, **labile INR**, INR on discharge.	RF	Out-of-bag	RF achieved **an AUC of 0.87**, with **80.77% sensitivity, 87.67% specificity, and 85.86% accuracy.**
Meyer *et al*. (2018)[166]	Predict postoperative complications in ICU, including bleeding	11,492 ICU admissions<br><br>(2,322 experienced bleeding)	**Demographics: age, sex, height, weight. Clinical: ASA, SBP, DBP, pulmonary artery pressure, central venous pressure, ventilator FiO_2, HR, RR, temperature, bleeding and urine flow rate. Surgical procedure: surgery type, duration, and urgency, anesthesia type, cardioplegic solution used, aortic cross-calm time, CPB, anesthetic monitoring time. Lab: bicarbonate, glucose, Hb,**	GRU-RNN	Hold-out<br>Training: 90%<br>Testing: 10%<br><br>10-fold CV<br><br>EV (n=5,898)	GRU-RNN achieved AUC = 0.87 for bleeding (vs 0.58 for the clinical reference), with sensitivity = 0.74 and specificity = 0.86.

			SpO_2, **$PaCO_2$, PaO_2, pH, K, Na, ALB, bil, urea, CRP, CK, GGT, AST, Hb, HCT, INR, creatinine, WBC, LDH, Mg, APTT, PT, PLT.**			
Perduca *et al*. (2024)[167]	Predict hemostasis after EC for cardiac surgery	598 patients (NA)	Demographics: age, **sex**, **weight**, BMI. Clinical: DM, renal insufficiency, antiplatelet agents. Surgical Procedure: **EC duration**, coronary artery bypass, **cardioplegia type**, circuit type. Lab: Hb, **PLT**, **PT**, **FIB**, creatinine.	ENPLR, GAM, RF, XGB, SVM, SL, GLMNet	Hold-out Training: 80% Testing: 20% 10-fold CV	SL excelled in predicting PT ratio (RMSE: 8.66%) and FIB assay (RMSE: 0.44 g/L), while ENPLR performed best for PLT predictions (RMSE: 38.27×10^9/L).
Yang *et al*. (2025)[168]	Predict bleeding after CABG with dual antiplatelet therapy	561 patients (165 with bleeding)	**Demographics: age, BMI. Clinical: DM, history of bleeding, CKD, anemia, cancer, history of surgery, preoperative eGFR. Postoperative: DAPT-Ticagrelor.**	XGB, SVC, MLP, RF, LR	Hold-out Training: 70% Testing: 30% 5-fold CV EV (n=100)	XGB outperformed all other models and PRECISE-DAPT (AUC 0.915 (internal), 0.926 (external), AUC-PR (0.703 external), F1 score: 0.727.
Zhao *et al*. (2023)[169]	Predict bleeding in MI after PCI	16,736 patients (70 experienced bleeding)	Demographics: **age**, sex, BMI, education, medical insurance, marital status. Clinical: **HR**, **SBP**, **DBP**, Killip class, chest pain, SAH, hyperlipidemia, DM, MI, angina, HF, bleeding history, CKD, dialysis, PAD, aortic disease, cancer, rheumatic/immune diseases, medication use (NSAIDs, beta-blockers, immunosuppressives, Ca channel blockers, ACE inhibitors, statins, diuretics, aldosterone antagonists, traditional Chinese medicine, bivalirudin), smoking, alcohol, diet, exercise, EKG and angiographic findings (ST-segment changes, TIMI flow, triple-vessel	XGB	Hold-out Training: 50% Testing: 50% 10-fold CV	XGB outperformed CRUSADE and ACUITY-HORIZONS (AUC 0.837 vs. 0.741 and 0.731).

			disease), previous CABG or PCI or revascularization, presentation characteristics. Lab: **Hb**, **HCT**, PLT, WBC, NEU, **ClCr**, **NT-proBNP**, **CK-MB**, troponins, glucose, cholesterol, LDL, HDL, triglycerides, **K**, Na, Cl, **Tbil**, **Dbil**, HbA1C, CRP.			
**Critical Care Medicine**						
Abbasi *et al*. (2020)[170]	Predict hemorrhage during ECMO	44 patients<br><br>(19 with bleeding)	**Demographics: race, BMI. Clinical: ECMO configuration, duration, indication and cannulation strategy, renal replacement therapy, drainage cannula size and site, transfusion of PLT or cryoglobulin, heparin drip rate. Lab: APTT, ACT, INR, PLT.**	RF, DT, KNN, LR	Leave-One-Out CV	DT performed best (accuracy 80%).
Chen et *al*. (2023)[42]	Predict bleeding risk in ICU patients receiving antithrombotic therapy	1,938 patients<br><br>(930 with bleeding)	Demographics: age, sex. Clinical: RR, **BP**, **HR**, **SpO_2**, CHF, arrythmias, VHD, PVD, neurological disease, SAH, COPD, DM, hypothyroidism, renal and liver disease, peptic ulcer, AIDS, lymphoma, solid tumor, rheumatoid arthritis, coagulopathy, alcohol. Lab: **thromboelastography values**, blood gases (**lactate**), **liver and kidney function tests**, **PLT**.	LSTM, GRU	Hold-out<br>Training: 80%<br>Testing: 10%<br>Validation: 10%	GRU predicts ICU bleeding risks faster and more accurately than clinicians (AUC 0.83, specificity 90%).
Taggart *et al*. (2018)[44]	Predict bleeding in clinical notes from ICU patients	1,650 clinical notes<br><br>(369 notes labeled as "bleeding present")	Text converted into numerical features: Term Frequency–Inverse Document Frequency n-grams for SVM and ET; word embeddings (GloVe) for CNN; rules-based approach used a bleeding dictionary with keywords or modifiers.	SVM, ET, CNN	Hold-out<br>Training: 60%<br>Testing: 40%<br><br>10-fold CV	RBNLP outperformed ML models (sensitivity 91.1%, specificity 84.6%, NPV 97.1%, PPV 62.7%).
**ENT**						

Ge *et al*. (2025)[171]	Predict secondary PTonH in pediatric patients	492 patients<br><br>(164 with PTonH)	Demographics: **age**, sex, BMI. Clinical: perioperative medications: antibiotics, **corticosteroids**, **NSAIDs**. Surgical details: **surgical technique** and instruments, **adenoidectomy**, postoperative fever, postoperative hospital stay. Lab: PT, **APTT**, INR, **FIB**.	DT, RF, XGB, SVM, KNN, LGBM, NB, LR	Hold-out<br>Training: 70%<br>Testing: 30%	XGB achieved the best performance (AUC 0.780, Sensitivity 0.739, Specificity 0.716, NPV 0.859, F1 Score 0.624).
Pizzi *et al*. (2001)[172]	Predict bleeding in tonsillectomy and adenoidectomy	191 patients<br><br>(89 with bleeding tendency)	**Bleeding tendency questionnaire data: frequency of bruising and nosebleeds, duration of nosebleeds, bleeding during past surgeries or injuries, bruise size from accidents/trauma, positive family bleeding history, joint bleeding history, menstrual bleeding duration (for female patients).**	SFN, SFN-FIQ, LDA	Hold-out<br>Training: 63%<br>Testing: 35%	SFN-FIQ outperformed standard SFN and LDA on bleeding questionnaire data, reaching 76% accuracy and showing moderate agreement (κ = 0.46).
**Gastroenterology**						
Agarwal *et al.* (2021)[39]	Predict bleed in esophageal varices in compensated advanced chronic liver disease	828 patients<br><br>(163 with variceal bleeding)	Demographics: age, gender. Clinical: etiology of liver disease. Lab: **Hb**, **PLT**, bil, creatinine, **INR**, **ALB**. Others: **MELD**, **liver stiffness measurement**, **endoscopic classification** (high-risk varices or low-risk varices).	XGB	Hold-out<br>Training: 77%<br>Testing: 23%<br><br>10-fold CV<br><br>EV (n=182)	XGB showed excellent performance (AUC 0.94 internal, 0.86 external), outperforming endoscopic classification (AUC 0.59).
Herrin *et al*. (2021)[173]	Predict GI bleeding in patients receiving antithrombotic therapy	306,463<br><br>(12,322 with GI bleeding)	Demographic: age, **sex**, race, ethnicity. Clinical: **alcohol** use, **smoking**, **AF**, **IHD**, **VTE**, DM, **SAH**, PAD, **chronic kidney**/liver **disease**, VHD, sleep apnea, viral hepatitis, rheumatologic disease, prior PCI, H *pylori*, **prior GI bleeding**, medications (anticoagulants, antiplatelets, **PPI/H2**, NSAIDs, antihypertensives, SSRI, antilipids).	RegCOx, RSF, XGB	Hold-out<br>Training: ~34.5%<br>Testing: ~65.5%<br><br>10-fold CV	RegCox outperformed HAS-BLED score in predicting 6-month (AUC 0.67 vs. 0.60) and 12-month (AUC 0.66 vs. 0.59) GIB risk.
Hong *et al*. (2023)[41]	Predict esophageal variceal bleeding based on endoscopic images	2,800 images	Endoscopic images of esophageal varices, with spatial and texture features automatically extracted by	MobileNet, ResNet, Xception, EfficientNet,	Hold-out<br>Training: 83%<br>Testing: 17%	EfficientNet achieved the highest accuracy (0.910 internal

		(1,400 with bleeding)	EfficientNet (**variceal size**, **red wale markings**, **vascular surface texture**).	ConvMixer, ViT	EV (n=400 images)	validation; 0.893 external testing), outperforming two expert endoscopists.
Hou *et al*. (2023)[174]	Predict esophagogastric variceal bleeding in liver cirrhosis	1,100 patients<br><br>(131 developed bleeding within 1 year)	**Demographics:** gender. Clinical: smoking, **drinking history**, **ascites**, **decompensation**. Endoscopic: location and **size of varices**, presence of **red wale markings**. Lab: ALT, GGT, **HCT**, **NLR**, **RBC**.	ANN	Training: 91%<br>Validation: 9%<br><br>(training and validation performed in two different hospitals)	ANN showed superior predictive performance (AUC 0.959 training, 0.945 validation) over NIEC (AUC 0.669/0.743) and Rev-NIEC (AUC 0.725/0.797) indices.
Larkin *et al*. (2024)[175]	Predict GI bleeding in hemodialysis patients	451,579 patients<br><br>(28,644 experienced GI bleeding)	Demographics: **age**, sex, race, ethnicity, marital status, height. Clinical: **dialysis** vintage and **access type**, chronic conditions (anemia, SAH, DM, cancer, CVD, COPD, CHF, hepatitis, drug or alcohol dependence, IHD, PVD, hyperparathyroidism), acute conditions (cardiac arrest, dysrhythmias, GI bleeding, infections), medications (heparin, VitD, calcimimetic, ESA, iron). HD treatment data: SBP, DBP, HR, temperature, pre-HD & post-HD weights, estimated dry weight, treatment time, clearance of Na, clearance Kt/V, blood flow rate, dialysate flow rate, saline administration, dialysis sessions interruptions. Lab: **Hb**, WBC, NEU, LYM, MO, EOS, BASO, PLT, Hb A1C, ALB, Ca, Cl, P, K, Na, creatinine,	XGB, LR	Hold-out<br>Training: 50%<br>Testing: 20%<br>Validation: 30%	XGB outperformed LR (AUC: 0.74 vs. 0.68).

			bicarbonate, BUN, BUN/creatinine ratio, **VitD**, **ferritin**, TSAT, parathormone. Environmental factors: season. Hospitalization events: LOS, **recent hospitalizations**, emergency room visits.			
Liu *et al*. (2022)[176]	Predict esophageal variceal bleeding	317 patients<br><br>(69 with bleeding)	Demographics: age, gender. Clinical: hepatic encephalopathy, CT (**esophageal varices diameter**, **splenic vein diameter**, **ascites**, **iodine concentration of spleen and short gastric vein**). **Radiomics features** extracted from liver, spleen, esophageal vein. Lab: **Hb**, PT, INR, creatinine, ALB, PLT.	Radiomics-based LR	Hold-out<br>Training: 70%<br>Testing: 30%<br><br>5-fold CV	Combined model (radiomics + CT + clinical) was the best-performing, with an AUC of 0.78, compared to CT + clinical (AUC 0.76) and radiomics-only (AUC 0.66).
Na *et al*. (2022)[177]	Predict bleeding after ESD in gastric cancer	5,629 patients<br><br>(325 with bleeding)	Demographics: **age**, **sex**. Clinical: **SAH**, DM, liver cirrhosis, **CKD**, medications (aspirin, **$P2Y_{12}$RA**, warfarin, **DOAC**, cilostazol, NSAIDs), pre-procedure management of antithrombotic, tumor characteristics (**size**, **location**, multiple), piecemeal resection. Lab: INR, ALB.	DL, MLR	Hold-out<br>Training: 80%<br>Testing: 20%	DL performed comparably to MLR (AUC 0.71 vs. 0.70).
Peng *et al*. (2024)[178]	Predict esophagogastric variceal bleeding in cirrhosis	208 patients<br><br>(92 with bleeding)	Demographics: age, sex. Clinical: **portal vein thrombosis**, **ascites**, cirrhosis's etiology, SAH, Child-Pugh score. Lab: **PT**, AFP, ALB, PLT, AST, ALT, GGT, Tbil, Dbil, Ibil. **Radiomics features** selected from liver, spleen, and esophagogastric fundus, including shape, first-order and texture features.	LASSOR models: Rad-score, Rad-score fusion model; MLR-based model: clinical, radiomics-clinical	Hold-out<br>Training: 70%<br>Testing: 30%	Radiomics-clinical performed best (AUCs of 0.930, sensitivity 82.1%, specificity 85.7%).

Seo *et al*. (2020)[179]	Predict rebleeding in stable non-variceal UGIB	1,439 patients<br><br>(134 experienced rebleeding)	Demographics: age, sex. Clinical: SBP, DBP, HR, RR, temperature, SpO_2, DM, SAH, cardiac disease, liver disease, coagulopathy, IHD, HF, previous UGIB, neoplasm, CKD, COPD, stroke, mental status, syncope, hematemesis, melena, **fresh blood on nasogastric tube**, medications (NSAIDs, (antiplatelet agents, anticoagulants). Lab: **Hb**, **PLT**, **INR**, **BUN**, creatinine, **ALB**, **lactate**, **BD**.	RF, GB, VC, LR	5-fold CV	VC performed best (AUC 0.733), outperforming GBS (AUC 0.694) and the Rockall Score (AUC 0.550).
Wang *et al*. (2022)[40]	Predict esophageal variceal bleeding	341 patients<br><br>(121 experienced bleeding)	Demographic: age, sex, BMI. Clinical: **alcohol use**, hepatic encephalopathy, ascites, etiology of cirrhosis (HBV, HCV, others). Image: **endoscopic features**. Lab: PLT, creatinine, HDL, **PT**, INR, **ALT**, AST, **Tbil**, ALB, Child-Pugh score, MELD, FIB-4, APRI.	XGB, RF, GBM, GLM, Stacking Ensemble, DL (ResNet, ViT, Xception, EfficientNet, ConvMixer)	Hold-out<br>Training: 70%<br>Testing: 30%<br><br>EV (n=161)	Stacking model performed best (AUC 0.975, sensitivity 0.952, and accuracy of 0.932 in the external test set), outperforming MELD and Child-Pugh scores.
Wong *et al*. (2019)[180]	Predict recurrent bleeding in idiopathic gastroduodenal ulcer	24,119 patients<br><br>(4,772 with recurrent bleeding)	Demographics: **age**, gender. Clinical: **ulcer location** (**gastric**, duodenal), GI diseases, **malignancies**, **infections**, aspirin, NSAID, anticoagulants. Lab: **Hb**, HCT, WBC, PLT, PT, BUN, creatinine.	LR, RidgeR, AdaBoost, DT, RF	Hold-out<br>Training: 95%<br>Testing: 5%<br><br>(EV n=1,265)	LR performed best (AUC 0.775, accuracy 84.3%, NPV 99.1%).
Yan *et al*. (2022)[181]	Predict esophageal variceal bleeding	391 patients<br><br>**109 mild Var vs. 109 non-Var (n=218)** and **120 high-risk Var vs. 120 mild Var (n=240)**	2,358 radiomic features extracted from contrast-enhanced CT images (**textural, statistical, wavelet, and HOG features** from **esophagus, liver, and spleen)**.	SVM	5-fold CV<br><br>EV (n=405)<br>**94 mild Var vs. 65 non-Var (n=159)** and **246 high-risk Var vs. 94 mild Var (n=340)**	SVM-based radiomic model achieved AUC 0.834 (test), 0.736 (external), and outperformed Baveno VI criteria in accuracy and reclassification.

Zhao *et al*. (2025)[182]	Predict esophageal variceal bleeding in patients with cirrhosis	224 patients<br><br>(112 with bleeding)	Demographics: **age**, height, weight, BSA. Clinical: hepatitis, ALD, autoimmune injury, other diseases. CT-derived imaging features (liver and **spleen volume**, diameter of esophageal varices). Lab: **ALT**, **AST**, ALB, TBil, PT, APTT, TT, **FIB**, **DD**, **PLT**, fibrosis-4 index.	LR, RF, SVM, AdaBoost	Hold-out<br>Training: 80%<br>Testing: 20%<br><br>5-fold CV	RF performed best (AUC 0.818).
Zhuang *et al*. (2023)[183]	Predict rebleeding after acute upper GI bleeding	1170 patients<br><br>(135 with rebleeding)	Lab: lactic acid, NEU%, **PLT**, ALB, **DD**.	LR, XGB (for variable importance ranking)	Hold-out<br>Training: 77%<br>Testing: 23%	The new prediction model outperformed the AIMS65 score (AUC 0.746 vs. 0.619).
**General Surgery**						
Chen *et al*. (2018)[184]	Predict postoperative bleeding in colorectal surgery	13,399 surgeries<br><br>(1,680 experienced postoperative bleeding)	Demographics: age, sex, race, BMI. Patient reported symptoms: **alcohol use, anesthesia complication, bleeding complication, difficulty performing daily activities, pain, nausea, weakness. Comorbidities: DM, SAH, CAD, HF, COPD, pulmonary disease, kidney disease, benign and malignant rectal/colon cancer, hemophilia, embolism, pancreatitis, colitis, diverticulitis, anemia. Physiological parameters: SBP, DBP, MAP, HR, RR, temperature, SpO_2. Lab: Hb, HCT, PLT, creatinine, BUN, glucose, Na, K, Ca, AST, Tbil, Dbil, ALB, troponin. Observational assessments: activity level, ASA, mobility, pain levels, fall risk score, nutritional status. NLP-derived variables. Anticoagulants.**	LR, GBM	10-fold CV	GBM outperformed LR (AUC: 0.822 vs. 0.735), while also identifying functional status variables as novel predictors.

			**Procedure: type of surgery, intraoperative complications, surgery length. Postoperative variables: incision condition, bowel sounds, urine clarity, postoperative pain, nutrition, mobility.**			
Hsu *et al*. (2023)[185]	Predict postoperative GI bleeding in bariatric surgery	159,959 patients<br><br>(632 with GI bleeding)	**Demographics: age**, gender, weight, BMI, race, ethnicity. Clinical: ASA, functional status, SAH, hyperlipidemia, GERD, DM, COPD, smoker, sleep apnea, PE, DVT, MI, prior PCI/PTCA, prior cardiac or foregut surgery, venous stasis, IVC filter, dialysis, renal insufficiency, medications (anti-hypertensive, anticoagulation, steroids and other immunosuppressants). **Surgical procedure:** type of bariatric surgery, surgical approach, **procedure type**, robotic assist, **operative duration**, surgical specialty, lysis of adhesions, EGD, anastomosis/stale line leak test, paraeshopageal hernia repair, gastric band removal, cholecystectomy, nerve block, liver biopsy, omental flap. **Lab: HCT**, **creatinine**, HbA1c, ALB.	RF, XGB, NN, LR	Hold-out<br>Training: 80%<br>Testing: 20%<br><br>5-fold CV	RF performed best (AUC 0.764).
Ikuta *et al*. (2024)[186]	Predict post pancreatectomy hemorrhage following pancreaticoduodenectomy	284 patients<br><br>(11 experienced hemorrhage)	**Demographics: age**, **sex**, weight, **BMI**. Clinical: PNI, smoking, ASA, **SAH**, DM, PDAC, IPMN, ampullary carcinoma, cholangiocarcinoma, NET, **pancreatic duct diameter**, preoperative biliary drainage. **Surgical procedure:** surgery type,	ETC, RF, LR, LGBM	10-fold CV<br><br>Bootstrap resampling (1,000 iterations) for model stability	ETC showed the best performance, with high accuracy across all validations (F1 0.937 training, 0.941 test, 0.901 bootstrap; AUC 0.985).

			venous resection, hepatic artery resection, operative time, estimated blood loss, intraoperative transfusion, drain amylase levels, presence of postoperative pancreatic fistula. **Lab: CRP, WBC, Hb, ALB,** eGFR, PLT, **NLR**, PLR.			
Li *et al*. (2024)[187]	Predict massive intraoperative bleeding in patients undergoing liver resection for primary hepatic malignancies	406 patients<br><br>(65 experience massive bleeding)	**Clinical: ascites, alcohol consumption, TNM staging. Lab: ALB-bil score.**	LR, KNN, DT, RF, NB	Hold-out<br>Training: 70%<br>Testing: 30%	**LR** was the best performer (**AUC 0.803).**
Liu *et al*. (2023)[188]	Predict intraoperative major bleeding in liver resection	936 patients<br><br>(339 with major bleeding)	Demographics: age, gender, weight. Clinical: ASA, DM, liver cirrhosis, COPD, SAH, previous surgeries, LVEF. Surgical procedure: **surgical duration**, **Pringle maneuver duration**, anesthetic agents. Lab (pre- and intraoperative): **pre_Hb, intra_HCT**, PLT, **intra_lactate**, **pre_AST**, ALT, ALB.	XGB, LR	Hold-out<br>Training: 80%<br>Testing: 20%<br><br>5-fold CV	XGB outperformed LR (accuracy 0.80 vs. 0.76; precision 0.89 vs. 0.79).
Loftus *et al*. (2017)[189]	Predict severe lower intestinal bleeding and the need for surgical intervention	147 patients<br><br>(61 experienced severe bleeding)	Clinical: **SBP**, **aspirin use**, CCI. Lab: BD, INR, **Hb**, HCT.	MLP	Hold-out<br>Training: 70%<br>Testing: 30%	ANN outperformed the Strate rule in predicting severe bleeding (AUC 0.979 vs. 0.656).
Park *et al*. (2022)[190]	Predict massive intraoperative blood loss in liver transplant surgery	414 patients<br><br>(124 experienced massive blood loss)	Demographics: age, sex. Clinical: **MAP**, **pulse pressure**, **temperature**, **MELD**, **hepatocellular carcinoma status**, etiology of liver disease (hepatitis B and C, alcohol-related, autoimmune, cryptogenic, others). Surgical procedure: **operation**	LR, NN, SVM, RF, XGB, EN	Hold-out<br>Training: 70%<br>Testing: 30%<br><br>5-fold CV	LR performed best (AUC 0.84).

			**duration**. Lab: **APTT**, **creatinine**, ALB, BUN.			
Wakiya *et al*. (2021)[191]	Predict massive intraoperative bleeding in pancreatic cancer surgery	175 patients<br><br>(88 experienced massive bleeding)	**Demographics: age, gender, weight, BMI. Clinical: DM, cancer history, SAH, heart disease, CVD, hepatitis, obstructive jaundice, biliary drainage. Surgical procedure: type of surgery, portal vein resection. Lab: WBC, Hb, HCT, PLT, CRP, ALB, TP, creatinine, AST, ALT, GTP, Tbil, amylase, CA19-9, CEA, tumor factors (stage, TNM category, UICC stage).**	CART, LR	Hold-out<br>Training: 73%<br>Testing: 27%	CART outperformed LR with higher accuracy (80.9% vs. 70.3%) and sensitivity (100% vs. 75%).
Weller *et al*. (2018)[192]	Predict post-surgical complications (including bleeding) in colorectal surgery	1,353 procedures<br><br>(185 experienced bleeding)	**Demographics: age, sex. Clinical: ASA, preoperative diagnosis. Surgical procedure: surgery duration, procedure type, wound type, blood loss, fluid administration. Lab: Hb, ALB, HCT, ALT. NLP-derived features.**	LASSOR, RF, SVM, NB, AdaBoost	Hold-out<br>Training: 80%<br>Testing: 20%<br><br>5-fold CV	**RF** was the best-performing method for predicting **bleeding complications** (**AUC of 0.86).**
Xue *et al*. (2021)[193]	Predict intraoperative bleeding in liver cancer patients undergoing hepatectomy	665 patients<br><br>(105 experienced intraoperative bleeding)	**Demographics: age, sex. Clinical: tumor size and stage. Surgical procedure: operation time, intraoperative bleeding volume, medications (midazolam, cis-atracurium, dexmedetomidine, sevoflurane. Lab: Hb, HCT, RBC, WBC, PLT, RDW, PDW, MO, LYM, NEU, ALB, GLO, Ca.**	DT, XGB, LinearSVC, MLPC, KNN, CNN, LSTM, CNN-LSTM, PBNN, LR	Hold-out<br>Training: 70%<br>Testing: 30%<br><br>5-fold CV	XGB performed best (AUC 0.728, accuracy 87%, precision 100%).
**Hematology**						
An *et al*. (2023)[194]	Predict life-threatening bleeding in ITP	3,191 patients (2,094 retrospective,	Demographics: **age**, gender. Clinical: **ITP type**, comorbidities and symptoms (**CVD**, COPD, CKD, **uncontrolled DM**, **infection**, **skin**	RF, XGB, LR, MLP, AdaBoost, SVM, LGBM	Hold-out<br>Training:71%<br>Testing: 29%	RF achieved best AUC: 0.89 internal validation, 0.82 prospective inpatient validation;

		1,097 prospective)<br><br>(647 experienced bleeding)	**and mucosa bleeding**), treatments within 1 month (danazol, (glucocorticoid, immunosuppressive agents, Ig, Rituximab). Lab: **PLT**, **LYM**.		CV performed (folds' number not specified)<br><br>EV (n=1,097 - prospective cohort)	model available online as a web-based tool.
Chu *et al*. (2023)[195]	Predict ICH in acute leukemia	948 patients<br><br>(75 experienced ICH)	Demographics: age, sex. Clinical: smoking, drinking, SAH, hyperglicemia, stroke, leukemia type. Lab: WBC, NEU, LYM, **MO**, RBC, Hb, **PLT**, APTT, **PT**, **INR**, **FIB**, DD, Dbil, **Ibil**, **ALB**, GGT, AST, ALT, ALP, **pre-ALB**, BUN, **creatinine**, uric acid, CK, CK-MB, **LDH**, total cholesterol, HDL, LDL, triglycerides.	RF, LR, SVM, KNN, DT, NB, Gaussian NB, AdaBoost	Hold-out<br>Training: 80%<br>Testing: 20%<br><br>10-fold CV	RF outperformed all other models (AUC 0.995).
Kang *et al*. (2025)[196]	Predict post-procedure RBC transfusion and to evaluate whether PLT transfusion reduces bleeding risk in patients with ITP	3,848 patients<br><br>(608 transfused)	Demographics: **age**, sex, socioeconomic status. Clinical: **ITP phase**, treatment history (steroids, Ig or anti-D, TPO-RA, Rituximab, **PLT transfusion amount**), splenectomy, **anemia**, CKD, liver cirrhosis, cancer, SAH, DM, CHF, cardiovascular disease, PVD, COPD, connective tissue disease, peptic ulcer disease, dementia, MI, hypothyroidism, hemiplegia, thyrotoxicosis, AIDS. Procedure: **bleeding risk**, antiplatelet/ **anticoagulant use**.	RF	Hold-out<br>Training: 80%<br>Testing: 20%<br><br>CV performed (folds' number not specified)	RF achieves string performance, with an AUC of 0.936, accuracy of 87.1% and F1-score of 0.861).
Sidonio Jr et al. (2024)[197]	Identify undiagnosed symptomatic patients with VWD	4,875,499 patients	Demographics: age, sex. Clinical: bleeding symptoms (**heavy menstrual bleeding**, **epistaxis**, GI and **respiratory bleeding**), procedures (cauterization, IUD	RF (for females), GB (for males)	Hold-out<br>Training: 80%<br>Testing: 20%	RF (84% accuracy) in females and GB (85%) in males identified **48,902 likely undiagnosed VWD cases** with high

		(5,981 with symptomatic VWD)	placement), healthcare utilization (**ER visits**, hospitalizations), medications (**DDVAP**, **TXA**), comorbidities (**anemia**, SAH). Lab: **APTT**, VWF assays.			precision (PPV: 93%/92%, sensitivity: 73%/77%).
**Internal Medicine**						
Bernardini *et al*. (2024)[198]	Predict outcomes in anticoagulated patients with atrial fibrillation	11,078 patients<br><br>(388 experienced major bleeding)	Demographics: **age, BMI**. Clinical: AF, SAH, DM, HF, CAD, PAD, COPD, cancer, dementia, anemia, frequent fall, **previous bleeding**, previous stroke, antiplatelet drugs, anticoagulation type, CHA_2DS_2VASc score, HAS-BLED score. Lab: **eGFR**, **Hb**, **PLT**, RBC.	SLR, GBDT, MMoE, MTNN	5-fold CV	GBDT best predicted major bleeding in DOAC users (AUC = 0.711); ML models outperformed HAS-BLED score.
Chen *et al*. (2023)[199]	Predict bleeding risk in geriatric patients on rivaroxaban	798 patients<br><br>(112 experienced bleeding)	Demographics: age, gender, **BMI**. Clinical: dose of rivaroxaban, antiplatelet drugs, SAH, DM, coronary disease, HF, valvopathy, PCI, apoplexy, **hemorrhage history,** coagulopathy. Lab: triglyceride, cholesterol (total, LDL), **Hb**, **PLT**, **TT**, **APTT**, INR, **DD**, ALT, AST, BUN, **creatinine**.	LR, RF, XGB	Hold-out<br>Training: 85%<br>Testing: 15%<br><br>10-fold CV<br><br>EV (n=94)	XGB outperformed LR and RF: AUC = 0.776 (internal), 0.689 (external).
Chen *et al*. (2024)[200]	Predict bleeding risk in elderly aspirin users	26,030 patients<br><br>(1,565 experienced bleeding)	Demographics: age, sex. Clinical: smoking, alcohol use, dual antiplatelet therapy, DM, SAH, MI, PCI, operation history, **tumor histology**, **cerebral infarction**, **previous bleeding**, **gastric ulcer**, use of gastric protective medicine, portal hypertension, anticoagulants use. Lab: **Hb**, **PLT**, WBC.	XGB, LASSOR, MLR	Hold-out<br>Training: 70%<br>Testing: 30%<br><br>10-fold CV	XGB using six clinical predictors achieved strong discrimination (AUC 0.820).

Fard *et al*. (2024)[201]	Predict bleeding risk in VTE	2,542 patients<br><br>(118 had major bleeding)	Demographics: **age**, **sex**. Clinical: VTE characteristics (DVT, PE, **provoked VTE**), DM, post-thrombotic syndrome, prior stroke, GI bleeding history, medications (**number of concomitant medications**, **antiplatelet** and anticoagulant agents), genetics (**CYP2C9 polymorphisms**, VKORC1 genotype, CYP4F2 variant, Factor V Leiden and prothrombin genes' mutations). Lab: **Hb**, **creatinine**.	LR, LDA, QDA, Gaussian NB, SVM, RF, AdaBoost, GB	5-fold CV	ML models did not outperform clinical models; QDA was the best ML model (AUC 0.67) but still limited in discrimination.
Fard *et al*. (2024)[202]	Predict major bleeding in patients on extended anticoagulation	2,542 patients<br><br>(118 had major bleeding)	Demographics: age, sex, weight, BMI. Clinical: VTE characteristics (DVT, PE, provoked VTE), **smoking**, DM, **SAH**, prior stroke, GI bleeding history, medications (**number of concomitant medications**, antiplatelet and anticoagulant agents), genetics (CYP2C9 polymorphisms, VKORC1 genotype, CYP4F2 variant, Factor V Leiden and prothrombin genes' mutations). Lab: Hb, creatinine.Time-dependent: clinical events, **treatment changes**, medications, surgeries, lifestyle, **temporal markers**.	FFNN, ANN, LSTM, Ensemble (FFNN-ANN)	Hold-out Training: 70% Testing: 30%<br><br>5x2 CV	Ensemble model performed best (AUC 0.824, Sensitivity 61%, Specificity 82%), outperforming 6 clinical models (HAS-BLED, CHAP, RIETE, ACCP, VTE-BLEED, OBRI).
Mora *et al*. (2023)[63]	Predict major bleeding in patients on anticoagulation for VTE	49,587 patients<br><br>(873 had major bleeding)	Demographics: age, sex, weight. Clinical: alcohol, HR, SBP, MI, stroke, PAD, SAH, esophagitis, hiatal hernia, esophageal varicosities, gastroduodenal ulcer, gastric erosions, ulcerative colitis, Chron's disease, angiodysplasia, liver cirrhosis, liver disease, dementia, hemoptysis, right ventricular hypokinesis, **active cancer**, prior VTE or PE, post-partum, thrombophilia, syncope, medications (NSAIDs, antiplatelet drugs, corticosteroids,	XGB, SVM, DT, NN, KNN	Hold-out Training: 70% Testing: 30%<br><br>10-fold CV<br><br>Prospective validation cohort: 10,337 (227 had major bleeding)	XGB outperformed RIETE and VTE-BLEED scores in internal (AUC 0.91) and prospective validation (F1 score 15.4%) but underperformed in EV (F1 score 5.2%).

			**anticoagulation**). Procedural: VTE diagnosis site, hospitalization status, recent surgery, recent immobilization, **history of bleeding**. Lab: **Hb**, **PLT**, WBC, DD, **creatinine**.		EV (n=3,027)	
Qian *et al*. (2024)[64]	Predict bleeding in dual antiplatelet therapy	18,408 patients<br><br>(1,075 experienced bleeding)	Demographics: age, **sex**. Clinical: smoking, drinking, DM, **SAH**, **previous bleeding**, **cerebral infarction**, **surgical history**, tumor history, MI, PCI, gastric ulcer, portal hypertension, medications (anticoagulant use, gastric protective). Lab: Hb, PLT, WBC.	LASSO, LR, RF, KNN, DT, XGB, LGBM, SVM	Hold-out<br>Training: 70%<br>Testing: 30%<br><br>10-fold CV	XGB performed best (AUC 0.877)
Watanable *et al*. (2021)[203]	Predict outcomes (including major bleeding) in AF	7,406 patients<br><br>(140 had major bleeding)	Demographics: **age**, sex, height, weight. Clinical: SBP, DBP, HR, alcohol use, CHF, SAH, DM, stroke or TIA, CAD, COPD, cardiomyopathy, malignancy, CHD, hyperthyroidism, abnormal renal and liver function, history of cancer, **history of bleeding**, **labile INR**, medications (warfarin, antiplatelet agents, statins, digitalis, Beta-blockers, calcium channel blockers, **antihypertensive drugs**, **ACE inhibitors/ARBs**. Lab: Hb, PLT, **creatinine**, **ClCr**, total cholesterol, Tbil, AST, **ALT**.	LR, RF	Hold-out<br>Training: 80%<br>Testing: 20%<br><br>5-fold CV	RF achieved an AUC of 0.69, performing similarly to LR (AUC 0.66) and ORBIT (AUC 0.67), but significantly outperforming HAS-BLED (AUC 0.61) and ATRIA (AUC 0.62) scores.
**Neurosurgery**						
Cui *et al*. (2022)[204]	Predict bleeding after thrombolysis in acute ischemic stroke	415 patients<br><br>(Not explicitly stated)	**Demographics: age, gender. Clinical: smoking, alcohol, AF, stroke, DM, time from stroke onset to treatment, SBP, DBP, NIHSS, TOAST classification. Imaging (radiomic)**	LR, RF, SVM, XGB	Hold-out<br>Training: 80%<br>Testing: 20%<br><br>EV (n=102)	XGB achieved highest performance: AUC = 0.945 (internal), 0.914 (external).

			**features. Lab: Hb, fasting blood sugar, INR, MO.**			
Dharmasaroja *et al*. (2012)[205]	Predict bleeding after thrombolysis in ischemic stroke	190 patients<br><br>(not explicitly stated)	**Demographics: age, sex. Clinical: NIHSS, Stroke subtype (TOAST criteria) and location, DBP, SBP, t**ime from symptom onset to rtPA administration, AF, DM, CHF, brain **CT findings**. Lab: Hb, WBC, **PT**, INR, **LDL**, PLT, glucose.	MLP, RBF, PNN, SVM	10-fold CV	PNN achieved the highest AUC (0.788); all ANN models performed similarly (AUC > 0.50).
Heo *et al*. (2024)[206]	Predict HT following reperfusion therapy in ischemic stroke	362 patients<br><br>(218 had HT)	**Demographics: a**ge, sex. Clinical: DM, dyslipidemia, AF, CHF, infarct location, NIHSS, antiplatelets/anticoagulant and statin use. Procedure: thrombolysis, endovascular thrombectomy. Lab: Hb, HCT, PLT, WBC, LDL, glucose. **Radiomic Features: first-order statistics**, **textural** matrices, multi-resolution transformations.	LGBM, ET, LR	Hold-out<br>Training:70%<br>Testing: 30%<br><br>5-fold CV	**LGBM (with all radiomics features) performed best: AUC 0.986.**
Hu *et al*. (2025)[207]	Predict HT after mechanical thrombectomy in acute ischemic stroke	159 patients<br><br>(100 experienced HT)	**Demographics: sex. Clinical: CAD, alcohol. Stroke and procedural characteristics (**NIHSS score, number of stents passes, **anterior circulation involvement**, thrombolysis, **HUmax ≥90** on initial CT), **ASPECTS**. **Radiomic features**. Imaging markers (presence of **subarachnoid hyperattenuated imaging marker**).	SVM, GB, XGB, LGBM, RF, KNN, AdaBoost	Hold-out<br>Training: 80%<br>Testing: 20%<br><br>5-fold CV	Combined clinical-radiomics model (SVM) had the highest AUC (0.925), outperforming clinical and radiomics models alone.
Li *et al*. (2023)[208]	Predict HT after ACI	1,182 patients<br><br>(587 with HT)	Demographics: age, sex. Clinical: SAH. Lab: RBC, RDW, Hb, MCV, MCH, MCHC, HCT, WBC, **NEU**, NEU%, PLT, MPV, PDW, TP, ALB, ALB/GLO ratio, BUN, **uric acid**, **creatinine**, Tbil, Dbil, Ibil, total bile	XGB, LR, DT, RF	5-fold CV<br><br>EV (n=227)	XGB performed best (AUC 0.95-0.96).

			acid, cholesterol (total, LDL, HDL), triglycerides, ALT, AST, ALP, γ-GT, cholinesterase, **glucose**, **Pre-BNP**, **CRP**, CK-MB, **myoglobin**, **Ca**, K, Cl, Na, **CO_2 combining power**, PT activity, PT, PT ratio, FIB, **TT**.			
Li *et al*. (2024)[209]	Predict the risk of (re)hemorrhage in sporadic CCM	517 patients<br><br>(76 with re-hemorrhage)	**Demographics: age**, sex. Clinical: **CCM volume** and **location**, SAH, DM, hypercholesterolemia, **prior ICH**, associated development venous anomaly.	XGB, SVM, SGM	Hold-out<br>Training: 80%<br>Testing: 20%<br><br>5-fold CV	XGB performed best (AUC 0.83, AUC-PR 0.40, sensitivity 79%, specificity 72%).
Lin *et al*. (2025)[210]	Predict HT after intravenous thrombolysis in acute ischemic stroke	1,007 patients<br><br>(99 with HT)	**Demographics: age, sex. Clinical: SAH, DM, AF, CAD, stroke, smoking, alcohol, antiplatelet therapy, SBP, DBP, onset-to-treatment time, NIHSS, tPA dose. Imaging features: hyperdense middle cerebral artery sign, massive cerebral infarction, ASPECTS score. Lab: WBC, PLT, APTT, TT, PT, INR, FIB, glucose.**	RF, MLP, AdaBoost, Gaussian NB	Hold-out (split not explicitly stated)<br><br>10-fold CV	RF performed best in EV (AUC 0.712, accuracy 0.867, F1 0.929), outperforming traditional scores.
Raman *et al*. (2020)[211]	Predict intraoperative blood loss in adult spinal deformity	909 patients<br><br>(Not explicitly stated)	**Demographics: age, sex, BMI. Clinical: ASA, MFI, DM, SAH, cardiovascular disease, COPD, osteoporosis. Surgical procedure: number of levels fused**, **pelvic fixation**, **3-column osteotomy**, **posterior column osteotomy**, surgical duration, Mirza Invasiveness Index, blood loss, cell salvage, TXA, RBC transfusion. Lab: **Hb**.	CIT, MLR	Not explicitly stated	CIT identified high-risk subgroups: R^2-Adj for bleeding prediction = 0.64 (>13 levels), 0.58 (≤6), 0.51 (6–13 levels).

Ren *et al*. (2025)[212]	Predict HT after thrombolysis in AIS	445 patients<br><br>(202 with HT)	**Demographics: age, sex. Clinical: NIHSS, SBP, DBP, history of stroke, DM, AF, time from onset to CT, non-contrast CT images. Lab: PLT, WBC, NEU, LYM, MO, EOS, Hb, glucose, NLR, LMR.**	XGB, DenseNet50-based CNN, XGB+ DenseNet50-based CNN	Hold-out<br>Training: 77%<br>Testing: 23%<br><br>5-fold CV<br><br>EV (n=101)	Ensemble model outperformed clinical scores, including MSS, SEDAN and GRASPS (AUC 0.937, sensitivity 0.878, specificity 0.883).
Ru *et al*. (2023)[213]	Predict HT after thrombolysis in AIS	828 patients<br><br>(69 with HT)	**Demographics: age, gender. Clinical: SBP, SAH, temperature, DM, AF, hypercholesterolemia, smoking, stroke, antiplatelets/anticoagulant use, rt-PA/urokinase, NIHSS, symptom onset to treatment. Imaging: baseline NCCT brain scans. Lab: PLT, glucose.**	LR, SVM, KNN, RF, XGB, WSDL, BDL	3-fold CV	WSDL achieved highest AUC (0.799); better predictive value than HAT and SEDAN clinical scores.
Saeedi *et al*. (2024)[214]	Predict IVH in premature neonates	160 neonates<br><br>(70 with IVH)	Demographics: **gestational age**, gender. Clinical: **birth weight**, **Apgar scores**, **head circumference**, **delivery method**. Lab: **Hb**, **PLT**, **APTT**, **INR**, **Tbil**, **Dbil**, **BUN**, **ESR**. Imaging: IVH grade.	RF, SVM, LR, KNN	Hold-out<br>Training: 80%<br>Testing: 20%<br><br>10-fold CV	RF has the best performance (AUC 0.99, accuracy 87%, sensitivity 75%, specificity 100%).
Saggi *et al*. (2022)[215]	Predict bleeding in pediatric bAVM	186 patients<br><br>(116 with bleeding)	Demographics: **age**, sex. Clinical: neurological deficits, seizures. Imaging: **bAVM size** and **location**, venous drainage pattern, nidus diffuseness, eloquence, hemorrhagic presentation, laterality, concurrent arterial aneurysm.	RF, GBDT, AdaBoost, LR	Not explicitly stated	ML, particularly GBDT, outperformed LR, identifying bleeding risk factors that conventional analysis missed.
Shi *et al*. (2024)[216]	Predict massive blood loss in metastatic spinal disease	276 patients	**Demographics: age, gender. Clinical: ECOG score, tumor type, smoking, number of comorbidities, renal disease, coronary disease, DM, SAH,**	LR, XGB, RF, KNN, DT, SVM	Hold-out<br>Training: 70%<br>Testing: 30%	XGB achieved the best performance (AUC = 0.857 internal, 0.809 external).

		(69 with massive blood loss)	**liver disease, visceral metastases, extravertebral bone metastases, oncological therapies (preoperative chemotherapy, targeted therapy, endocrinology). Surgical procedure: preoperative embolization, surgical process, surgical site, number of surgical segments. Lab: ALB, Hb, PT, APTT, PLT.**		Bootstrapping (with 100 repetitions)<br><br>EV (n=76)	
Turcato *et al*. (2022)[217]	Predict post-traumatic ICH in patients on anticoagulant therapy	3,054 patients<br><br>(290 developed ICH)	**Pre-trauma:** age, antiplatelet use, intoxication, dementia/psychiatric history, epilepsy, **prior neurosurgery**. Post-trauma: **TLOC**, **amnesia**, **headache**, **GCS**, **vomiting**, seizure, **visible trauma above clavicles**, neurological deficit. Lab: glucose, PLT.	CART	Not explicitly stated	CART stratified ICH risk from 2.5% to 61.4% and excluded low-risk cases with 98.4% accuracy.
Yang *et al*. (2022)[218]	Predict perioperative hidden blood loss in thoracolumbar burst fracture patients	161 patients<br><br>(62 with hidden blood loss)	**Demographics: age, sex, BMI. Clinical: smoking, alcohol use, SAH, DM, COPD, previous transfusion, chronic steroid use. Surgical procedure: operation time, time from admission to surgery, number of fused vertebrae, intraoperative infusion of crystalloids and colloids, transfusion (autologous/allogenic). Imaging: Beta. Lab: HCT, Hb, ALB, FIB, APTT, Na, K, Ca.**	RF, XGB, LGBM, AdaBoost, NB, SVM, KNN, MLP, LR	15-fold CV	RF performed best (AUC 0.864).
Zernikow *et al*. (1998)[219]	Predict severe IVH in preterm neonates	865 patients<br><br>(108 experienced severe IVH)	Clinical: **gestational age**, **birthweight**, **1- and 5-minute Apgar scores**, **transport duration**, cesarean delivery, mechanical ventilation, premature rupture of membranes,	ANN, LR	Hold-out<br>Testing: 50%<br>Training: 50%	ANN outperformed LR (AUC 0.935 vs. 0.884).

			**emergency delivery**. Lab: **capillary pH**, $PaCO_2$.			
Zhu *et al*. (2024)[220]	Predict IVH in preterm infants	862 patients<br><br>(431 experienced IVH)	Demographics: maternal age. Clinical: history of obstetric complications, **dexamethasone** use. Lab results (maternal): Hb, PLT, **Tbil**, **RDW**, CRP, **TT**, **HBeAg** and **anti-HBe**, **anti-HCV**, **anticardiolipin antibody**. Neonatal /Delivery: **gestational age**, **birthweight** (the study does not list all 1,080 variables explicitly).	RF	Hold-out<br>Training: 80%<br>Testing: 20%	RF achieved an AUC of 0.88, with 74% sensitivity and 85% specificity.
**Nephrology**						
Nopp *et al*. (2022)[221]	**Predict bleeding risk in HD**	625 patients<br><br>(89 developed major bleeding)	**Demographics: age, sex, BMI. Clinical: smoking status, AF, DM, HF, CAD, PAD, stroke or TIA, prior major bleeding, history of bleeding, cancer history, MI, venous thromboembolism, artificial heart valve, SAH. Dialysis parameters: tine on HD, ultrafiltration rate, remaining diuresis, kidney transplant, peritoneal dialysis. Lab: Hb, HCT, PLT, WBC, ALB, CRP.**	KNN, DT, RF, NN	100-fold Monte Carlo CV	Neither ML models nor bleeding scores effectively predicted bleeding risk in HD (AUC 0.49–0.55).
**Obstetrics**						
Ahmadzia e*t al*. (2024)[222]	Predict PPH and transfusion	185,413 patients<br><br>(5,760 experienced PPH)	Demographics: age, ethnicity, education, marital status, insurance, BMI, gravidity, parity. Clinical: obstetric surgery (prior cesarean delivery, macrosomia, stillbirth, preterm birth, shoulder	LR, SVM, MLP, RF, GB, TFIM, Emb	Hold-out<br>Training: 70%<br>Testing: 30%	GB performed best (AUC 0.833).

			dystocia, uterine scar, STD, thyroid disease, anemia, asthma, heart disease, renal disease, seizure disorder), prenatal conditions (DM, gestational and chronic hypertension, preeclampsia, oligohydramnios, placentae previa and accreta, bleeding in 3rd trimester, cervical incompetence, fetal growth restriction, fetal macrosomia, antenatal hospital admission, chorioamnionitis, UTI, preterm birth, smoking, alcohol, recreational drug use), intrapartum (reason for admission, Bishop score, SBP, DBP, TOLAC, **mode of delivery**, **oxytocin incremental dose**, **tocolytic use**, **anesthesia type**, Mg sulfate use, **hospital type**, **presence of anesthesia nurse**, indication for labor, fetal presentation).			
Akazawa *et al*. (2021)[223]	Predict PPH in vaginal delivery	9,894 births<br><br>(188 experienced PPH)	Demographics: **age**. Clinical: parity, **maternal** height and **weight before pregnancy**, **maternal weight on admission of labor**, pregnant gestation of labor. Neonatal characteristics: birth weight, sex of the baby. Delivery characteristics: fetal position (cephalic or breech), oxytocin use before delivery, mode of delivery (spontaneous, vacuum, or forceps).	DLNN, LR, SVM, RF, XGB, DT	Hold-out<br>Training: 80%<br>Testing: 20%<br><br>5-fold CV	LR outperformed all other models, achieving the highest AUC (0.708) and accuracy (0.686).
Akazawa *et al*. (2023)[224]	Predict severe hemorrhage (>2000mL) in placenta previa	48 patients	Demographics: age. Clinical: **gravity**, parity, previous cesarean section, **gestation of delivery (week)**, **weight**	DL (VGG16-CNN for images and NN for tubular data),	Hold-out<br>Training: 80%<br>Testing: 20%	DL achieved best accuracy (0.68) and AUC (0.735); better than

		(26 with severe hemorrhage)	**of neonate**. Lab: Hb, PLT, WBC. Others: **placental position on MRI**.	VGG16-CNN (image only), XGB (tubular only)	5-fold CV	human experts (mean accuracy 0.614).
Chen *et al*. (2024)[225]	Predict bleeding risk during cesarean scar ectopic pregnancy	1680 patients<br><br>(197 experienced bleeding)	Demographics: gestational age. Clinical: vaginal bleeding duration, **gestational sac size**, **myometrium thickness**, presence of **uterine arteriovenous fistula**, **placenta accreta**, **ultrasound blood flow grading**. Lab: **Hb**, **β-hCG**.	NB, MLP, DT, KNN, LR, RF, SVM, XGB	Hold-out Training: 80% Testing: 20%<br><br>10-fold CV<br><br>EV (n=295)	NB showed best performance (accuracy 0.879, AUC 0.882, F1 score 0.966).
Choi *et al*. (2025)[226]	Predict uterine bleeding after COVID-19 vaccination	2,296,675 vaccinated cases<br><br>(1,719 experienced bleeding)	Demographics: age, region of residence, household income, BMI. Clinical: smoking, alcohol use, physical activity, SBP, DBP, CKD, chronic liver and infectious diseases, blood diseases, musculoskeletal diseases, rhinitis, asthma, food allergy, atopy, COPD, DM, SAH, cancer, rheumatism, dyslipidemia, menstrual disorders, **medications (**steroids, immunosuppressants), **vaccination data** (type, **number of doses**, **brand**). Lab: **Hb**, **AST**, ALT, glucose, **cholesterol**, eGFR.	LR, GBM, AdaBoost, RF, XGB, AdaBoost+GBM, AdaBoost+LR, GBM+LR, AdaBoost+GBM+LR	Hold-out Training: 80% Testing: 20%<br><br>5-fold CV	**GBM+LR achieved the best performance (AUC 0.661 and balanced accuracy 0.617).**
Futterman *et al*. (2025)[227]	Predict surgical morbidity (including bleeding and transfusion) in PAS	401 cases<br><br>(309 experienced at least one morbidity)	Demographics: age, race, ethnicity, **socioeconomic status**, language, insurance. Clinical: obstetric history (prior cesareans, myomectomy, **hysterectomy**, ART use), prenatal care and imaging (**timing of PAS suspicion**, first-trimester ultrasound, **prenatal MRI**, **PAS grade**), delivery details (**gestational age at first visit** and delivery, delivery timing, scheduled vs. unscheduled surgery), maternal health (**prepregnancy and delivery BMI**, comorbidities), care coordination (**number of prenatal visits**, multidisciplinary team involvement).	GBT	5-fold CV	GBT achieved **moderate accuracy bleeding and transfusion prediction (AUC =0.75/0.70).**
Guedalia *et al*. (2022)[228]	Predict neonatal subgaleal hemorrhage	35,552 deliveries<br><br>(109 developed hemorrhage)	Maternal data: **age**, height, **parity**, **gravidity**, smoking, **prior cesarian section**, previous abortions, previous ectopic pregnancies, blood type. Index pregnancy features: pre-pregnancy maternal body weight, current maternal weight, need for assisted reproductive	BRF, CatBoost, AdaBoost	3-fold CV	**BRF preformed best (AUC 0.88), outperforming traditional risk stratification methods.**

			technology, GBS status, estimated fetal weight, gestational age at labor. Intrapartum data: anesthesia type, initiation of labor, presenting part, induction of labor, amniotic fluid, rupture of membranes.			
Holcroft *et al*. (2024)[229]	Predict PPH	430 patients (108 with PPH)	Demographics: **age**, BMI, medical insurance status. Clinical: multiparity, multiple pregnancy, previous postpartum hemorrhage, pre-labour hemorrhage, intrauterine fetal death. Lab: **Hb level during labour**.	LR, ENLR, RF, ERT, XGB	Hold-out Training: 60% Testing: 10% Validation: 30% 5-fold CV	RF performed best (sensitivity 80.7%, specificity 71.3%, misclassification rate 12.19%).
Krishnamoorthy *et al*. (2022)[230]	Predict PPH	11,000 patients (1,042 experienced PPH)	The dataset included 149 features, but the specific variables used to train the model were not individually stated.	RF, XGB, GBDT, SVM, EL-HC, EL-SC, OBCSA-OSAE	10-fold CV	OBCSA-OSAE outperformed all ML and Ensemble models, achieving the highest AUC (0.983), accuracy (94.8%), precision (98.3%), and F1-score (96.5%).
Lengerich *et al*. (2024)[231]	Predict PPH at admission	85,766 births (641 with PPH)	Demographics: age, race, ethnicity. Clinical: BMI, height, smoking, **IVF**, **parity**, DM, hypertensive disorders, mental illness, cholestasis, prior stillbirth, polyhydramnios, **preeclampsia**. Lab: **HCT**, PLT. Others: gestational week at birth, admission hour/day/month, **cervix dilatation**, birthweight, **retained placentae**, length of 1st stage. Procedure: **aspirin**, **oxytocin**, **induction of labor**, **cervical priming**, **cesarean delivery**, **forceps/vacuum**, artificial rupture of membranes, epidural use, episiotomy.	GAM	Hold-out Training: 68% Testing: 32% Bootstrap resampling EV (n=27,743)	GAM achieved an AUC of 0.67, outperforming the CMQCC screen (AUC 0.52).
Liu *et al*. (2022)[232]	Predict PPH after vaginal delivery	10,520 births (854 with PPH)	Demographics: age. Clinical: **BMI**, **gestational hypertension**, **neonatal weight**, **duration of second-stage labor**, **SI**. Lab: **HCT**, **WBC**. Uterine Contraction Features: **contraction frequency**, contraction intensity, **mean contraction area**, **total amniotic fluid volume**.	LR, RF, KNN, LGB, LGB+LR	Hold-out Training: 90% Testing: 10%	LGB+LR performed best (AUC 0.803, sensitivity 0.694, specificity 0.800).

Mehrnoush *et al.* (2023)[233]	Predict PPH	8,888 deliveries<br><br>(163 with PPH)	Demographics: **age, nationality, education level, living place, prenatal care adequacy. Clinical: smoking status, anemia severity, cardiovascular disease, SAH, hepatitis, pyelonephritis, COVID-19, DM, thyroid dysfunction; gestational age, parity, onset of labor, preeclampsia, eclampsia, abnormal placentation, placental abruption, chorioamnionitis, meconium-stained fluid, fetal presentation, method of delivery, perineal lacerations, shoulder dystocia, fetal macrosomia, type of anesthesia during cesarean delivery.**	XGB, LGBM, RF, DT, LR, LinearR, SVM, KNN-based permutation methods, Feedforward DL	Hold-out<br>Training: 80%<br>Testing: 30%	XGB achieved the highest performance: AUC 0.98, accuracy 0.98.
Meyer *et al.* (2022)[234]	Predict PPH using EEHRs	5,261 deliveries<br><br>(1,321 with PPH)	Demographics: age, race, ethnicity, marital status, insurance status, years of education. Clinical: **pre-pregnancy (SAH**, CKD, heart disease, seizure, prior cesarean, and pre-pregnancy BMI and weight), **pregnancy-related conditions** (anemia, DM, SAH, preeclampsia, placenta previa, accreta, abruption, intrauterine growth restriction, polyhydramnios, multiple gestation, fetal macrosomia or demise, breech presentation, antepartum bleeding, hospitalizations, premature rupture of membranes), tobacco, alcohol, illicit drug use, **clinical at admission** (SBP, DBP, temperature, weight, gestational age, presence of spontaneous or trial labor), medications (Mg sulfate, steroids). Lab: Group B Streptococcus colonization, Hb.	LR, LASSOR, RF, GBM	10-fold-CV<br><br>(this study was an EV)	RF performed best (AUC 0.64)
Shazly *et al.* (2024)[235]	Predict clinical outcomes in placenta accreta, including massive blood loss	727 patients<br><br>(≈ 129 experienced massive blood loss)	Demographics: age, BMI, **ethnicity**. Clinical: smoking, **parity**, **previous cesarean sections**, IVF pregnancy, type of conception, obstetric complications, previous gynecological surgery, gestational age at diagnosis and delivery, method of diagnosis, intrapartum diagnosis, **placental location**, type of invasion, organ invasion, type of uterine	LR	Hold-out<br>Testing: 80%<br>Training: 20%	LR accurately predict massive blood loss (AUC 0.86).

			incision, relation of incision to placenta, antenatal steroids, indication of delivery, preoperative and intraoperative US, PAS type. Procedure: PAS management, outcome and type of conservative treatment, interventional radiology, intraoperative surgical complications, cord clamping, tranexamic acid use, blood loss, DIC, transfusion, IUFD. Others: admission to ICU, LOS, postoperative morbidity and mortality. Laboratory: **Hb**.			
Susanu *et al*. (2024)[236]	Predict Intra- and PPH	203 patients<br><br>(68 experienced PPH)	Demographics: maternal age, residential environment. Clinical: **preeclampsia**, **SAH**, renal disease, SLE, antiphospholipid syndrome, **obstetric characteristics** (parity, **macrosomia**, **obesity**, **placental abnormalities**, **labor arrest**, fetal demise, **instrumental delivery**, **vulvovaginal lacerations**).	NB, DT, RF, SVM	Hold-out<br>Training: 80%<br>Testing: 20%	NB outperformed all models achieving 98.6% accuracy, 96.3% sensitivity, and a 3.7% false negative rate.
Walczak *et al*. (2021)[237]	Predict perioperative bleeding and transfusion in myomectomy patients	96 patients<br><br>(Not explicitly stated for bleeding)	**Demographics:** age, BMI. Clinical: **number of fibroids**, **type** and location of **fibroids**, largest fibroid diameter, **anemia**. Lab: **Hb**, HCT, **PLT**.	RBF-ANN, Backpropagation-ANN, Ensemble ANN, LR, LinearR	2-fold CV	ANN predicted **bleeding within 100 ml of actual loss in 58.3% of cases, significantly outperforming regression (38.5%).**
Wang *et al*. (2024)[238]	Predict the volume of bleeding during cesarian section	5,468 patients<br><br>(~695 had blood loss ≥ 1,000 mL)	Demographics: age. Clinical: height, weight, gestational week, SBP, DBP, hypothyroidism, infant weight, gestational DM, preeclampsia, placentae abruption, hysteromyoma, chorioamnionitis, number of	LR, LinearR, GB, XGB, MLP, RF	Hold-out<br>Training: 80%<br>Testing: 20%	**RF** was the top performer (MAE 21.7 mL; RMSE 33.75 mL).

			pregnancies, intrapartum fever, other prenatal symptoms, anesthesia (spinal-epidural, epidural, general, spinal, other), ASA. Lab: **Hb**, **WBC**, **PLT**, **Na**, **K, Ca**, PT, INR, APTT, TT, FIB.			
Wang *et al*. (2024)[239]	Predict PPH in vaginal and caesarean deliveries	768 deliveries<br><br>(337 experienced PPH)	Demographics: **age**. Clinical: gravidity, **parity**, **gestational week**, newborn weight, **multiple pregnancy**, **assisted reproduction**, uterine fibroids, **preeclampsia**, uterine scar, **preterm birth**, **placenta previa**, ovarian cyst, chorioamnionitis, perineal laceration, **DM**, caesarean section.	RF, AdaBoost, Gaussian NB, GB, HGB, MLP, LR	Hold-out<br>Training: 80%<br>Testing: 20%	RF performed best for caesarean cases (AUC 0.95); AdaBoost led for vaginal births (AUC 0.76).
Westcott *et al*. (2022)[240]	Predict maternal PPH	30,867 patients<br><br>(2,179 with PPH)	Demographics: age, **BMI**. Clinical: gestational age, SBP, DBP, SpO_2, temperature, live birth count, baseline fetal HR, amniotic fluid color, **cesarean delivery prior to labor or rupture**, **cesarean delivery scheduling status**, mode of delivery. Lab: **HCT**, Hb, **PLT**, RBC, WBC, EOS, LYM, LYM%, MCH, MCHC, MCV, MPV, MO%, NEU, NEU%.	LR, RF, SVM, GBDT	Hold-out<br>Training: 70%<br>Testing: 15%<br>Validation: 15%	GBDT performed best (AUC 0.979, accuracy 98.1%, sensitivity 76.3%).
Zheng *et al*. (2024)[241]	Predict intraoperative blood loss during cesarean sections	346 patients<br><br>(150 experienced bleeding)	Demographics: **maternal age**, **gestational age**. Clinical: history of **uterine surgeries**, **PAS**. Lab: **PT**, APTT, FIB, TT. **Radiomics-derived features.**	SVM integrating clinical, radiomic, and coagulation data in five combinations: CFI, C-CFI, R-CFI, C-R, and C-R-CFI	Hold-out<br>Training: 70%<br>Testing: 30%<br><br>EV (n=122)	C-R-CFI model performed best with AUCs of 0.873 (internal) and 0.806 (external).
Zheutlin *et al*. (2022)[242]	Predict maternal PPH using EMR	70,948 deliveries	Demographics: age, **race**, ethnicity, BMI, weight, insurance. Clinical: **SBP**,	LGBM	Hold-out<br>Training: 80%	LGBM outperformed clinical tools (AUC 0.71),

		(6,639 experienced PPH)	DBP, **pulse**, temperature, pregnancy and delivery characteristics (**gestational age**, labor induction, labor trial, **time from admission to delivery**, **delivery method**), medical history (**prior PPH**, preterm birth, **anemia**, assisted reproductive technology, tobacco use), diagnoses (**preeclampsia**, placenta disorders, uterine rupture, anemia types, infections, fetal growth issues, fluid/electrolyte disorders), medications (**oxytocin**, magnesium sulfate, betamethasone, antibiotics, bupivacaine). Procedure: dilation and curettage, hysteroscopy, laparotomy, myomectomy, pelvic exams). Lab: Hb, HCT, **RBC**, **MCH**, **RDW**, **PLT**, **WBC**, **NEU**, BASO, MO.		Testing: 20%<br><br>100-fold CV	with 28% PPH in high-risk vs. 15–19% for standard tools.
**Oncology**						
Grdinic *et al*. (2024)[243]	Predict bleeding in cancer-associated thrombosis on anticoagulation	1,080 patients<br><br>(major or clinically relevant bleeding: 83 (1–90 days), 122 (1–365 days), 51 (90–455 days))	**Demographics: age, sex, height, weight. Clinical: smoking, HR, type and location of VTE, history of bleeding, cancer type and stage, comorbidities (GI bleeding history, renal failure, COPD), treatment-related variables (anticoagulant type/duration, time of bleeding relative to VTE diagnosis, concomitant medications), use of CT scan, US, and scintigraphy to confirm VTE. Lab: DD, creatinine, cholesterol, amylase, ALB, CEA, ALP, ALT, APTT, INR, FIB, WBC,**	LASSOR, RidgeR, RF, XGB	10-fold CV	XGB and LASSOR outperformed CAT-BLEED (AUCs up to 0.65 vs. 0.47).

			**Retic, MCV, Hb, HCT, PLT, ProBNP, GGT, bilirubin, thrombophilia tests, SpO_2.**			
Martín *et al*. (2024)[244]	Predict major bleeding in anticoagulated cancer patients with VTE	1,816 patients<br><br>(895 experienced major bleeding)	**Demographics: age. Clinical: presence of metastases. Lab: Hb, PLT, WBC, creatinine.**	LR, DT, RF	Hold-out<br>Training: 75%<br>Testing: 25%	ML models (RF best with AUC 0.61) outperformed CAT-BLEED (AUC 0.53).
Truong *et al*. (2024)[245]	Predict major bleeding events in cancer and AF	18,388 patients<br><br>(221 experienced major bleeding)	**Demographics:** age, sex, race/ethnicity, calendar year, region, urbanicity, **household income**, **education level**, Medicaid eligibility. Clinical: SAH, CHF, DM, **prior stroke/TIA or bleeding,** vascular disease, liver or **renal disease**, alcohol use, asthma, COPD, hematological disorders, dementia, depression, thrombocytopenia, peptic ulcer disease, **cancer characteristics** (**type**, stage, grade, **time from diagnosis to AF onset**, active status, treatments: radiation, surgery, **antineoplastic agents**), **medications (ACE inhibitors, ARBs**, antiplatelets, NSAIDs, beta blockers, antiarrhythmics, diuretics, proton pump inhibitors, SSRIs, statins).	EN, RF, XGB; SVM, NN	Hold-out<br>Training: 70%<br>Testing: 30%<br><br>10-fold CV	Major bleeding prediction showed low performance across all models (best AUC = 0.623 with RF), but ML still outperformed HAS-BLED (AUC = 0.574).
**Orthopedics**						
Shohat *et al*. (2023)[67]	Predict major bleeding after total joint arthroplasty	35,963 patients<br><br>(293 experienced major bleeding)	Demographics: **age**, gender, race, BMI. Clinical: ASA, **smoking**, alcohol, history of VTE, hormone replacement therapy, hyper-coagulopathy, AIDS, MI, HF, other cardiovascular disease, AF, COPD, DM, dementia,	XGB, RF, LASSO, SVM	Hold-out<br>Training: 70%<br>Testing: 30%<br><br>5-fold CV	LASSO was best for major bleeding (AUC 0.803).

			hemiparesis, liver disease, **peptic ulcer disease**, **PVD**, CKD, inflammatory arthritis, cerebrovascular accident, sleep apnea, malignancy. Surgical procedure: **surgery type**, **revision status**, **operative time**, **anesthesia type**, use of cement, TXA, **blood transfusion**, **surgical approach**, **postoperative anticoagulation therapy**.			
**Trauma**						
Fernández *et al*. (2023)[246]	Predict massive hemorrhage in severe trauma	473 patients<br><br>(≈ **37 developed massive hemorrhage)**	**Demographics:** age, sex. Clinical: **HR**, SBP, DBP, **GCS**, **SI**, **prehospital fluid therapy volume**. **Injury Characteristics: suspected femur fracture or unstable pelvic fracture**, **penetrating trauma**.	RF, SVM, GBM, NN	Hold-out<br>Testing: 80%<br>Training: 20%<br><br>10-fold CV	RF performed best (AUC 0.993); all ML models outperformed traditional scores for massive hemorrhage prediction.
Guo *et al*. (2022)[247]	Predict massive hemorrhage in trauma	2,353 patients<br><br>(377 with massive hemorrhage)	**Demographics: age, sex. Clinical: HR, RR, pulse pressure, body temperature, SpO_2, clinical symptoms (oliguria /anuria, unconsciousness). Lab:** pH, PO_2, $PaCO_2$, **BE**, lactate, **Hb**, MCV, MCH, MCHC, RDW, PLT, WBC, differential counts, FIB, PT, APTT, TT, INR, DD, ALT, AST, ALP, GGT, bil, ALB, creatinine, BUN, uric acid, Na, K, Cl, P, Mg, glucose, amylase, lipase, LDH, troponin T, Pro-BNP. Imaging: **displaced pelvic fracture**, **positive CT/FAST scan for hemorrhage**.	LASSO+LR	10-fold CV	LASSO + LR achieved AUC 0.894, outperforming vital signs (AUC 0.718), TASH (0.860), and PWH (0.851).

Lee *et al*. (2022)[248]	Predict hemorrhage in trauma ICU patients	2218 patients<br><br>(1036 experienced bleeding)	Demographics: age, gender. Clinical: **ISS**, NISS, GCS, **HR**, SBP, DBP, RR, temperature, **SI**, **transarterial embolization**. Lab: **Hb**, **HCT**, MCV, PLT, WBC, BUN, creatinine, PT, APTT, INR, FIB, Na, Ca, K, Cl, Mg, lactate, amylase, lipase, Tbil, ALK, AST, ALT, glucose, ALB, procalcitonin, ABG (pH, $PaCO_2$, PaO_2, SpO_2, BE, bicarbonate).	LMT, RF	10-fold CV	LMT achieved the best predictive performance (AUC 0.816).
Li *et al*. (2020)[249]	Predict traumatic coagulopathy	1,385 patients<br><br>(71 had an INR>1.5)	Clinical: **SI**, **DBP**. Lab: **RBC**, **BE**, **pH**, **lactate**.	RF, LR	Hold-out<br>Training: 70%<br>Testing: 30%<br><br>EV (n=587)	LR had a higher AUC (0.858 vs. 0.830), suggesting better discrimination, while RF showed better overall accuracy and classification performance.
Perkins *et al*. (2020)[250]	Predict the risk of trauma-induced coagulopathy in injured patients	973 patients<br><br>(110 developed coagulopathy)	Clinical: **HR**, **SBP**, temperature, RR, mechanism of injury, GCS, hemothorax, FAST, long bone fractures, unstable pelvic fractures. Lab: **lactate**, **BD**, **pH**. Others: volume of pre-hospital fluid administered and energy of trauma (high/low velocity).	BN	Hold-out<br>Training: 62%<br>Testing: 38%<br>(Test set: different period<br><br>10-fold CV<br><br>EV (n=118)	BN showed excellent performance (AUC 0.93 development; 0.95 combined validation).
Richards *et al*. (2025)[251]	Predict acute traumatic coagulopathy using a ML-based CRI	17,567 patients<br><br>(control group not explicitly defined)	Clinical: **HR**, **MAP**, **SpO_2**, **SI**, waveform-derived **HR variability features** (EKG and photoplethysmography).	XGBT	Hold-out<br>Training: 50%<br>Testing: 50%<br>(Test set: different period)	CRI predicted ATC with high accuracy (AUROC ~0.97), high NPV (1.00), but low PPV.

Yang *et al*. (2021)[252]	Predict coagulopathy in traumatic brain injury	999 patients<br><br>(493 with coagulopathy)	Clinical: SOFA, CHF, MI, blood transfusion. Lab: INR, PT, APTT, PLT, HCT, RBC, Hb, RDW, BUN, creatinine, Na.	ANN, NB, GBM, AdaBoost, RF, BT, XGB	10-fold CV<br><br>EV (n=697)	AdaBoost performed best in EV (AUC 0.924)
Xiong *et al*. (2025)[253]	Predict pre- and postoperative traumatic coagulopathy	13,237 patients<br><br>(6,566 with traumatic coagulopathy)	Demographics: age, sex, race, weight, BMI. Clinical: **HR**, **SBP**, DBP, shock status, SAH, DM, CVD, COPD, chronic bronchitis, asthma, CKD. Surgical procedure: **emergency status**, surgery type, **perioperative transfusion**. Lab: **Hb**, **HCT**, **RBC**, WBC, PLT, MPV, RDW, MCV, MCHC, **APTT**, **PT**, **INR**, DD, **AST**, **ALT**, AKP, GGT, TP, ALB, GLO, urea, creatinine, uric acid, **Na**, K, **Ca**.	RF, GB, XGB, DT, LR, SVM, NN, NB, AdaBoost, KNN	Hold-out<br>Training: 70%<br>Testing: 30%<br><br>5-fold CV<br><br>EV (n=3212)	RF performed best (AUC 0.91, AUPRC 0.89, accuracy 0.84, sensitivity 0.80, specificity 0.88, and F1-score 0.84).
**Urology**						
Meng *et al*. (2024)[254]	Predict postoperative bleeding after percutaneous nephrolithotomy	290 patients<br><br>(35 experience bleeding)	Demographics: age, gender, **BMI**. Clinical: SAH, DM, CHD, lung diseases, brain diseases, spinal deformity, lesion side, **stone size** and location, **multiple stones**. Surgical procedure: puncture site, channel type and number, operation time, intraoperative blood loss, **intraoperative SBP and DBP**, **preoperative HR**, mode of anesthesia, stone co-infection.	LR, RF, XGB	Hold-out<br>Training: 80%<br>Testing: 20%	RF performed best (AUC 0.679, accuracy 74.5%, F1-score 0.79).
**Multiple Specialities**						
Laursen *et al*. (2023)[255]	Improve medical doctors' detection of hemorrhage events in EHRs	25,862 sentences<br><br>(NA)	Unstructured clinical text from EHRs — 25,862 sentences labeled as positive or negative for hemorrhage presence, with additional classification by anatomical location (12 categories).	LR, GRU+CNN, ELECTRA	Hold-out<br>Training: 80%<br>Testing: 10%<br>Validation: 10%	ELECTRA performed best (**93.7% sensitivity, 98.1% specificity**). Medical Doctors identified 33-48% more

						hemorrhages with AI assistance.
Mitra *et al*. (2023)[256]	Detect bleeding events in EHRs	1,079 HER notes<br><br>(NA)	Free-text EHR notes with labeled entities, including **bleeding events**, anatomical sites, **severity**, **medications**, **lab evaluations**, and suspected alternative causes.	CRF, LM-LSTM-CRF, BERT-based models	Hold-out<br>Training: 79%<br>Testing: 21%<br><br>Partial CV	LM-LSTM-CRF performed best, achieving the highest F1 score (0.75). Models struggled with recognizing complex entities, such as alternative causes of bleeding.
Mittman *et al*. (2024)[257]	Predict major bleeding risk at admission in medical patients	46,314 patients<br><br>(268 experienced major bleeding)	**Demographics:** age, sex, race. Clinical: smoking, alcohol use, **prior bleeding**, rheumatic disease, **peptic ulcer**, TIA, stroke, SAH, **HF**, pericarditis, DM retinopathy, **sepsis**, ICU admission, active cancer, peptic ulcer, HF, medications (antiplatelet drugs, **steroid**, NSAIDs, SSRI). Others: presence of a CVC, recent hospital discharge. Lab: PLT, INR, PTT, eGFR.	LASSO	Hold-out<br>Training: 70%<br>Testing: 30%<br><br>10-fold CV	LASSO outperformed IMPROVE for predicting major bleeding, with AUC 0.85 vs. 0.70, sensitivity 74%, specificity 86%, and Brier score 0.0057.
Nwanosike *et al*. (2024)[258]	Predict bleeding in DOAC users across different BMI categories	97,413 patients<br><br>(811 experienced bleeding)	**Demographics: age**, sex, ethnicity, BMI (**normal weight and overweight**). Clinical: **medication** (**DOAC type**, dose, treatment duration), comorbidities, **bleeding risk**, VTE risk, eGFR, **CKD**, stroke risk). E**ncounter-level** variables: length of hospital stay, number of emergency visits.	DT, RF, KNN, LR, GB, SVM	Hold-out<br>Training: 70%<br>Testing: 30%	RF achieved best overall prediction accuracy (~99.2%).
Pedersen *et al.* (2021)[259]	Detect and visualize bleeding events in EHRs	11,786 sentences from 300 annotated EHRs<br><br>(5,893 positive sentences)	**Sentence-level annotations of bleeding events** across multiple types, with **100-dimensional GloVe word embeddings** and **text preprocessing** (sentence tokenization, stopword removal, character normalization).	CNN, RNN, CNN+RNN, RuleBC	Hold-out<br>Training: 80%<br>Testing: 10%<br>Validation: 10%	CNN+RNN performed best (AUC 0.90, F1-score 0.90, accuracy 0.90, sensitivity 0.90, specificity 0.90).
Rickards *et al*. (2014)[260]	Detect hypovolemia (simulated hemorrhage)	24 patients<br><br>(NA)	Clinical: **HR**, **stroke volume**, EKG-derived features, **skin temperature**, **galvanic skin response**. Sensor data: **heat flux**, two-axis acceleration, motion-based	Type of ML model not explicitly stated	Leave-One-Out CV	ML model achieved ≥90% accuracy,

	by using low-level physiological signals		variables (toe strikes, mean crossing counts)			sensitivity, specificity, and precision
Shi *et al*. (2023)[45]	Predict intraoperative bleeding using surgical EMR data	48,543 patients<br><br>(9,728 with bleeding)	Demographics: age, sex, BMI. Clinical: pulse, kidney disease, heart disease, SAH, DM, coagulopathy. Surgical procedure: ASA, anesthesia type, surgery type, surgical level, emergency/elective status, **operative time**, surgeon-related (**length of employment**, academic degree, department, professional title). Lab: SBP, glucose, **DD**, Hb, **HCT**, TT, PT, APTT.	LGBM, XGB, CatBoost, AdaBoost, LR, LSTM, MLP	Hold-out<br>Training: ~66%<br>Testing: ~33%<br><br>CV performed (fold number not specified)	LGBM was the best-performing model (AUC 0.933, sensitivity 0.87, specificity 0.85, accuracy 0.87).
Yoon *et al*. (2024)[261]	Estimate intraoperative blood loss using gauze images	1,208 gauze images<br><br>(NA)	Masked gauze images, **patch-wise crumpled state features** based on local binary pattern, texture features, and image patches from surgical video frames.	EfficientNet-B5 + Fully Connected layers for regression; with variations: Base model, CS model, and P-W CS model	Hold-out<br>Training: 60%<br>Testing: 20%<br>Validation: 20%<br><br>5-fold CV<br><br>EV (n=473)	P-W CS performed best (MAE 0.25g and MAPE 7.26%, per gauze, and MAE 0.58g, per patient.

Table S3. Harness and Optimize physiological reserve of anemia.

Reference (Year)	PBM Application	Sample Size (case group)	Model Variables Top Predictors (bold)	Models	Model Validation	Key Findings
**Cardiology**						

Hamilton *et al*. (2023)[37]	Predict complications after PCI (including bleeding and transfusion)	107,793 procedures<br><br>(2,592 transfused; 942 experienced bleeding)	**Demographics: age, sex, race, height, weight. Clinical:** DM, HF, prior PCI, prior CABG, CVD, PAD, lung disease, smoking, **frailty**, **LVEF**, clinical status, cardiovascular instability, ventricular support, cardiac arrest, stress test results, PCI indication, Cath lab indication. Lab: **Hb**, total cholesterol, HDL, **creatinine**.	XGB, LR	Hold-out Training: 60% Testing: 40%<br><br>3-fold CV<br><br>EV (n=56,583)	XGB had excellent discrimination: AUC 0.917 (transfusion) and 0.887 (bleeding); outperforming LR for all outcomes in internal and EV.
**Cardiothoracic**						
Che *et al*. (2024)[262]	Predict intraoperative blood transfusion in aortic surgery	3,654 patients<br><br>(NA)	Demographics: age, **gender**, BMI. Clinical: **weight**. Lab: **Hb**, **RBC**, **DD**, INR, ALB, residual alkali, pH, PaO_2, CK, LDH, WBC, Cl. Surgical procedure: **estimated operation time**, blood volume, **deep hypothermia circulatory arrest**, **thoracoabdominal aorta replacement**.	ETR, CatBoost, RF, XGB, BR, RidgeR, LinearR, EN, LASSOR, HuberR, KNN, SVM	Hold-out Training: 70% Testing: 30%	ERT performed best (R^2 = 0.50, MAE = 1.17 units of blood transfused).
Chen *et al*. (2024)[263]	Predict intraoperative blood transfusion in aortic valve replacement surgery	702 patients<br><br>**(269 transfused)**	Demographics: **gender**, **age**. Clinical: **weight**, height, cardiac function. Lab: **Hb**, **HCT**, RDW, **MCH**, **TP**, **FIB**, LDL, globin, creatinine, uric acid, TBil, IBil, DBil. Surgical procedure: **type of operation** (surgical AVR or transcatheter AVR).	LR, GB, CatBoost, ET, GNB, MLP, DT, AdaBosst, RF, XGB, LGBM, BNB, SVC	Hold-out Training: 70% Testing: 30%<br><br>5-fold CV	LR performed best (AUC 0.872 and accuracy 81%).
Cunha *et al*. (2024)[264]	Predict blood transfusions in cardiac surgery	495 patients<br><br>(284 transfused)	**Demographics: age**, **sex**, **BSA**. Clinical: SAH, DM, **prior cardiac surgery**. Surgical procedure: **CPB**, urgency of procedure, surgery type. Lab: **Hb**, HCT, creatinine.	LR, MLP, RF, SVM	Hold-out Training: 80% Testing: 20%<br><br>Stratified k-fold CV	LR achieved the best AUC (0.735); all ML models outperformed TRACK (0.676) and TRUST (0.662) scores.
Hur *et al*. (2024)[265]	Predict RBC demand in thoracic surgery	7,843 surgeries<br><br>(NA)	Demographics: age, sex. Clinical: **MI**, CHF, peptic ulcer, liver disease, DM, hemiplegia, **renal disease**, **malignancy**, metastatic disease, AIDS, CCI. Surgical procedure: surgery type, anticoagulant	GPR, RF, XGB, ANN, LinearR	Hold-out Training: 79% Testing: 21%<br><br>5-fold CV	XGB performed best (RMSE=3.203, adjusted R^2=0.399). It predicted RBC needs more accurately than MSBOS while

			use, **MSBOS**. Lab: **Hb**, **PT/INR**, **APTT**, **PLT**.			avoiding ~200 excess RBC units.
Li *et al*. (2024)[266]	Predict perioperative RBC transfusion in CPB	2,420 patients<br><br>(518 **transfused)**	**Demographics: age**, **sex**, **BMI**. Clinical: temperature, HR, pulse pressure, DM, MI, PVD, hyperlipidemia, SAH, COPD, CKD, endocarditis, carotid artery stenosis, stroke. Surgical procedure: valvular, **CABG**, **congenital**, transfusion volume. Lab: **Hb**, PLT, **WBC**, AST, ALT, **ALP**, **Tbil**, creatinine, **BUN**, ALB.	CatBoost, RF, SVM, XGB, LGBM, GB, ET, DT, KNN, NB, LR, AdaBoost	Hold-out Training: 80% Testing: 20%<br><br>5-fold CV<br><br>EV (n=8,781)	**CatBoost and RF outperformed other models in EV** (AUC> **0.74).**
Shen *et al*. (2025)[267]	Predict postoperative blood transfusion in cardiac surgery	6,502 surgeries<br><br>(2,019 transfused)	**Demographics: age**, sex, race, height, **weight**. Clinical: SBP, DBP, SpO_2, **pulse**, ASA physical and emergency status, SAH, CAD, MI, CHF, AF, pacemaker, stroke or TIA, PAD, DVT or PE, DM, insulin use, CKD, dialysis, pulmonary hypertension, COPD, asthma, obstructive sleep apnea, cirrhosis, cancer, gastro-esophageal reflux, dementia, anemia, smoking. S**urgical details** captured through CBOW-transformed procedure notes Lab: **HCT, Hb, RDW, PLT, RBC, WBC, Coombs test+,** creatinine, **BUN**, **glucose**, Cl, Ca, NT-proBNP, APTT.	LR, RF, GBM, XGB, DNN, Multi-task DNN, VAE, Beta TC VAE, Factor VAE, cVAE, PN, MAML, surgVAE	5-fold CV	surgVAE performed best (AUC 0.874 and AUPRC 0.737).
Sun *et al*. (2024)[268]	Predict blood transfusion in MIDCAB surgery	766 patients<br><br>(107 transfused)	Demographics: age, gender, **BMI**, **weight**. Clinical: blood volume, RR, **HR**, temperature, profuse sweating, tearing sharp pain, limitation of physical activity, MI, pulmonary tuberculosis, pleural thickening, hyperlipidemia, NYHA, **PCI**, surgical approach, number of grafts, medications (aspirin, clopidogrel). Lab: CK, CK-MB, α-HBDH, hs-cTnT, NT-proBNP, **Hb**, HCT, RBC, **PLT**, WBC,	XGB, GB, ET, LR, LDA, CatBoost, AdaBosst, RF, DT, KNN	Hold-out Training: 80% Testing: 20%	XGB achieved the best performance (AUC 0.726, accuracy 0.854).

			cholesterol, triglycerides, HDL, LDL, ApoA1, ApoB, lipoprotein, **PT**, APTT, FIB, INR, CRP, AST, ALT, **creatinine**, eGFR.			
Tschoellitsch *et al*. (2022)[269]	Predict perioperative MT in cardiac surgery	3,782 patients<br><br>(139 received MT)	Demographics: **age**, gender. Clinical: **EuroScore II**, **ASA**, NYHA, **urgency of surgery**, **surgical type**, arteriopathy, DM, PH, endocarditis, renal impairment, MI, LVEF. Lab: **Hb**, PLT, APTT, WBC, MCV, **creatinine**, **eGFR**, CRP.	LR, RF, NN, GBM, AdaBoost	Hold-out<br>Training: 80%<br>Testing: 20%<br><br>5-fold CV	RF and GBM achieved the best performance (AUC 0.81).
Wang *et al*. (2022)[270]	Predict intraoperative RBC transfusion in cardiothoracic surgery	2,847 patients<br><br>(173 received ≥4 RBC units)	Demographic: age, gender, ethnicity. Clinical: SAH, DM, CKD, PAD, CVD. Lab: **Hb**, PLT, creatinine, **Ca**, **K**, **ALB**, **barometric pressure**, pH, $PaCO_2$, PaO_2, O_2Hb, bicarbonate, AG, LDH, glucose, Na, Cl, bil, AST, ALT, INR, PT, APTT, FIB, iron, urinalysis. Procedure: surgeon and anesthesiologist, CABG, left ventricular assist device implantation, **thoracoabdominal aneurysm repair**, ECMO, urgency of surgery (elective, urgent, emergent), use of cell salvage, **prolonged mechanical ventilation**. Biling/procedure codes (**CPT/ICD for ECMO**, aneurysm repair, ventilation, transplants).	RF, DT, XGB, NN, GaussianP (classification + regression)	Hold-out<br>Training: 85%<br>Testing: 15%<br><br>5-fold CV	GaussianP performed best with an AUC of 0.826 for predicting RBC transfusions of ≥4 units.
Zhou *et al*. (2024)[271]	Predict intraoperative RBC transfusion during valve replacement	423 patients<br><br>(102 transfused)	Demographics: age, sex, BMI, blood type. Clinical: smoking, drinking, NYHA, SAH, DM, stroke, AF, LVEF. Surgical procedure: surgery type. Lab: **Hb**, **HCT**, **ALT**, Tbil, **FIB**, **ferritin**, PLT, APTT, PT, TT, **folic acid**, **TAST**, **Vit B12**, **ALB**, **HbA1c**, creatinine, eGFR.	CatBoost, RF, XGB, DT, SVC, LR	Hold-out<br>Training: 80%<br>Testing: 20%	CatBoost achieved the best performance (AUC 0.752, F1 score 0.695).
**Critical Care Medicine**						

Lee *et al*. (2022)[49]	Predict RBC transfusion in major internal medical conditions	16,222 patients<br><br>(5,818 transfused)	Demographics: age, sex, race. Clinical: SBP, DBP, HR, RR, temperature, SpO_2. Lab: **Hb**, **HCT**, **creatinine**, Cl.	LGBM, XGB, RF, LSTM	Not explicitly stated	LGBM performed best (AUC **0.907,** F1-score **0.817).**
Levi *et al*. (2021)[50]	Predict transfusion needs in ICU patients with GI bleeding	4,314 patients<br><br>(2,077 transfused)	Demographics: age, gender. Clinical: temperature, HR, MBP, SAH, SBP, DBP, RR. Lab: **HCT**, **Hb**, WBC, PLT, K, ALB, BUN, creatinine, bicarbonate, APTT, glucose. Others: transfusion history, **amount of blood transfused**.	SVM, LR, RF, MLP, Ensemble model	Cross-dataset<br><br>Training: 75%<br>Testing: 25%<br><br>10-fold CV<br><br>EV (n=10,306)	Ensemble model (LR+RF) achieved the best overall performance (AUC 0.81 and sensitivity 0.93 in EV).
Sheikhalishahi *et al*. (2024)[272]	Predict blood transfusion in ICU after various surgeries	9,118 patients<br><br>(2,064 transfused)	Demographics: age, sex, height, weight. Clinical: **HR**, RR, temperature, SBP, DBP, **MAP**, NISBP, NIDBP, NIMBP, mechanical ventilation status. Lab: **Hb**, **HCT**, **RBC**, WBC, **PLT**, BE, **lactate**, bicarbonate, Na, K, creatinine, urea, **glucose**, Ca, **pH**, **SpO_2**, PaO_2.	XGB, LR	5-fold CV	XGB performed best (AUC 0.85).
Shung *et al*. (2021)[273]	Predict RBC transfusions in ICU patients with GI bleeding	2,524 patients<br><br>(2,438 transfused)	Demographics: age, gender. Clinical: **HR**, **SBP**, DBP. Lab: **Hb**, HCT, PLT, MCH, MCHC, MCV, RDW, WBC, NEU, LYM, MO, EOS, BASO, bands, ALB, Tbil, Dbil, AST, ALT, ALP, amylase, lipase, LDH, Na, K, Cl, Bicarbonate, Ca, Mg, P, AG, creatinine, **BUN**, glucose, lactate, ferritin, iron, UIBC, transferrin, TSH, troponin T, CK, CK-MB, vancomycin, BE, pH, PaO_2, $PaCO_2$, $SatO_2$, FIB, PT, APTT, INR, urine (creatinine, Na, specific gravity).	LSTM, LR, LREN	Hold-out<br>Training: 80%<br>Testing: 20%<br><br>5-fold CV<br><br>EV (n=1,526)	LSTM performed best, achieving an AUC of 0.81 (internal) and 0.65 (external).
**General Surgery**						

Chen *et al*. (2022)[274]	Predict intraoperative MT in liver transplantation	1,239 patients<br><br>(139 transfused)	Demographics: **age**, sex, weight. Clinical: clinical diagnosis (cirrhosis, liver malignant tumor, liver failure, alcoholic hepatitis, viral hepatitis, cholestatic liver disease, others), portal hypertension, ascites, hepatic encephalopathy. Lab: **Hb**, HCT, WBC, PLT, creatinine, BUN, TP, **ALB**, ALT, AST, Tbil, Dbil, **APTT**, PT, **TT**, INR, FIB, globin, uric acid.	CatBoost, LGB, XGB, GBDT, RF, AdaBoost, KNN, NB, MLP, SVM, LR	Hold-out<br>Training: 70%<br>Testing: 30%<br><br>Bootsrap (1,000 iterations)<br><br>EV (n=46)	CatBoost performed best (AUC 0.81)
Liu *et al*. (2021)[275]	Predict RBC transfusion during or after liver transplantation	1,193 patients<br><br>(864 transfused)	Demographics: **age,** gender. Clinical: weight, **clinical diagnosis** (cirrhosis, liver malignant tumor, liver failure, alcoholic hepatitis, viral hepatitis, cholestatic liver disease, others), **portal hypertension**, ascites, hepatic encephalopathy. Lab: **Hb**, WBC, PLT, creatinine, BUN, TP, ALB, **ALT**, **AST**, Tbil, **Dbil**, **APTT**, PT, INR, **globin,** uric acid.	XGB, RF, GBDT, NB, AdaBoost, KNN, SVM, MLP, LR	Hold-out<br>Training: 70%<br>Testing: 30%<br><br>5-fold CV<br><br>EV (n=31)	XGB performed best (AUC 0.813), significantly outperforming LR (AUC 0.707).
Zhang *et al*. (2022)[276]	Predict perioperative blood transfusion in total gastrectomy for gastric cancer	513 patients<br><br>(199 transfused)	Demographic: age, gender; clinical: BMI, **CONUT score**, **TyG index**, PNI; laboratorial: **Hb**; surgical: tumor size (long and short diameters), surgical technique (open surgery or laparoscopy), stapler type (circle stapler or linear stapler), **blood loss**, operation time, number of resected lymph nodes, number of positive lymph nodes; pathological: TNM stage, vascular invasion, and nerve invasion.	LR, DT, RF, LGBM, GBDT, XGB	Hold-out<br>Training: 70%<br>Testing: 30%	LR achieved the best performance (AUC 0.879) and was selected to build the final nomogram.
**Maxillofacial**						
Jalali *et al*. (2021)[277]	Predict intraoperative blood transfusion in pediatric craniofacial surgery	2,143 patients<br><br>(1,866 transfused)	Demographic: **age**, **weight**, race, ethnicity, sex. Clinical: ASA, craniosynostosis-related syndrome, type of craniosynostosis syndrome, distractor	GB, RF, SVM, EN, AdaBoost, NN	Hold-out<br>Training: 70%<br>Testing: 30%	GBM was the best-performing model, accurately predicting transfusion needs (AUC

			placement, preoperative erythropoietin use, anatomical site classification, existing tracheostomy, preoperative elevated intracranial pressure, prior craniofacial surgery. Surgical procedure: surgeon specialty (plastic, other), cell salver used, antifibrinolytic agent used, number of units transfused, number of operated sutures. Others: **surgical volume per institution**. Lab: **Hb**, **HCT**, **PLT**.		5-fold CV	0.87, F-score 0.91) and estimating blood units required (R^2 0.73).
Puladi *et al*. (2023)[278]	Predict blood transfusion in head and neck microvascular reconstruction	657 patients<br><br>(266 transfused on surgery day; 419 within 3 days)	**Demographics:** age, sex, weight, height, BSA, **blood volume**. Clinical: **ASA**, CCI, cancer. Lab: **perioperative Hb**. Surgical procedure: **flap type**, **neck dissection type**, tracheostomy, **surgery duration**.	GBM, LR, NN, RF, SVM	Bootstrapping with 500 iterations	ML models performed comparably to LR (AUC = 0.808-0.825).
Stehrer *et al*. (2019)[279]	Predict perioperative blood loss in orthognathic surgery	950 patients<br><br>(NA)	**Demographics: age, sex, BMI. Clinical: ASA. Surgical procedure: type of surgery, surgical time, type of anesthesia, calculated blood volume. Lab: Hb, HCT, RBC.**	RF	Hold-out<br>Training: 80%<br>Testing: 20%	RF achieved high predictive accuracy, with a mean prediction error of 7.4 mL and a SD of 172.3 mL.
**Neurosurgery**						
Cabrera *et al*. (2023)[280]	Predict blood transfusion following Posterior Cervical Decompression	12,913 patients<br><br>(336 transfused)	Demographics: **age**, sex, ethnicity, race, **BMI**. Clinical: weight loss, ASA, functional status, smoking history, ventilator dependence, DM, COPD, CHF, RF, dialysis, bleeding disorder, SAH requiring medication, dyspnea, preoperative transfusion, spinal pathology (surgical indication), **operative time**. Lab: **HCT**, **WBC**.	RF	10-fold CV	RF predicted transfusion with AUC = 0.902 (accuracy 75.8%, sensitivity 88.6%, specificity 75.3%).

Durand *et al*. (2018)[281]	Predict blood transfusion in adult spinal deformity	1,029 patients<br><br>(479 transfused)	Demographic: **age**, sex, **weight**, BMI. Clinical: SAH, smoking, DM, COPD, ASA, functional status, chronic steroid use, bleeding disorders, history of preoperative transfusion. Lab: **HCT**, creatinine, PLT, Na, WBC. Surgical procedure: surgical specialty, **operative duration**, **surgical invasiveness**, type of graft used, use of pelvic fixation, days from admission to operation, number of osteotomies performed.	RF, CTree	Hold-out<br>Training: 80%<br>Testing: 20%<br><br>10-fold CV	RF performed best (AUC 0.85).
Dong *et al*. (2021)[282]	Predict blood transfusion after spinal fusion for spinal tuberculosis	152 patients<br><br>(56 transfused)	Demographics: age, sex. Clinical: SAH, DM, paraplegia, **anticoagulant history**. Surgical procedure: **duration**, intraoperative **blood loss**, **number of fused vertebrae**. Lab: PT, APTT, FIB, **Hb**, HCT, MCV, MCH, **MCHC**, RDW, WBC, PLT, **ALB**, ESR, CPR.	LR, SVM, DT, MLP, NB, KNN, RF	10-fold CV	LR performed best (AUC 0.75).
Fatima *et al*. (2020)[283]	Predict postoperative complications, including transfusion, after lumbar spondylolisthesis surgery	80,610 patients<br><br>(4,268 transfused)	Demographics: **age**, **sex**, BMI. Clinical: **functional status**, **ASA**, DM, SAH, smoking, cardiovascular disease, pulmonary disease, renal disorders. Surgical procedure: **approach**, **number of levels** operated, **use of instrumentation**, use of autogenous iliac bone graft or allograft. Lab: HCT, **ALB**, **ALP**, WBC, PLT, creatinine, Na.	LR, LASSO	Hold-out<br>Training: 70%<br>Testing: 30%<br><br>10-fold CV	LR performed best (**AUC 0.70, sensitivity 98%, specificity of 95%)**.
Lang *et al*. (2023)[284]	Predict blood transfusion in lumbar posterior interbody fusion	1,905 patients<br><br>(360 transfused)	Demographic: age. Clinical: **ASA.** Surgical procedure: **posterior lumbar interbody fusion**, **levels fused**, **operative time**, **total intraoperative blood loss**, **intraoperative fluid infusion volume**. Lab: **Hb**, **HCT**.	XGB, RF, SVM, NB, ANN, LR	Hold-out<br>Training: 80%<br>Testing: 20%<br><br>10-fold CV	XGB performed best (AUC 0.958; accuracy 0.903).

Raman *et al*. (2020)[211]	Predict blood transfusion in adult spinal deformity	909 patients<br><br>(377 transfused)	**Demographics: age, sex, BMI. Clinical: ASA, MFI, DM, SAH, cardiovascular disease, COPD, osteoporosis. Surgical procedure: number of** levels fused, **pelvic fixation**, **3-column osteotomy**, **posterior column osteotomy**, **surgical duration**, Mirza Invasiveness Index, blood loss, cell salvage, TXA, RBC transfusion. Lab: **Hb**.	CIT, MLR	Not explicitly stated	CIT stratified risk with moderate adjusted R^2 values up to **0.59** for intraoperative transfusion.
Ramos *et al*. (2022)[285]	Predict perioperative blood transfusion in adult spinal deformity	1,173 patients<br><br>(580 transfused)	Demographics: **age**, sex, weight. Clinical: **ASA**, smoking, chronic steroid use, bleeding disorder, **functional status**. Surgical procedure: **surgery duration**, **pelvic fixation**, interbody graft use, **osteotomy type**, levels fused, **revision status**. Lab: **HCT**.	ANN	Hold-out<br>Training: 70%<br>Testing: 30%<br>Validation: 10% of the training set (150 epochs)	ANN achieved a good predictive performance (AUC 0.84).
Tunthanathip *et al*. (2022)[286]	Predict intraoperative transfusions in neurosurgical operations	3,021 patients	Demographics: age, **sex**, BMI. Clinical: **ASA**, **emergency operation status**, underlying diseases, anticoagulant use, **neurosurgical diagnoses**. Surgical procedure: time of operation, estimated blood loss. Lab: **Hb**, **HCT**, PLT, WBC, NLR, PT, **INR**.	NB, SVM, KNN, DT, RF, ANN	Hold-out<br>Training: 70%<br>Testing: 30%<br><br>5-fold CV	SVM and RF achieved highest AUCs (0.83 and 0.82).
Xiao *et al*. (2023)[287]	Predict intraoperative blood transfusion in aneurysm surgery	375 patients<br><br>(108 transfused)	Demographics: age, **sex**, weight. Clinical: ASA, NYHA, CVD, cardiovascular disease, renal disease, DM, **ruptured aneurysm**. Intraoperative data: blood transfusion volume, vasopressor usage. Lab: **WBC**, **Hb**, **PLT**, **DD**, PT, APTT, FIB, TP, ALB.	KNN, LR, RF, XGB	Hold-out<br>Testing: 70%<br>Training: 30%<br><br>Nested CV + 5-fold outer CV	LR performed best, showing the lowest classification error (0.2290) and an AUC of 0.799.
Xiao *et al*. (2023)[288]	Predict intraoperative blood transfusion in brain tumor resection surgery	541 patients<br><br>(141 transfused)	Demographics: **age**, gender, weight. Clinical: **ASA**, **heart function**, DM, CVD, cardiovascular disease, pulmonary disease, renal disease, liver disease,	LDA, SVM, RF, KNN, NB, XGB, NN, LR	Hold-out<br>Training: 70%<br>Testing: 30%	LR performed best (AUC 0.817), outperforming Hb alone (0.663).

			**tumor diameter**, multi-site tumor presence, history of surgery at the same time. Lab: **Hb**, **WBC**, PLT, **PT**, APTT, FIB, DD, TP, ALB, NT-proBNP.		10-fold CV	
**Obstetrics**						
Ahmadzia et *al*. (2024)[222]	Predict PPH and transfusion	185,413 patients<br><br>(10,344 transfused)	Demographics: age, ethnicity, education, marital status, insurance, BMI, gravidity, parity. Clinical: obstetric surgery (prior cesarean delivery, macrosomia, stillbirth, preterm birth, shoulder dystocia, uterine scar, STD, thyroid disease, anemia, asthma, heart disease, renal disease, seizure disorder), prenatal conditions (DM, gestational and chronic hypertension, preeclampsia, oligohydramnios, placentae previa and accreta, bleeding in 3rd trimester, cervical incompetence, fetal growth restriction, fetal macrosomia, antenatal hospital admission, chorioamnionitis, UTI, preterm birth, smoking, alcohol, recreational drug use), intrapartum (reason for admission, Bishop score, SBP, DBP, TOLAC, **mode of delivery**, **oxytocin incremental dose**, **tocolytic use**, **anesthesia type**, Mg sulfate use, **hospital type**, **presence of anesthesia nurse**, indication for labor, fetal presentation).	LR, SVM, MLP, RF, GB, TFIM, Emb	Hold-out<br>Training: 70%<br>Testing: 30%	GB performed best (AUC 0.860).
Chen et *al*. (2023)[289]	Predict intraoperative RBC transfusion in cesarean section	13,402 patients<br><br>(377 transfused)	Demographics: age, height, weight, BMI. Clinical: prior blood transfusion. Lab: Hb, **RBC**, PLT, **INR, PT**, **APTT**. Surgical procedure: **surgical method**, **surgical site**.	GBDT	Hold-out<br>Training: 80%<br>Testing: 20%	GBDT achieved 0.89 AUC, 96.85% accuracy for transfusion prediction, and 86.56% accuracy for volume prediction within 1U error.

					Simulation on 3,255 separate patients	
Lee *et al*. (2024)[290]	Predict intraoperative RBC transfusion in cesarean section	14,254 patients<br><br>(1,020 transfused)	Demographics: age, weight, **height**, BMI, parity. Clinical: PNI, GDM, **placenta previa totalis and partialis**, placenta accreta, placental abruption, **pre-eclampsia,** anesthesia type, midazolam use. Lab: WBC, **Hb**, **PLT**, **RDW**, WBC, NLR, PLR, **INR**, PLR, **PNI**, eGFR, creatinine, uric acid, ALB, AST, ALT, Tbil, Na, K, Cl.	XGB, KNN, DT, SVM, MLP, LR, RF, DNN	Hold-out Training: 60% Testing: 20% Validation: 20%<br><br>Bootstrap resampling (50x)	XGB performed best (AUC 0.826 and AUPRC 0.483).
Walczak *et al*. (2021)[237]	Predict perioperative transfusion and bleeding in myomectomy patients	96 patients<br><br>(7 transfused)	**Demographics:** age, BMI. Clinical: **number of fibroids**, **type** and location of **fibroids**, largest fibroid diameter, **anemia**. Lab: **Hb**, HCT, **PLT**.	RBF-ANN, Backpropagation-ANN, Ensemble ANN, LR, LinearR	2-fold CV	Ensemble ANN achieved 100% sensitivity but lower specificity (62.9%).
**Oncology**						
Raz *et al*. (2024)[291]	Predict RBC transfusion in cancer patients	98 patients<br><br>(49 transfused)	Demographics: age, gender. Clinical: cancer type, smoking, alcohol use, chemotherapy history, comorbidities (DM, heart disease, respiratory disease, CKD), BP, HR, LVEF, active bleeding. Lab: **Hb**, **HCT**, RDW, **PLT**, **PO_2**, **pH**, **creatinine**, **BUN**, bicarbonate, ferritin, PT, **APTT**, ALT, AST, ALP, ALB, Tbil, Dbil, CK, CK-MB, LDH, Troponin T.	LSTM, Bidirectional LSTM, MLP, AdaBoost, Bagging DT, Bagging KNN	5-fold CV	LSTM performed best (accuracy 94.2%).
Laios *et al*. (2023)[292]	Predict blood transfusion in ovarian cancer surgery	403 patients<br><br>(not explicitly stated)	Clinical: performance status, tumor grade, the size of the largest tumor bulk, disease burden metrics (Peritoneal Cancer Index and the Intraoperative Mapping of Ovarian Cancer score). Surgical procedure: timing of surgery,	XGB	Hold-out Training: 70% Testing: 30%<br><br>5-fold CV	XGB achieved a strong discriminatory performance (AUC 0.82).

			operative time, surgical complexity score, residual disease status, types of surgical sub-procedures performed. Lab: pre-treatment CA125 levels, the CA125-to-Peritoneal Cancer Index ratio, tumor histology.			
**Orthopedics**						
Buddhiraju *et al.* (2023)[293]	Predict blood transfusion after primary and revision THA	109,860 patients<br><br>(5,229 transfused)	Demographic: age, sex, race, ethnicity; clinical: ASA, **BMI**, smoking, DM, SAH, bleeding disorders, functional status, COPD, CHF, liver failure, renal failure; laboratory: **HCT**, WBC, PLT, creatinine, BUN, Na; surgical variables: **operative time** and indication.	ANN, RF, HGB, KNN, ENPLR	Hold-out<br>Training: 80%<br>Testing: 20%<br><br>5-fold CV	ANN performed best for primary THA (AUC 0.84, Brier 0.04); RF led in revision THA (AUC 0.85, Brier 0.12).
Cavazos *et al.* (2023)[294]	Predict blood transfusion in primary total knee arthroplasty	2,093 patients<br><br>(67 transfused)	Demographic: age, sex, BMI. Clinical: **ASA**, smoking, alcohol, DM, bleeding disorder, DVT/PE, **TXA**, anticoagulation, antiplatelets, steroids, opioids. Surgical: surgery type, **length of surgery**, **simultaneous bilateral surgery**. Lab: **preoperative Hb**, postoperative Hb, **preoperative creatinine**, **preoperative ALB**.	MPNN	Hold-out<br>Training: 70%<br>Testing: 30%	MPNN achieved high predictive performance (AUC 0.894, accuracy 95.8%).
Chen *et al*. (2023)[295]	Predict postoperative blood transfusion in hip fracture	1,355 patients<br><br>(210 transfused)	Demographics: **age**, gender. Clinical: previous surgery and transfusion. Lab: Pre-ALB, ALB, **globin**, TP, ABL/globin, ALT, AST, ALT/AST, Tbil, **Ibil**, PT, APTT, FIB, **Hb**, RBC, RDW, MHCH, MCV, PDW, WBC, PLT, NEU. Surgical procedure: operation time, **blood loss>200mL**, TXA, anesthesia type, surgical type, fracture type.	LR, MLP, XGB, RF, SVM	Hold-out<br>Training: 70%<br>Testing: 30%<br><br>10-fold CV	SVM (AUC 0.860) and MLP (AUC 0.858) achieved the best performance.

Chen *et al*. (2023)[296]	Predict postoperative RBC transfusion in orthopedic surgery	59,605 patients<br><br>(19,921 transfused)	Demographic: **age**, sex, **BMI**. Clinical: medications (iron, **EPO**, folic acid, VitB12), in-hospital times. Surgical procedure: **surgery type**. Lab: **ALB**, ALT, AST, creatinine, **APTT**, PT, Hb, HCT, **RBC**, MCHC, TBil, WBC.	KNN, SVM, RF, XGB, CatBoost, LR	Hold-out<br>Training: 80%<br>Testing: 20%	CatBoost performed best (AUC 0.831).
Cohen-Levy *et al.* (2022)[297]	Predict transfusion rates in primary total hip arthroplasty	7,265 patients<br><br>(703 transfused)	Demographics: age, **gender**, BMI, ethnicity, marital status, insurance status. Clinical: ASA, smoking, alcohol, drug abuse, CCI, **DM**, renal failure, depression, **bleeding disorders**. Lab: **HCT**, **PLT**. Surgical procedure: **TXA**, **anesthesia type**, surgical approach, cemented vs. non-cemented fixation, operation time.	ANN, SGB, SVM, ENP	Hold-out<br>Training: 80%<br>Testing: 20%<br><br>5-fold CV	ANN performed best (AUC 0.82).
Deng *et al*. (2024)[298]	Predict perioperative blood transfusion in total hip arthroplasty	224 patients<br><br>(63 transfused)	**Demographics: age**, gender, **BMI**. Clinical: ASA, SAH, pulmonary infection, DM, cerebral infarction, CVD, COPD, Renal failure, pulmonary hypertension, fracture. **Surgical procedure: bleeding volume**, autotransfusion, TXA, anesthesia type, colloid, **urine volume**, drainage volume, operation time. Lab: **Hb**, RBC, HCT, **PLT**, Tbil, TP, ALB, AST, ALT, creatinine, Cys-C, CLCr, CRP, PT, APTT, FIB, DD.	LR (combined with LASSOR and RF for feature selection and K-means clustering for patient subtyping)	10-fold CV	LR achieved an AUC of 0.899.
Dreizin *et al*. (2020)[299]	Predict MT and other critical interventions in pelvic fracture	253 patients<br><br>(79 received MT)	Demographics: age, sex. Clinical: injury type, **ISS**, pelvic-extremity AIS, **pelvic fracture type**, **lactate**, HR, **SBP**. Imaging: **hematoma volume**.	CNN, LR	5-fold CV	CNN improved LR prediction of the need for MBT (AUC increased from 0.74 to 0.83, $p < 0.001$).
Faure *et al*. (2025)[300]	Predict postoperative transfusion in total knee arthroplasty	774 patients<br><br>(100 transfused)	Demographics: **age**, gender, **BMI**, weight. Clinical: indication for surgery, ASA, CCI, smoking, alcohol, DM, SAH, CAD, medications (anticoagulants,	GBM	Hold-out<br>Training: 70%<br>Testing: 30%	GBM predicted transfusion with high accuracy (AUC 0.97,

			acetylsalicylic acid). Surgical procedure: operation time, tourniquet use, total blood loss, LOS, **TXA**. Lab: HCT, **Hb**, **PLT**.			sensitivity 94.4%, and specificity 85.4%).
Gowd *et al*. (2019)[301]	**Predict short-term postoperative complications (including transfusion)** in total shoulder arthroplasty	17,119 patients<br><br>(471 transfused)	Demographics: age, BMI, gender, functional status. Clinical: diagnosis (osteoarthritis, rotator cuff arthropathy, fracture, posttraumatic/dislocation, avascular necrosis, rheumatoid), smoking, DM, SAH, CHF, dialysis, ascites, cancer, dyspnea, bleeding disorder, steroid use, weight loss, ASA. Surgical procedure: operative time, anesthesia type, frailty index. Lab: **HCT**, ALB.	LR, RF, GBT, DT, KNN, NB	Hold-out<br>Training: 80%<br>Testing: 20%<br><br>CV (details not mentioned)	LR outperformed other models for predicting transfusion risk (AUC 0.77).
Guo *et al*. (2024)[302]	Predict perioperative transfusion in elderly hip fracture	805 patients<br><br>(306 transfused)	Demographics: **age**, gender. Clinical: fracture side, **fracture type**, time from injury to surgery, coronary heart disease, DM, cerebral infarction, COPD. Lab: **Hb**, WBC, NEU, PLT, glucose, AST, ALT, Tbil, Dbil, Ibil, ALB, BUN, creatinine, K, Na, Ca, PT, PTA, APTT, TT, INR, FIB.	CART, GBM, KNN, LR, NN, RF, SVM, XGB	Hold-out<br>Training: 70%<br>Testing: 30%<br><br>10-fold CV	XGB performed best (AUC 0.997).
Huang *et al*. (2021)[303]	Predict perioperative RBC transfusion is pelvic fracture	510 patients<br><br>(318 transfused)	Clinical: **MAP**, cause, type and site of fracture, **organ injury**, **hemorrhagic shock**, **time from injury to surgery**, therapy type (iron therapy, hemostasis, intraoperative cell salvage), **ISS**. Lab: **Hb**, **HCT**, PLT, creatinine, BUN, INR, **total protein**, **AST**.	XGB, RF, GBDT, KNN, NB, LR	Hold-out<br>Training: 80%<br>Testing: 20%<br><br>5-fold CV<br><br>EV (n=33)	XGB achieved the highest performance (AUC 0.99 and accuracy 95.13%), significantly outperforming other ML models and predictions based on surgeons' experience.
Huang *et al*. (2021)[304]	Predict postoperative blood transfusion in total hip and knee arthroplasty	12,642 patients<br><br>(2,367 transfused)	Demographics: **age**, **gender**, **BMI**. Clinical: **ASA**, preoperative analgesic use, anemia, DM, SAH. Surgical procedure: **operative time**, **TXA**, **drain**	LSTM, RF, DT, KNN, SVM, NB, LR	Hold-out<br>Training: 90%<br>Testing: 10%	LSTM and RF performed best (AUC 0.83 and 0.84, respectively).

			**use**, tourniquet use (knee arthroplasty only), **intraoperative blood loss.** Lab: **Hb**, **HCT**.		10-fold CV	
Jo *et al*. (2020)[305]	Predict blood transfusion after total knee arthroplasty	1,686 patients<br><br>(108 transfused)	Demographics: **age**, **weight**. Surgical procedure: **surgery type**, **TXA**. Lab: preoperative **Hb**, **PLT**.	GMB	10-fold CV<br><br>EV (n=400)	GBM accurately predicted transfusion (AUC = 0.842 internal, 0.880 external).
Mohammed *et al*. (2022)[306]	Predict surgical outcomes (including blood transfusion) after TKA	636,062 patients<br><br>(73,020 transfused)	Demographics: age, sex, race, insurance type, median household income, **patient location**. **Hospital Data: a**dmission type, **admission month and year**, hospital ownership, hospital bed size. Clinical: **anemia**, coagulopathy, SAH, DM, obesity, HF, pulmonary disease.	LR, GBM, RF, ANN	Hold-out<br>Training: 50%<br>Testing: 30%<br>Validation: 20%	ANN performed best for predicting blood transfusion (AUC 0.812; Brier Score 0.088).
Seong *et al*. (2024)[307]	Predict RBC transfusion in geriatric patients undergoing hip arthroplasty	19,110 patients<br><br>(13,894 transfused)	Demographics: **age**, **sex**, **socioeconomic status**. Clinical: **anemia**, thrombocytopenia, hemiplegia, connective tissue disease, **dementia**, **CHF**, cardiovascular disease, COPD, DM; CKD, PVD, liver disease, solid tumor, leukemia, lymphoma, MI, SAH, peptic ulcer disease, hypothyroidism, thyrotoxicosis, thrombocytopenia, AIDS, medication (**iron**, statins, antithrombotic. Surgical procedure: **anesthesia type**, **TXA**.	RF	Hold-out<br>Training: 80%<br>Testing: 20%<br><br>Out-of-bag	RF achieved a good performance (AUC 0.736).
Zang *et al*. (2024)[308]	Predict perioperative RBC transfusion in hip surgery	2,431 hip surgeries<br><br>(614 transfused)	Demographics: age, sex, BMI. Clinical: **ASA**, SAH, DM, CHD, **anemia**, medication (anticoagulation, antiplatelet use), emergency vs. elective, diagnosis. Surgical procedure: anesthesia type, **operation time**, **surgery type**, **autotransfusion use**, TXA. Lab: **Hb**, PLT, APTT, PT, DD, **FIB**, ALT, Tbil, Dbil, **ALB**, creatinine, BUN.	Ridge, RF, XGB, AdaBoost, ET, CatBosst, NB, LGBM, SVM, DT, KNN, GB, LDA, LR	Hold-out<br>Training: 70%<br>Testing: 30%<br><br>10-fold CV	Ridge Classifier performed best (AUC 0.85, Brier score 0.21, accuracy 79%).

Zhou *et al*. (2024)[309]	Predict intraoperative RBC transfusion in hip fracture surgery	2,785 patients<br><br>(209 transfused)	Demographics: **age**, **gender**. Clinical: **ASA**, **DM**, SAH, cardiac disease, **stroke**, DVT, **hypoproteinemia**, **anemia**. Surgical procedure: **estimated duration of surgery**, **surgery type**. Others: **wait time to surgery**.	RF, LR, SVM, MLP, XGB, GBDT	Hold-out Training: 80% Testing: 20%<br><br>5-fold CV<br><br>EV (n=122)	RF performed best (AUC = 0.887 (internal), 0.834 (external), sensitivity = 90.6%, and specificity = 78.1%).
Zhu *et al*. (2024)[310]	Predict postoperative RBC transfusion in total hip arthroplasty	441 patients<br><br>(94 transfused)	Demographics: age, BMI. Clinical: ASA, medications (**anticoagulation**, **TXA**). Surgical procedure: **intraoperative blood loss**, **estimated surgical duration**. Lab: **Hb**, **ALB**.	LR, SVM, KNN, MLP, NB, DT, RF, GB	Hold-out Training: 90% Testing: 10%<br><br>10-fold CV<br><br>EV (n=388 from 2 independent hospitals)	LR performed best, with AUC 0.98 (internal) and AUC 0.93-0,94 (external).
**Pediatrics**						
Liu *et al*. (2024)[311]	Predict pulmonary hemorrhage in RDS	309 patients<br><br>(48 with pulmonary hemorrhage)	Clinical: gender, **gestational age**, **birth weight**, **Apgar scores**, invasive ventilation, PDA, neonatal asphyxia, intubation in the delivery room, invasive ventilation on the 1st day, surfactant use, septicemia, image classification of RDS. Lab: **PaO_2/FiO_2**, PLT, **MPV**, PT, APTT. Maternal factors: age, cesarean section, multiple pregnancy, amniotic fluid contamination, umbilical cord abnormality, placentae abnormality, GDM, pregnancy-induced SAH, prenatal infection, antenatal steroids.	LR, RF, XGB	4-fold CV	RF model performed best (AUC 0.868, accuracy 88.7%).
**Trauma**						

El-Menyar *et al*. (2024)[312]	Predict activation of the MT protocol	17,390 patients (664 transfused)	Demographics: age. Clinical: BMI, scene and trauma room: **pulse pressure** and rate, **SBP**, **DBP**, **MAP**, **SI**, delta shock index, **diastolic shock index**, **GCS**, revised trauma score, ISS, **triage level**.	KNN, LR, DT, SVM, RF, XGB, ANN	Hold-out Training: 80% Testing: 20% 5-fold CV	RF performed best (AUC 0.95).
Feng *et al*. (2021)[313]	Predict RBC transfusion demand in trauma	1,371 patients (188 transfused)	Demographics: age, gender, height, weight. Clinical: HR, RR, BP, SI, endotracheal intubation, **vasoactive drugs**, ISS, trauma location or type temperature. Lab: K, SpO_2, Hb, **HCT**, **PLT**, **CRP**, IL-6, PT, **PTA**, APTT, INR, **FIB**, **pH**, PaO_2, **$PaCO_2$**, **TCO_2**, lactate, **bicarbonate**.	XGB, CART, LR	Hold-out Training: 80% Testing: 20%	XGB performed best (AUC 0.94, sensitivity 94%, and specificity 82%).
Hodgman *et al*. (2018)[314]	Predict activation of the MT protocol	1,245 patients (297 required MT)	Clinical: HR, SBP, **HR/SBP ratio**, **BD**, **mechanism of injury** (blunt, gunshot wound, stab wound).	LASSO	Not performed EV (n=1,245)	**LASSO-based app model** moderately predicted **MT protocol activation and MT administration** (AUC 0.694–0.711).
Lammers *et al*. (2022)[315]	Predict the need for MT in trauma patients	22,158 patients (1,639 required MT)	Demographics: age, gender. Clinical: mechanism of injury, explosion involvement, HR, RR, SBP, DBP, SpO_2, temperature, GCS, intubation status. Lab: INR, HCT, PLT, pH, $PaCO_2$, PaO_2, BD. Others: prehospital blood products required, procedure emergent required, TXA.	LR, SVM, NN, RF	Hold-out Training: 70% Testing: 30% 10-fold CV	RF performed best (accuracy 95.9%, precision 95.4%, F1 score 96.2%, and AUC 0.984).
Nederpelt *et al*. (2021)[316]	Predict MT, shock, and definitive surgical therapy after truncal gunshot wounds	29,816 patients (1,768 required MT)	Demographics: **age**, sex, BMI, race. Clinical: **HR**, **SBP**, temperature, RR, SaO_2, **GCS**, **gunshot wound anatomical location**, transfer status, blood alcohol, drug screen. Others: time from dispatch to evaluation.	LR, KNN, SVM, RF, DNN, DNN-IAD	Hold-out Training: 80% Testing: 20%	DNN-IAD performed best (AUC 0.86) for predicting MT.

Nikouline *et al*. (2024)[317]	Predict MT in trauma patients	326,758 patients<br><br>(18,871 required MT)	**Demographics: age, sex, race, BMI. Clinical: GCS, ISS, SBP, HR, RR, SaO_2, smoking**, **mechanism of injury**, shock indicators (**pulse oximetry**, **time in ED**). Others: **time at scene**, **use of protective equipment**.	XGB, RF, GB, AdaBoost, MLP, LinearR	Hold-out<br>Training: 80%<br>Testing: 20%	**XGB performed best (AUC 0.83, sensitivity 83%, specificity 84%).**
Shahi *et al*. (2020)[318]	Predict MT, mortality and need for operative management in blunt solid organ injury	477 patients<br><br>(27 patients required MT)	**Demographics: age, gender, weight. Clinical: GCS, HR, BP, SIPA, intubation in ED,** fluid administered pre-hospital and in-hospital, **blood transfusion volumes within 4 hours**. **Imaging:** FAST, CT injury grade. Lab: Hb (serial), **BD**, INR, lactate, **TEG values**.	DNN	Hold-out<br>(MT model)<br>Training: 8%<br>Testing: 92%	**DNN achieved high performance (AUC 0.90) for predicting MT.**
Strickland *et al*. (2023)[319]	Predict MT in trauma patients	2,438 patients<br><br>(121 required MT)	**Demographics: age, sex, BMI. Clinical: SBP, DBP, HR, RR, SpO_2, mechanism of injury (penetrating, blunt), GCS. Bedside imaging: eFAST, pelvic X-ray (pelvic fracture).**	LR, LASSO, Ridge, CART, RF, SVM, NB, XGB, NN, AdaBoost	5-fold CV	LR performed best (AUC 0.83), outperforming existing scores (ABC and RABT).
Sullivan *et al*. (2023)[320]	Predict blood transfusion after pediatric injury	241,729 patients<br><br>(6,871transfused)	Demographics: age. Clinical: **prehospital** and **ED SBP**, HR, RR, SpO_2, **ED GCS**, ED temperature, prehospital CPR, **mechanism of injury**, number of injured body regions, AIS-coded injury to abdomen/pelvis and thorax.	BBN	Hold-out<br>Training: 15%<br>Testing: 43%<br>Recalibration: 42%	BBN achieved excellent performance (AUC 0.92); better predictive performance compared to SIPA and rSIG scores.
Walczak *et al*. (2005)[321]	Predict transfusion needs for trauma patients in the EHRs	1,016 patients<br><br>(508 transfused)	Demographics: age, **sex**, race. Trauma characteristics (**etiology** such as assault, fall, or fire; **type of trauma**: blunt vs. penetrating; use of safety equipment). Clinical: **GCS**, RR, **SBP**.	Multinominal LR, ANN	Hold-out<br>Training: 53%<br>Testing: 47%	ANN achieved a MAE of 7.02 units, outperforming Multinominal LR.
**Urology**						
Engel *et al*. (2021)[322]	Predict blood transfusion in cystectomy	1,168 patients<br><br>(370 transfused)	Demographics: **age**, sex. Clinical: CCI, **anticoagulant use**, **tumor stage**, positive nodal status, neoadjuvant chemotherapy. Interaction terms: S**ex/ blood loss ratio**. Procedure: operation time, **intraoperative**	LR, ENLR, SVM, RF	Hold-out<br>Training: 90%<br>Testing: 10%	LR performed best (AUC 0.87).

			**blood loss**, norepinephrine use, crystalloids use. Lab: **Hb**, PLT.		10-fold CV	
**Vascular**						
Li *et al*. (2024)[323]	Predict bleeding requiring transfusion in lower extremity open revascularization	24,309 patients (4,041 experience bleeding)	**Demographics: a**ge, sex, BMI, race, ethnicity, origin status. Clinical: symptom status (**chronic limb-threatening ischemia**), ASA, SAH, DM, smoking, **CHF**, COPD, **dialysis**, **functional status**, **urgency of surgery**, medications (**antiplatelets**, statins, Beta blockers), **limb hemodynamics (**ABI, toe pressure, palpability of pedal pulses, **prior bypass or endovascular intervention in treated segment**. Lab: Na, BUN**, creatinine**, ALB, WBC, HCT, PLT, INR, PTT.	XGB, RF, NB, SVM, MLP, LR	Hold-out Training: 70% Testing: 30% 10-fold CV	XGB performed best in predicting bleeding events that required transfusion (AUC 0.92, accuracy 84%, sensitivity 82%, specificity 86%).
**Multiple Specialities**						
Hayn *et al*. (2017)[324]	Predict blood transfusion in total hip, total knee replacement and CABG	6,530 elective surgeries (not explicitly stated)	**Demographics: age, gender, weight, height, BMI, BSA. Clinical: t**ype of surgery, surgical technique, ASA. **Surgical procedure:** Blood volume, **estimated RBC volume**, lost RBC volume, **transfused RBC volume**, ordered RBC volume. Lab: **Hb**, **HCT**, PLT aggregation inhibitors, TXA usage.	RF	Leave-10%-Out CV	**RF outperformed traditional RBC ordering methods** in predicting **transfused RBC volume** (Correlation coefficient: **0.61 vs. 0.39 using Mercuriali algorithm**).
Kwon *et al*. (2024)[48]	Predict MT during surgery	18,135 patients (279 received MT)	**Demographics: age, sex. Clinical: SBP, DBP, MBP, pulse transit time, SAH, DM, COPD, tuberculosis, asthma, disease affecting the liver, heart, thyroid, kidney, hematological disease, vascular disease. Intraoperative hemodynamic variables: systolic peak, onset of systolic wave, cycle duration, area under the waveform, velocity and acceleration of PPG. Lab: Hb, HCT, PLT, creatinine, BUN, AST, ALT, ALB, PT, Na, K, glucose.**	LR, RF, XGB, LGBM, RNN, LSTM, GRU	Hold-out Training: 80% Testing: 20% 5-fold CV EV (n=621)	GRU performed best (AUC: 0.962 internal, 0.922 external).
Lee *et al*. (2022)[325]	Predict intraoperative MT across different surgeries	17,986 patients (406 received MT)	**Preoperative variables – Demographics: age, sex, weight, height. Clinical: ASA, SAH, DM, heart disease, liver disease, COPD, asthma, thyroid disease, renal disease, hematologic disease, neurological disease, vascular disease, tuberculosis, pregnancy. Procedure: surgery department, anesthesia type, emergency vs. elective surgery. Lab: Hb, HCT, PLT, ALB, AST, ALT, Na, K, Cl, BUN,**	Preoperative: LR, LASSO, Ridge, RF, GB; Real-time: GRU	Hold-out Training: 70% Testing: 30% 5-fold CV	GRU predicted massive transfusion 10 minutes ahead with AUCs of 0.972 (internal) and 0.943 (external), outperforming preoperative-only models.

			**creatinine, eGFR, glucose, PT, APTT, HDL. Intraoperative variables – SBP, DBP, MBP, HR, SpO_2, ST segment, AUAW, HCT.**		EV (n=494)	
Lou *et al*. (2022)[46]	Improve transfusion risk prediction	4,126,058<br><br>(96,518 transfused)	Demographics: **age**, sex, height, weight. Clinical: SAH, HF, smoking, COPD, dialysis, DM. Lab: **HCT**, **PLT**, **INR**, **APTT**, **creatinine**, Na, ALB, Tbil; Surgical procedure: elective status, **procedure-specific transfusion rate**.	LR, DT, RF, GBM	Training: 80%<br>Testing: 20%<br><br>5-fold CV<br><br>EV (n=16,053)	GBM performed best (AUC 0.939), reducing unnecessary type and screen orders by 33%, saving ~$37,000.
Mitterecker *et al*. (2020)[326]	Predict RBC transfusion, MT, and the number of transfusions in hospitalized patients	131,041 patients<br><br>(25,656 transfused and 968 received MT)	Demographics: **age**, sex. Clinical: **CCI**. Admission data: elective vs. emergency, admission year, diagnosed-related group codes, primary & secondary **ICD-10 diagnosis codes**. Transfusion data: RBC, MT, FFP, PLT, cryoprecipitate units. Lab: admission **Hb**.	LR, RF, GBT, NN	Hold-out<br>Training: 90%<br>Testing: 10%<br><br>5-fold CV	GBT achieved the best performance for predicting RBC transfusion (AUC 0.966; F1-score 0.755), whereas predictions for MT and total RBC units transfused were less reliable.
Park *et al*. (2025)[327]	Predict intraoperative blood transfusion in non-cardiac surgery	6255 patients<br><br>(337 transfused)	Demographics: age, sex, height, weight, BMI. Clinical: ASA, SAH, DM, preoperative EKG findings and pulmonary function test results. Surgery procedure: surgery department and type, **surgical approach**, emergency status, **operation time**, anesthesia typ. Lab: **Hb**, PLT, PT, APTT, Na, K, glucose, ALB, AST, ALT, BUN, creatinine.	LR, RF, GB, AdaBoost, XGB	Hold-out<br>Training: 70%<br>Testing: 30%<br><br>5-fold CV	LR performed best (AUC 0.836).
Walczak *et al*. (2020)[328]	Predict perioperative transfusions across a wide range of surgical procedures	750,937 patients<br><br>(41,254 transfused)	Demographics: age, sex, BMI. Clinical: DM. Lab: HCT, PLT, creatinine, INR.	ANN, LR	EV (n=885,502; 45,621 transfused)	ANN outperformed LR (AUC 0.858, sensitivity 75.4%, specificity 70%).
Yao *et al*. (2019)[329]	Efficiently check the appropriateness of blood transfusion across many surgical cases	4,946 patients<br><br>(3,604 with appropriate transfusion)	**Demographics: age, weight, hospital, type of admission. Clinical: preoperative anemia**, **ASA**, SAH, cardiovascular disease, COPD, DM. Surgical procedure: surgery type, surgery duration, **volume of blood loss**. Lab: **Hb**.	MLPNN, RBM	Not explicitly stated	MLPNN matched physician transfusion decisions with 96.8% accuracy (99% for appropriate, 90.9% for inappropriate cases).

Zapf *et al*. (2023)[47]	Predict intraoperative RBC transfusion across a wide range of surgeries	100,813 surgeries<br><br>(5,448 transfused)	Demographics: age, sex, race/ethnicity, BMI. Surgical procedure: **surgical procedure**, **surgeon**, **anesthesiologist**, urgent status. Lab: **Hb**, PLT, APTT, PT/INR ratio.	LGBM, DT, AdaBoost, RUSBoost, SMOTE-Boosted DT, LR, SMOTE-Boosted LR	Hold-out Training: 60% Testing: 20% Validation: 20%<br><br>EV (n=35,396; 1,776 transfused)	LGBM performed best (AUC 0.93, accuracy 76%, sensitivity 91%).

Table S4. Other Fields of Indication.

Reference (Year)	PBM Application	Sample Size (case group)	Model Variables Top Predictors (bold)	Models	Model Validation	Key Findings
**Support Transfusion decisions**						
Bruun-Rasmussen *et al*. (2022)[52]	Assess the impact of donor sex on RBC transfusion mortality	90,917 patients<br><br>(6,155 male and 5,581 females died within 28 days)	Demographics: age, **sex**. Clinical: CCS angina classification, average **number of RBC transfusions**, **donor sex**, ABO blood group, RhD status. Others: **type of hospital**, medical specialties.	LR, LASSO, MARS, XGB	5-fold-CV	TMLE with Super Learning (XGB & MARS) showed a 2.06% higher survival in male patients receiving RBCs from male vs. female donors, with no significant effect in female patients.
Epah *et al*. (2022)[330]	Predict Hb and iron content in RBC units	8,695 units<br><br>(NA)	**Unit volume**, **donor Hb** (fingertip test), **donor sex**, whole blood donation volume, separated plasma volume, hematocrit, production site, production date, type of blood bag system, production machine, and program.	MLinearR, RANSAC, SVM, LGBMR, KNN, DT, NN	Hold-out Training: 80% Testing: 10% Validation: 10%<br><br>10-fold CV	MLinearR performed best (adjusted R^2=0.91, MAPE=1.43g Hb/4.96mg iron).

Nguyen *et al.* (2020)[53]	Evaluate how transfusion ratios of plasma and PLT to RBC affect hemostasis and mortality in trauma patients	680 patients<br><br>Patients randomized to a 1:1:1 transfusion ratio (cases) versus 1:1:2 (controls)	Demographics: age, gender, ethnicity, BMI. Clinical: SBP, DBP, RR, HR, temperature, FAST, Revised trauma score, injury mechanism, treatment group assign (1.1.1 or 1.1.2 ratio), R-time in TEG, ABS. Lab: INR, PT, APTT, FIB, lactate, pH, BD, Hb, HCT, PLT, WBC.	TMLE	Bootstrap	TMLE showed that high transfusion ratios (plasma and PLTs >0.75 relative to RBCs) significantly improved hemostasis (RR = 2.49, 95% CI: 1.19–5.22), while no significant mortality benefit was found.
Portela *et al.* (2025)[54]	Estimate effects of restrictive vs. liberal transfusion strategies in anemic patients with acute MI	3,447 patients<br><br>(1,729 received the restrictive strategy)	Demographics: age, sex. Clinical: **prior MI**, HF, AF, stroke, **bleeding**, SAH, DM, PAD, MI-related factors (MI type, **LVEF**), RBC transfusion. Lab: **Hb**, **eGFR**.	Super Learner Ensemble (XGB, RF, GBM, LR), LASSOR	10-fold CV	**SL + LASSOR -** best 30-day MACE<br>risk: **15.2% vs. 17.5% (liberal)** and **19.1% (restrictive)**; absolute risk reduction: **−2.3% vs. liberal, −4.0% vs. restrictive.**
**Transfusion safety**						
Borgmann *et al.* (2016)[331]	Accurately classify Rhesus D antigen phenotypes	51 human blood samples (12 D+, 14 weak D, 12 DEL, and 13 D−)<br><br>(NA)	**Number of fluorescence peaks**, **cell intensity** and standard deviation, **peak density** and distribution, average intra-cell and inter-cell peak distances, intensity ratios of different **fluorescence** markers.	RF, SVM, GP, KNN	5-fold CV	**RF was the best-performing model,** achieving **96% overall accuracy.**
Chang *et al.* (2024)[55]	Optimize RH genotyping using WES	6,456 patients<br><br>(NA)	**Genomic sequencing data with features such as: RHD zygosity and hybrid alleles, RHCE*C vs. RHCE*c allele differentiation, RHD c.1136C>T zygosity, RHCE c.48G>C zygosity.**	XGB	Hold-out<br>Testing: 75%<br>Training: 25%<br><br>EV (n=3,030)	XGB improved RH typer's prediction accuracy for RH genotyping, increasing concordance between WES and WGS predictions to 97.2% for RHD (96.2% in EV) and 98.2% for RHCE (96.2% in EV).

Ferraz *et al*. (2017)[332]	Develop and validate a low-cost and reliable system for blood type classification suitable for emergency settings	41 blood tests<br><br>(NA)	Image-derived variables including standard deviation, pixel intensity histogram, histogram of oriented gradients, and fast Fourier transform extracted from regions of interest in the blood test images.	SVM, Bagging, RF, AdaBoost, LPBoost	Hold-out<br>Training: 70%<br>Testing: 30%	SVM with circular ROIs and histogram features achieved perfect classification performance, reaching an F1-score of 1.0.
Fung *et al*. (2024)[333]	Classify posttransfusion adverse events	36 cases<br><br>(NA)	**Clinical Case Data:** Hypothetical transfusion reactions including allergic reactions, febrile non-hemolytic transfusion reactions, AHTR, TACO, TRALI, DSTR, PTP, TAD, TA-GVHD, TTI.	**ChatGPT-3.5 (NLP-based ML model)**	NA	ChatGPT-3.5 correctly classified 48.7% of transfusion reactions (vs. 72.1% by TM specialists) but outperformed them in AHTR/DHTR (100% accuracy) and TRALI.
Hyvärinen *et al*. (2024)[56]	Predict blood group antigens and HPA-1 from genotyping data	112,859 blood donors<br><br>(binary classification: antigen-positive or antigen-negative)	**Genetic Variables:** Genotyping array data for 39 RBC antigens across 14 blood group systems and HPA-1. **Blood Group Antigen Data:** Antigen typing results from Finnish Blood Service Biobank.	**RF, XGB**	Hold-out<br>Training: 50%<br>Testing: 50%<br><br>CV used in XGB (100 iterations with early stopping)<br><br>EV (n=111,667)	**RF achieved an accuracy of 99.9% in the test dataset and 97.1% in the validation dataset.**
Larpant *et al*. (2022)[334]	Enhance the accuracy of Rh antigen phenotyping	4,692 blood samples<br><br>(NA)	Visual features of agglutination patterns in Rh antigen tests (positive, weakly positive, negative).	DarkNet, DarkNet-53, DenseNet-201, YOLO v4	Hold-out<br>Training: 90%<br>Testing: 10%<br><br>EV (n=48)	YOLO v4-tiny with DenseNet-201 achieved top accuracy—97.6% (negative), 98.8% (positive), 96.4% (weakly positive)—and 100% correct interpretation of ambiguous cases in EV.
Lai *et al*. (2014)[335]	Improve the diagnosis of AIHA	588 Coombs test+ patients	Coombs test strength, GCT anti-IgG titer, IgG1 and IgG3 status, their strengths and dilutions, C3d status and strength, IgG	MLP	Hold-out<br>Training: 67.5%<br>Testing: 32.5%	MLP achieved 94.7% sensitivity and 99.4% specificity.

		(52 with AIHA)	overall strength, and the presence of multiple Igs.			
Mahmud *et al*. (2023)[336]	Detecting anemia using non-invasive lip mucosa images	133 individuals<br><br>(29 with anemia)	Image-derived features—**RGB red values** and **HSV saturation. Demographics: age, sex. Lab: Hb.**	NB, DT, KNN, LR, ANN, SVM	Hold-out<br>Training: 60%<br>Testing: 20%<br>Validation: 20%	NB achieved the highest accuracy (96%).
Moslemi *et al*. (2024)[337]	Predict blood group antigens using genomic data	112,168 blood donors<br><br>(case–control counts by antigen)	**600,000+ genetic polymorphisms**, imputed to **millions of variants** across **36 antigens in 15 blood group systems**	MLP, CNN, DAE+CNN	Training: 60%<br>Testing: 10%<br>Validation: 30%	**DAE + CNN** achieved **F1-scores above 99% for most blood group antigens**
Ngufor *et al*. (2016)[338]	Estimate the causal effects of preoperative plasma transfusion on perioperative bleeding	1,234 patients<br><br>(not explicitly stated)	Demographics: age, gender. Clinical: ASA, MI, CHF, CVD, **CKD**, DM, COPD, **peptic ulcer**, connective tissue disease, tumor, liver disease, dementia, preoperative plasma or RBC transfusion, medications (aspirin, **clopidogrel**, heparin). Surgical procedure: emergency vs. elective surgery. Lab: INR, Hb, PLT, **creatinine**, ALB, APTT.	SVM, NN, AdaBoost, RF, rKNN, GBM	5-fold CV	**AdaBoost performed best (AUC 0.868, sensitivity 73.4%, specificity 82.4%).**
Ngufor *et al*. (2018)[339]	Identify subgroups with differential effects of plasma transfusion	3,135 patients<br><br>(NA)	Demographics: age, gender, height, weight. Clinical: preoperative sepsis, ASA, MI, **CHF**, stroke, CKD, DM, COPD, liver disease, dementia, smoking. Surgical procedure: surgical specialty, **emergency** vs. elective surgery. **Treatment Variables:** plasma transfusion status, RBC transfusion, perioperative medication use. Lab: **INR, Hb, PLT**, creatinine, ALB, APTT.	URF + TMLE (SVM, GBM, ELR, LR within TMLE)	5-fold CV	URF + TMLE identified patient subgroups with distinct transfusion responses, showing plasma transfusion increased bleeding (+14% NCS, +12% IR) while mortality effects varied.
Shung *et al*. (2020)[51]	Predict the composite outcome of transfusion,	1,958 patients	Demographics: age, sex. Clinical: ASA, IHD, HF, renal failure, liver disease, malignancy,	LR, LASSO, Ridge, SVM,	Hold-out<br>Training: 80%	XGB performed best (AUC 0.91 in internal validation

	hemostatic intervention, or death in patients with upper GI bleeding	(875 experienced at least one of the composite outcomes)	medications (aspirin, anticoagulation, thienopyridines, NSAIDs), presentation (pulse, SBP, syncope, mental status, hematemesis, melena. Lab: Hb, urea, creatinine, ALB, INR.	DT, RF, XGB, EN, MLPNN	Testing: 20%<br><br>10-fold CV<br><br>EV (n=399)	and 0.90 in EV), outperforming clinical risk scores: GBS (0.87), Rockall (0.65), and AIMS65 (0.64).
Whitaker *et al*. (2022)[340]	Identify allergic transfusion reactions from EHRs	751 transfusion events<br><br>(146 with reported allergic reactions)	Demographics: age, gender. Clinical: conjunctival edema, periorbital edema, mouth edema, angioedema, rash, itching, hives, flushing, hypotension, bronchospasm, anaphylaxis, airway symptoms, dysphagia, dysphonia, hoarseness, stridor, dyspnea, cough, wheezing, hypoxemia, syncope, hypotonia, **H1 antihistamines** and **glucocorticoids** administration. Others: **NLP-extracted mentions of hives, itching, and transfusion reaction from clinical notes**.	LR, DT, RF, KNN, NB	Hold-out<br>Training: 80%<br>Testing: 20%<br><br>5-fold CV	LR combined with NLP-extracted features was the best-performing model, achieving an AUC of 0.92, 67.9% sensitivity, and 97.5% specificity at a decision threshold of 0.9.
Wu *et al*. (2022)[57]	Automatic classification of IARI in Coombs test images	1,628 IARI - labeled samples<br><br>(NA)	Raw IARI images from U-bottom microplates; each labeled as (-), (1+), (2+), (3+), or (4+) by expert immunologists.	Ensemble of 5 CBAM-enhanced CNNs: AlexNet, VGG, ResNet, Inception, DenseNet	Hold-out<br>Training: 80%<br>Testing: 20%	CBAM-CNN achieved 99.8% accuracy, outperforming all individual CNNs and manual classification by immunologists (average 88.3%).
Zhu *et al*. (2022)[341]	Identify risk factors and predict adverse events during neonatal ET	188 exchange transfusions (185 patients)<br><br>(185 ≥1 adverse event)	Child-related: sex, gestational age, birth weight, weight at admission, **age at admission**, Apgar score, feeding mode, diagnosis and **etiology**, ET parameters, Lab (**Tbil**, **Dbil**, Ibil, **Hb**, WBC, PLT, Ca, K, Na, glucose, bicarbonate, pH. Maternal-related: gravidity, G6PD deficiency, blood group.<br>(top predictors for transfusion are in bold)	XGB, RF, LR, NB, KNN	Hold-out<br>Training: 70%<br>Testing: 30%	XGB showed moderate discriminative ability (AUC 0.71) in predicting adverse events, and when combined with SHAP (explainable AI), it identified both known and

						novel nonlinear risk factors with clinical relevance.
**Hospital Blood Bank Inventory Management**						
Ahmadimanesh *et al*. (2020)[61]	Optimize regional blood inventory management	731 days (NA)	**Daily hospital blood demand**, **return rates**, **hospital order frequency**, **base inventory levels**, **blood donation frequency**, inter-base transfers, **safety reserve thresholds**.	DNN	Hold-out Training: 68% Testing: 32%	DNN accurately predicted demand and optimized inventory, reducing blood returns to zero and maintaining safety stock, with simulation results matching real data ($p > 0.95$).
Cifuentes *et al*. (2020)[342]	Predict blood transfusion needs	4,831patients (not explicitly stated)	Demographics: age. Clinical: ASA, **emergency status**, **surgery subspecialty**, **anemia**, SAH, DM, cardiovascular disease, COPD. Lab: **Hb**. Surgical procedure: surgery duration, **blood loss volume**.	RBM +/-: RF, SVM, DTC, MLPNN	Hold-out Training: 70% Testing: 30% CV performed (folds' number not specified)	RBM-based RF achieved the highest accuracy (96.85%) and F1 score (94.26%).
Engelke *et al*. (2023)[59]	Predict PLT transfusion needs	34,809 patients (~17,500 samples)	Demographics: **age**, gender. Clinical conditions (**leukemia**, **lymphoma**, anemia, **coagulopathies**, **infections**, malignancies, rheumatic diseases), procedures (surgical interventions, **chemotherapy,** palliative care), medications (anti-infective, other drugs). Temporal features (**weekday** vs. weekend schedules), and categorical features (ward and **department location**).	LSTM, RF, XGB	Hold-out Training: 80% Testing: 20% 5-fold CV	LSTM performed best, with moderate performance overall (AUC-PR 0.73) and highest for hematology-oncology patients (AUC-PR 0.84).
Etchels *et al*. (2006)[343]	Identify key factors influencing transfusion decisions in surgeries	209 transfusion decision cases	Demographics: age. Clinical: **risk of tissue hypoxia**, presence of **ongoing hemorrhage**, clinician's perception of cardiac output capacity. Lab: **Hb**.	NN-OSRE	Not explicitly stated)	NN-OSRE achieved **high predictive performance**, with a 96% **specificity, 93% sensitivity of 93%**, and a **PPV of 90%.**

		(not explicitly stated				
Fanoodi *et al*. (2019)[344]	Predict daily PLT demand	1,826 daily PLT demand records over 5 years<br><br>(NA)	Daily demand for each blood type platelet, historical demand trends (delays of 1 to 365 days).	ANN	Hold-out<br>Training: 70%<br>Testing: 15%<br>Validation: 15%	**ANN outperformed ARIMA**, improving **O+ platelet forecasts by 66%** over the baseline and showing **higher accuracy across all blood types.**
Feng *et al*. (2021)[345]	Predict preoperative RBC demand	130,966 surgical episodes<br><br>(40,609 transfused)	Demographics: age, sex. Clinical: weight, height. Procedure: surgical grade, **surgeon name**, **surgical type**, **surgical site**, **surgeon's RBC transfusion history**. Lab: **Hb**, PLT, INR, PT, APTT, FIB, pH, $PaCO_2$ pO_2, HCO_3^-, lactate; others: autologous blood donation, blood product usage trends.	LGBM, XGB, RF, NN, LinearR, CatBoost, Stacking	Hold-out<br>Training: 80%<br>Testing: 20%	LGBM achieved an AUC of 0.908, outperforming clinician predictions in 85% of cases.
Li *et al*. (2022)[58]	Predict RBC demand and optimize inventory management	227,944 RBC transfusions<br><br>(NA)	**Demographics:** age, sex. **Clinical: d**iagnosis, ICU admission, surgery type, outpatient or inpatient Status. **Operational:** number of daily transfusions, number of ABO non-identical transfusions, number of Rh^- units to Rh^+ recipients daily, number of units transfused daily to different patient types, number of daily transfusions per patient, **weekday of transfusion**. **Lab:** Blood type, Hb, PLT, **MPV**, **RDW**, **IgG**, **INR**, creatinine.	LSTM, STL, STL+XGB, STL+LinearR	Hold-out<br>Training: ~85%<br>Testing: ~15%<br><br>5-fold CV	**STL+XGB performed best (RSME=18.32, MAPE=15.9%),** reducing inventory by 38.4%, eliminating urgent orders, and cutting costs by 43%.
Miri-Moghaddam *et al*. (2024)[346]	Forecast blood product trends in Iran to support PBM	Monthly blood product data from 38 blood centers over 10 years<br><br>(NA)	Aggregated monthly supply and distribution data for five blood products: RBC, LRRBC, FFP, PLT, PLT-Apheresis.	LSTM	Hold-out<br>Training: 70%<br>Testing: 30%	LSTM performed better than classical time series models (lower RMSE/MAPE) for RBC, LRRBC, and FFP forecasts; classical models performed better for PLT-Apheresis.

Motamedi *et al*. (2024)[347]	Predict daily PLT demand	61,377 PLT transfusions (from 47,496 patients)<br><br>(NA)	Lab: PLT, Hb, creatinine, INR, MCH, MCV, RDW, WBC, LYMP, HCT, ALP, MPV, PaO_2. Patient location (General Medicine, Hematology, ICU, Cardiovascular surgery, Pediatrics). Others: weekday, operational data (previous day and week transfusions, received units).	LASSO, RF, LSTM	Hold-out with rolling-origin evaluation, using historical training data and unseen 2018 test data<br><br>5-fold CV (LASSO, RF)<br>10-fold CV (LSTM)	LASSO was the best-performing method overall, offering high accuracy and interpretability, with RMSE around **4.9** and MAPE near **19.7%**, outperforming univariate models like ARIMA and Prophet.
Sarvestani *et al*. (2022)[348]	Predicting monthly blood demand by blood group	96 monthly observations per blood group (2012-19)<br><br>(NA)	Monthly demand for blood groups (A+, A−, B+, B−, AB+, AB−, O+, O−).	ANN, ARIMA, ARIMA+ANN	Hold-out<br>Training: 84 months<br>Testing: 12 months	ARIMA outperformed ANN and hybrid model for most blood groups, except O+ and O-.
Shih *et al*. (2019)[349]	Forecast blood supply and improve inventory decisions	5 years of weekly supply data (2013-17)<br><br>(NA)	Time-based: date, weekday, seasonality. Past supply volumes.	ANN, MR	Hold-out<br>Training: 2013-17<br>Testing: 2018	**Time series models outperformed ML models.**
Sun *et al*. (2021)[60]	Predict daily RBC demand and improve inventory management	1,243 days of RBC usage and inventory data<br><br>(NA)	Time-based: day of the week, month, and year. Holiday-related: days before and after holidays. Hospital activity indicators: historical averages and standard deviations of RBC use per department, application dates, requested quantities. Purpose of transfusion (surgical or therapeutic). Historical consumption trends.	XGB	Hold-out (ratio training/testing not stated)	XGB accurately predicted daily RBC demand with low MAE across all blood types (A: 10.69, B: 11.19, O: 10.93, AB: 5.91), enabling improved inventory planning and reducing transfusion-related supply risk.

Xiang *et al*. (2021)[62]	Identify areas of blood product wastage	879,532 transfusion transactions<br><br>(NA)	Product description, ABO and Rh type, special treatments, status in Blood Bank, **ward location**, **hospital site**, transaction location, **disposal and return reasons**, **time of transaction** entry, time until expiration, season, inventory size, **handling time**, supplier, **technologist shift/time of the day**, transfusion frequency.	Association Rule Mining	NA	The algorithm identified clinically significant, irrelevant, and unexplained associations, revealing both expected and hidden causes of blood product wastage.

Table S5.

Reference (Year)	Title	Reason for Exclusion
Afonso et al. (2021)[350]	Performance of a convolutional neural network for automatic detection of blood and hematic residues in small bowel lumen	Out of PBM scope - focuses on detecting bleeding on endoscopic images
Aiwale et al. (2024)[351]	Non-invasive anemia detection and prediagnosis	Methodological/reporting limits - ML used for nail image-based anemia detection but lacks clear hold-out or control group structure, dataset description, and validation design.
Akazawa et al. (2024)[352]	Prediction of hemorrhage in placenta previa: Radiomics analysis of pelvic MRI images	No ML/DL models used (LR only).
Alnor et al. (2025)[353]	Natural language processing for identifying major bleeding risk in hospitalized medical patients	No ML/DL model used - NLP applied for text-based event identification, but predictive models developed using traditional Cox regression.
Aminsharifi et al. (2017)[354]	Artificial neural network system to predict the postoperative outcome of percutaneous nephrolithotomy	Outcomes presented as a composite (ANN predicts multiple postoperative variables together).
Aoki et al. (2020)[355]	Automatic detection of blood content in capsule endoscopy images based on a deep convolutional neural network	Out of PBM scope - DL model detects blood in capsule endoscopy images (diagnostic image analysis).
Asadi et al. (2014)[356]	Machine learning for outcome prediction of acute ischemic stroke post intra-arterial therapy	Out of PBM scope - ML model predicts neurological and functional outcomes after endovascular intervention for acute ischemic stroke.
Asadi et al. (2016)[357]	Outcomes and complications after endovascular treatment of brain arteriovenous malformations: a prognostication attempt using artificial intelligence	Out of PBM scope - ML model predicts overall outcomes and complications after endovascular AVM treatment.
Ayling et al. (2019)[358]	Potential roles of artificial intelligence learning and faecal immunochemical testing for prioritization of colonoscopy in anaemia	Out of PBM scope - AI algorithm used for diagnostic prioritization of colonoscopy in IDA; focuses on cancer risk stratification.
Babazadeh Khameneh et al. (2012)[359]	Abnormal red blood cells detection using adaptive neuro-fuzzy system	Out of PBM scope - ML-based image analysis model for detecting abnormal RBCs in laboratory microscopy.
Bahar et al. (2023)[360]	Using trends and outliers in managing delayed transfusions	Out of PBM scope - transfusion medicine quality improvement study using data analytics to monitor transfusion delays.
Baiardi et al. (1997)[361]	Use of statistical classifiers as support tools for the diagnosis of iron-deficiency anemia in patients on chronic hemodialysis	Out of PBM scope - diagnostic modeling study in nephrology.
Barbieri et al. (2021)[362]	How to assess the risks associated with the usage of a medical device based on predictive modeling: the case of an anemia control model certified as medical device	Out of PBM scope; no new ML/DL model developed - evaluation of an existing certified ML model for postmarketing risk assessment.

Battles et al. (1998)[363]	The attributes of medical event-reporting systems: experience with a prototype medical event-reporting system for transfusion medicine	No ML/DL model used; out of PBM scope - transfusion medicine quality/safety study describing a prototype event-reporting system.
Beck et al. (1989)[364]	Computer-based exercises in anemia diagnosis (PlanAlyzer)	Out of PBM scope, not in real patients, and no ML/DL model used - educational simulation for teaching anemia diagnostic reasoning in medical students.
Bensalah et al. (2013)[365]	Perioperative outcomes of off-clamp vs complete hilar control laparoscopic partial nephrectomy	No ML/DL model used (multivariable regression analysis)
Birndorf et al. (1996)[366]	An expert system to diagnose anemia and report results directly on hematology forms	Methodological/reporting limits - early hybrid AI model with incomplete reporting of model construction and evaluation; lacks modern transparency and regulatory standards.
Bonatti et al. (2011)[367]	Factors influencing blood transfusion requirements in robotic totally endoscopic coronary artery bypass grafting on the arrested heart.	No ML/DL model used (classical statistical methods only)
Causer et al. (1994)[368]	Assessment of a computerized system for the diagnosis of iron deficiency	No ML/DL model used - fuzzy logic-based expert system for diagnosing iron deficiency.
Chen et al. (2024)[369]	The role of coagulopathy and subdural hematoma thickness at admission in predicting the prognoses of patients with severe traumatic brain injury: a multicenter retrospective cohort study from China	Out of PBM scope - predicts neurological outcome after traumatic brain injury; no new ML/DL model developed - ML used only for variable importance ranking.
Christie et al. (2019)[370]	Dynamic multi-outcome prediction after injury: Applying adaptive machine learning for precision medicine in trauma	Out of PBM scope - transfusion prediction limited to acute trauma resuscitation.
Chu et al. (2018)[371]	Using neural attention networks to detect adverse medical events from electronic health records	Out of PBM scope - DL model detects adverse events (including bleeding) in EHR text.
Chu et al. (2023)[372]	Convolutional neural network-based segmentation network applied to image recognition of angiodysplasias lesion under capsule endoscopy	Out of PBM scope - AI applied to diagnostic image recognition of bleeding lesions.
Convertino et al. (2015)[373]	Individual-Specific, beat-to-beat trending of significant human blood loss: the compensatory reserve	Out of PBM scope - controlled blood loss experiment in volunteers; no ML/DL model.
Convertino & Koons (2020)[374]	The compensatory reserve: potential for accurate individualized goal-directed whole blood resuscitation	Out of PBM scope - animal study on hemorrhage physiology and waveform-based algorithm; no ML/DL model developed.
Convertino et al. (2022)[375]	AI-enabled advanced development for assessing low circulating blood volume for emergency medical care: comparison of compensatory reserve machine-learning algorithms	Out of PBM scope - focuses on experimental shock detection and waveform analysis; uses healthy volunteers.
Darrin et al. (2023)[376]	Classification of red cell dynamics with convolutional and recurrent neural networks: a sickle cell disease case study	Out of PBM scope - AI applied to laboratory RBC motion analysis.
Das et al. (2013)[377]	Quantitative microscopy approach for shape-based erythrocytes characterization in anaemia	Out of PBM scope - ML model applied to microscopic image analysis of erythrocyte morphology for laboratory classification of anemia subtypes.

Dengler et al. (2021)[378]	Outcome prediction in aneurysmal subarachnoid hemorrhage: a comparison of machine learning methods and established clinic-radiological scores	Out of PBM scope - ML models predict neurological and functional outcomes after aneurysmal subarachnoid hemorrhage.
Deshmukh et al. (2020)[379]	Explainable machine learning model for predicting GI bleed mortality in the intensive care unit	Out of PBM scope - ML model predicts ICU mortality in gastrointestinal bleed patients.
Dong et al. (2019)[380]	Machine learning-based development and validation of a scoring system for screening high-risk esophageal varices	Out of PBM scope - ML model predicts presence of esophageal varices and varices needing treatment (diagnostic screening in cirrhosis).
Dumont et al. (2011)[381]	Prediction of symptomatic cerebral vasospasm after aneurysmal subarachnoid hemorrhage with an artificial neural network: feasibility and comparison with logistic regression models	Out of PBM scope - predicts cerebral vasospasm after subarachnoid hemorrhage.
Erler et al. (1995)[382]	Superiority of neural networks over discriminant functions for thalassemia minor screening of red blood cell microcytosis	Out of PBM scope - diagnostic classification of thalassemia minor using ANN.
Escandell-Montero et al. (2014)[383]	Optimization of anemia treatment in hemodialysis patients via reinforcement learning	Not in real patients - reinforcement learning model for ESA dose optimization tested only in computational simulation.
Farooq et al. (2025)[384]	Developing a transparent anaemia prediction model empowered with explainable artificial intelligence	Methodological/reporting limits - ML model for anemia prediction lacks dataset description, sample size, and validation details.
Ferret et al. (2013)[385]	Evaluation of a computerized tool allowing retrospective detection of potential vitamin K antagonist overdoses in complex contexts	Out of PBM scope - focuses on anticoagulant overdose identification; no ML/DL model used - rule-based adverse drug event detection tool without ML modeling.
Formeister et al. (2020)[386]	Machine learning for predicting complications in head and neck microvascular free tissue transfer	Outcomes presented as a composite - ML model predicts combined surgical complications including bleeding.
Ge et al. (2021)[387]	Cell mechanics based computational classification of red blood cells via machine intelligence applied to morpho-rheological markers	Out of PBM scope - unsupervised ML applied to label-free classification of RBC subtypes (reticulocytes vs. mature RBCs) using morpho-rheological data.
Ghobadi & Saraf Esmaili. (2024)[388]	Use of a neuro-fuzzy technique to predict complete Rockall score in patients with upper gastrointestinal bleeding	Out of PBM scope - neuro-fuzzy predicts composite Rockall score for upper GI bleeding.
Gómez et al. (2025)[389]	Anemia classification system using machine learning	Out of PBM scope - ML model classifies anemia into morphological types (microcytic, normocytic, macrocytic) but does not differentiate etiologies.
Graham (2015)[390]	Transfusion e-learning for junior doctors: the educational role of 'LearnBloodTransfusion'	Out of PBM scope; no ML/DL model used - educational e-learning program for transfusion training and competency development in junior doctors.
Guo et al. (2024)[391]	Evaluation of artificial intelligence-assisted morphological analysis for platelet count estimation	Out of PBM scope - AI applied to laboratory PLT count estimation (analytical validation).

Haghani et al. (2017)[392]	Artificial neural network to modeling zero-inflated count data: application to predicting number of return to blood donation	Out of PBM scope (blood donation/return prediction).
Hahn-Klimroth et al. (2021)[393]	Generation and validation of a formula to calculate hemoglobin loss on a cohort of healthy adults subjected to controlled blood loss	Not in real patients (limited to healthy adults).
Hurley et al. (2023)[394]	Would doctors dream of electric blood bankers? Large language model-based artificial intelligence performs well in many aspects of transfusion medicine	Not in real patients - study evaluated used simulated transfusion scenarios and knowledge-based tasks.
Imbert et al. (1989)[395]	An expert system applied to the diagnosis of anemia with special reference to myelodysplastic syndromes	No ML/DL model used - rule-based Bayesian expert system.
Isiksacan et al. (2023)[396]	Assessment of stored red blood cells through lab-on-a-chip technologies for precision transfusion medicine	Out of PBM scope - conceptual perspective on RBC storage quality assessment; no ML/DL model developed.
Jiang et al. (2021)[397]	Machine learning for the prediction of complications in patients after mitral valve surgery	Out of PBM scope – complications analyzed not relevant in the PBM context.
Jørgensen et al. (2024)[398]	Preoperative hemoglobin thresholds for increased risk of 'medical' complications in fast-track total hip and knee arthroplasty, a secondary analysis of a machine-learning algorithm	No ML/DL models used - secondary analysis of existing ML model.
Kang et al. (2023)[399]	Observation-informed modeling of artificial neural networks to predict flow and bleeding of cement-based materials	Out of PBM scope - ANN predicts flow and material bleeding rate in cement mortars.
Karkouti et al. (2001)[400]	A multivariable model for predicting the need for blood transfusion in patients undergoing first-time elective coronary bypass graft surgery	No ML/DL model used - traditional multivariable LR model for transfusion prediction in CABG patients.
Kassaw et al. (2023)[401]	The application of machine learning approaches to determine the predictors of anemia among under five children in Ethiopia	No ML/DL predictive model developed - Boruta algorithm used only for feature selection without building or testing a predictive model.
Kimura et al. (2019)[402]	A novel automated image analysis system using deep convolutional neural networks can assist to differentiate MDS and AA	Out of PBM scope - DL model differentiates hematologic diseases (MDS vs. aplastic anemia) from peripheral blood smear images.
Kneifati-Hayek et al. (2007)[403]	A model for automated screening of thalassemia in hematology (math study)	No ML/DL model used - rule-based diagnostic index.
Knowlton et al. (2015)[404]	Sickle cell detection using a smartphone	Out of PBM scope - diagnostic device study for SCD detection using smartphone-based magnetic levitation.
Kolin et al. (2023)[405]	Predicting postoperative anemia and blood transfusion following total knee arthroplasty	No ML/DL models used - traditional multivariable LR models for predicting postoperative anemia and transfusion.
Koons et al. (2020)[406]	Combat medic testing of a novel monitoring capability for early detection of hemorrhage	Out of PBM scope - simulation study of combat medics using existing ML algorithm for hemorrhage detection; not in real patients.

Kordzadeh et al. (2021)[407]	Prediction, pattern recognition and modelling of complications post-endovascular infra renal aneurysm repair by artificial intelligence	Out of PBM scope - AI/ML models predict complications not relevant to PBM.
Kunze et al. (2022)[408]	Development and internal validation of machine learning algorithms for predicting complications after primary total hip arthroplasty	Out of PBM scope - ML models predict general postoperative complications after THA not relevant to PBM.
Kurmanaliyev et al. (2025)[409]	An integrative machine learning model for predicting early safety outcomes in patients undergoing transcatheter aortic valve implantation	Outcomes not separated - ML model predicts a composite endpoint without separate performance metrics.
Lee et al. (2021)[410]	Predicting outcomes after trauma: prognostic model development based on admission features through machine learning	Out of PBM scope - ML model predicts mortality in trauma patients based on admission features.
Li et al. (2017)[411]	New dandelion algorithm optimizes extreme learning machine for biomedical classification problems	Out of PBM scope - computational optimization of an ML algorithm for general biomedical classification.
Liu et al. (2013)[412]	Prediction of perioperative outcomes following minimally invasive partial nephrectomy: role of the R.E.N.A.L. nephrometry score	No ML/DL model used - multivariable regression analysis.
Lo et al. (2013)[413]	Clinical outcome prediction in aneurysmal subarachnoid hemorrhage using Bayesian neural networks with fuzzy logic inferences	Out of PBM scope - ML model predicts neurological outcomes in aneurysmal subarachnoid hemorrhage.
Luo et al. (2024)[414]	Impact of fresh frozen plasma transfusion on mortality in extracorporeal membrane oxygenation	No ML/DL predictive model developed; Boruta algorithm used only for feature selection prior to LR.
Mahapatra et al. (2024)[415]	Non-invasive hemoglobin screening device: a promising digital method for reducing anemia prevalence through routine screening and timely intervention	Methodological/reporting limits - study used a commercial device with unspecified internal algorithm; no explicit ML model development, training, or validation reported.
Mascarella et al. (2022)[416]	Above and beyond age: prediction of major postoperative adverse events in head and neck surgery	Outcomes analyzed only as part of a composite postoperative adverse event endpoint, not separately.
Merath et al. (2020)[417]	Use of machine learning for prediction of patient risk of postoperative complications after liver, pancreatic, and colorectal surgery	Outcomes not separated (model predicts composite "bleeding requiring transfusion" event, not transfusion or bleeding individually).
Merrill et al. (2019)[418]	Machine learning accurately predicts short-term outcomes following open reduction and internal fixation of ankle fractures	Out of PBM scope - ML model predicts general postoperative outcomes (morbidity, mortality, readmission, and LOS).
Meyer et al. (2024)[419]	TiME OUT: time-specific machine-learning evaluation to optimize ultramassive transfusion	Out of PBM scope - predicts mortality in patients already transfused.
Mikić et al. (2024)[420]	The advantages of using tranexamic acid in anterior cruciate ligament reconstruction: a randomized controlled trial	Out of PBM scope - clinical trial evaluating TXA to reduce postoperative bleeding after ACL reconstruction; no ML/DL model used.
Miyagi et al. (2020)[421]	New method for determining fibrinogen and FDP threshold criteria by artificial intelligence in cases of massive hemorrhage during delivery	Out of PBM scope - AI used to determine coagulation thresholds during active hemorrhage; no ML/DL model used - employs AI-based mathematical modeling and curve fitting.

Miyagi et al. (2024)[422]	A novel method for determining fibrin/fibrinogen degradation products and fibrinogen threshold criteria via artificial intelligence in massive hemorrhage during delivery with hematuria	Out of PBM scope - ML used to determine coagulation thresholds for hematuria during massive obstetric hemorrhage.
Moreillon et al. (2023)[423]	Prediction of plasma volume and total hemoglobin mass with machine learning	Out of PBM scope and not in real patients - ML model developed for physiological and sports research to estimate Hb mass and plasma volume in healthy/athlete populations.
Moura et al. (2020)[424]	Rapid diagnosis of hereditary haemolytic anaemias using automated rheoscopy and supervised machine learning	Out of PBM scope – supervised ML applied for laboratory diagnosis of hereditary hemolytic anemias (analytical validation).
Narula & Diaper. (1991)[425]	Third world continuing medical education with hypertext: the Liverpool anaemia guide system	Out of PBM scope; no ML/DL model used – hypertext-based educational tool for continuing medical education on anemia.
Nazarian et al. (2024)[426]	Development and validation of machine learning models to predict the need for haemostatic therapy in acute upper gastrointestinal bleeding	Out of PBM scope - ML model predicts the need for hemostatic intervention in acute upper GI bleeding.
Nemeth et al. (2023)[427]	TCCC decision support with machine learning prediction of hemorrhage risk, shock probability	Not in real patients – ML model predicts hemorrhage and transfusion needs using simulated or external datasets.
Nguyen et al. (1996)[428]	A rule-based expert system for laboratory diagnosis of hemoglobin disorders	Out of PBM scope - focus on rule-based diagnosis of hemoglobinopathies; no ML/DL model used – expert system based on fixed rules.
O'Connor & McKinney (1989)[429]	The diagnosis of microcytic anemia by a rule-based expert system using VP-Expert	Out of PBM scope - focus on diagnostic rule-based interpretation of microcytic anemia; no ML/DL model used - fixed rule-based expert system.
Ohara et al. (2021)[430]	Artificial intelligence supported anemia control system (AISACS) to prevent anemia in maintenance hemodialysis patients	Methodological/reporting limits – within PBM scope but the model mimics physician dosing decisions rather than predicting anemia.
Orlando et al. (2018)[431]	An ensemble deep learning based approach for red lesion detection in fundus images	Out of PBM scope – DL model detects retinal hemorrhages and microaneurysms in diabetic retinopathy fundus images.
Padrão et al. (2022)[432]	Phenotypes of sickle cell intensive care admissions: an unsupervised machine learning approach in a single-center retrospective cohort	Out of PBM scope – unsupervised ML used for ICU phenotyping of SCD patients.
Paterakis et al. (1989)[433]	The performance characteristics of an expert system for the 'on-line' assessment of thalassemia trait and iron deficiency—Micro Hema Screen	No ML/DL model used – rule-based expert system for automated discrimination of thalassemia trait and iron deficiency.
Pollei et al. (2013)[434]	Analysis of postoperative bleeding and risk factors in transoral surgery of the oropharynx	No ML/DL models used.
Qasrawi et al. (2024)[435]	Identification and prediction of association patterns between nutrient intake and anemia using machine learning techniques: results from a cross-sectional study with university female students from Palestine.	Not in real patients.

Ramzan et al. (2024)[436]	Revolutionizing anemia detection: integrative machine learning models and advanced attention mechanisms	Not in real patients - ML/DL model for anemia detection primarily trained and validated on retrospective open-source datasets.
Rangaraj et al. (2022)[437]	Identifying risk factors of intracerebral hemorrhage stability using explainable attention model	Out of PBM scope – DL model predicts intracerebral hemorrhage stability on CT images (diagnostic imaging).
Rao et al. (2022)[438]	Deep transfer learning for automatic prediction of hemorrhagic stroke on CT Images	Out of PBM scope – DL model predicts intracerebral hemorrhage on CT images (diagnostic imaging).
Rashiq et al. (2004)[439]	Predicting allogeneic blood transfusion use in total joint arthroplasty	No ML/DL model used – traditional multivariable LR to predict transfusion risk in total joint arthroplasty.
Rashedi et al. (2024)[440]	Prediction of occult hemorrhage in the lower body negative pressure model: initial validation of machine learning approaches	Not in real patients – ML model predicts hemorrhage onset in simulated lower body negative pressure experiments.
Roubinian et al. (2020)[441]	NT-proBNP levels in the identification and classification of pulmonary transfusion reactions	Out of PBM scope (transfusion complication identification/classification)
Rubbert et al. (2018)[442]	Prediction of outcome after aneurysmal subarachnoid haemorrhage using data from patient admission	Out of PBM scope - ML model predicts neurological functional outcomes after subarachnoid hemorrhage using CT perfusion and clinical data.
Santipas et al. (2025)[443]	Development and validation of machine learning model for prediction of complication after cervical spine metastases surgery	Out of PBM scope - ML model predicts general postoperative complications after cervical spine metastases surgery.
Shah et al. (2023)[444]	Development and external validation of a risk calculator for prediction of major complications and readmission after anterior cervical discectomy and fusion	Outcomes not separated - ML model predicts composite perioperative complications including bleeding.
Sharma et al. (2023)[445]	Development and validation of a nomogram predicting intraoperative adverse events during robot-assisted partial nephrectomy	Transfusion analyzed only as part of a composite outcome; no ML/DL models used (LR).
Shi et al. (2023)[446]	Prognostic value of machine-learning-based PRAISE score for ischemic and bleeding events in patients with acute coronary syndrome undergoing percutaneous coronary intervention	No new ML/DL model developed.
Shrestha et al. (2025)[447]	Evaluation of low-cost techniques to detect sickle cell disease and β-thalassemia: an open-label, international, multicenter study	Out of PBM scope - evaluates low-cost diagnostic for SCD and β-thalassemia detection (laboratory diagnostic application).
Shung et al. (2021)[448]	Early identification of patients with acute gastrointestinal bleeding using natural language processing and decision rules	Out of PBM scope – NLP and decision-rule models identify patients presenting with acute GIB in real time.
Shung et al. (2024)[449]	Validation of an electronic health record-based machine learning model compared with clinical risk scores for gastrointestinal bleeding	Outcomes analyzed only as part of a composite endpoint, not separately.
Shweta & Pande. (2023)[450]	Prediction of anemia using various ensemble learning and boosting techniques	Methodological/reporting limits and not in real patients - ML/ensemble models developed using combined open-source Kaggle datasets for anemia prediction; no clinical or hospital-based validation.

Sielaff et al. (1989)[451]	ESPRE: a knowledge-based system to support platelet transfusion decisions	No ML/DL model used - rule-based expert system for PLT transfusion decision support.
Sielaff et al. (1991)[452]	Design and preliminary evaluation of an expert system for platelet request evaluation	No ML/DL model used - rule-based expert system for triage and review of platelet transfusion requests.
Sofo et al. (2020)[453]	New perspectives in the prediction of postoperative complications for high-risk ulcerative colitis patients: machine learning preliminary approach	Out of PBM scope - ML model predicts overall postoperative complications (infectious and non-infectious) after colectomy.
Sorace et al. (1992)[454]	Automated review of blood donor screening test patterns at a regional blood center	Out of PBM scope - expert system for donor screening and deferral management; no ML/DL model used (rule-based system).
Stewart et al. (2016)[455]	Compensatory reserve for early and accurate prediction of hemodynamic compromise: case studies for clinical utility in acute care and physical performance	Out of PBM scope – focused on hemodynamic monitoring.
Sultan et al. (1988)[456]	Decision-making system (DMS) applied to hematology. Diagnosis of 180 cases of anemia secondary to a variety of hematologic disorders	Out of PBM scope – diagnostic classification of benign and malignant hematologic anemias (hematology-specific).
Susič et al. (2023)[457]	Wellbeing forecasting in postpartum anemia patients	Out of PBM scope - ML models predict wellbeing parameters (depression and fatigue) in postpartum anemia patients.
Tonetti et al. (2020)[458]	Self-reported bleeding on brushing as a predictor of bleeding on probing: early observations from the deployment of an internet of things network of intelligent power-driven toothbrushes in a supportive periodontal care population	Out of PBM scope; no ML/DL models - focused on periodontal care (gingival inflammation).
Tsai et al. (2024)[459]	Machine learning models for predicting mortality in patients with cirrhosis and acute upper gastrointestinal bleeding at an emergency department: a retrospective cohort study	Out of PBM scope - ML model predicts mortality in cirrhotic patients with upper GI bleeding.
Turgut et al. (2021)[460]	Applications of deep learning to the assessment of red blood cell deformability	Out of PBM scope – DL model estimates RBC deformability and rigid-cell fraction in laboratory settings (analytical validation).
Turna et al. (2005)[461]	Prediction of morbidity after lung resection in patients with lung cancer using fuzzy logic	Outcomes, including bleeding, presented as a composite.
Valafar et al. (2000)[462]	Predicting the effectiveness of hydroxyurea in individual sickle cell anemia patients	Out of PBM scope - ANN model predicts pharmacologic response to hydroxyurea therapy in sickle cell anemia (hematology-specific context).
Vicent et al. (2022)[463]	An algorithm to detect overlapping red blood cells for sickle cell disease diagnosis	Out of PBM scope – ML model developed for laboratory sickle cell image analysis using pre-existing datasets.
Vittoraki et al. (2020)[464]	Patterns of 1,748 unique human alloimmune responses seen by simple machine learning algorithms	Out of PBM scope – ML applied to HLA antibody response patterns in renal transplant patients.

Waisberg et al. (2022)[465]	A non-invasive approach to monitor anemia during long-duration spaceflight with retinal fundus images and deep learning	Out of PBM scope – focuses on spaceflight-induced anemia; no ML/DL model developed or validated; no real patients.
Wang et al. (2021)[466]	Artificial neural network predicts hemorrhagic contusions following decompressive craniotomy in traumatic brain injury	Out of PBM scope – ANN predicts postoperative intracranial hemorrhage in traumatic brain injury patients (radiological outcome).
Wang et al. (2022)[467]	An interpretable artificial intelligence system for detecting risk factors of gastroesophageal variceal bleeding	Out of PBM scope – DL system stratifies bleeding risk for prophylaxis in cirrhotic patients.
Welsby et al. (2010)[468]	A clinical prediction tool to estimate the number of units of red blood cells needed in primary elective coronary artery bypass surgery.	No ML/DL predictive model used (conventional statistical methods).
Wu et al. (2023)[469]	Machine learning-based model for predicting major adverse cardiovascular and cerebrovascular events in patients aged 65 years and older undergoing noncardiac surgery	Out of PBM scope – ML model predicts cardiovascular/cerebrovascular events; transfusion used only as an input variable, not as an outcome.
Xu et al. (2017)[470]	A deep convolutional neural network for classification of red blood cells in sickle cell anemia	Out of PBM scope – DL model classifies red blood cell morphologies in SCD using microscopy images.
Xu et al. (2020)[471]	Recurrent hemoptysis after bronchial artery embolization: prediction using a nomogram and artificial neural network model	Out of PBM scope – ANN predicts recurrence of hemoptysis after bronchial artery embolization.
Yamakuni et al. (2023)[472]	Prediction of anemia from cerebral venous sinus attenuation on deep-learning reconstructed brain computed tomography images	No ML/DL predictive model developed – DL used only for image reconstruction.
Yang et al. (2021)[473]	Comparison of massive and emergency transfusion prediction scoring systems after trauma with a new bleeding risk index score applied in-flight	No ML/DL model used - algorithmic Bleeding Risk Index based on continuous vital-sign features predicts massive transfusion and critical administration threshold.
Yang et al. (2023)[474]	Blood quality evaluation via on-chip classification of cell morphology using a deep learning algorithm	Out of PBM scope – AI applied to laboratory blood quality evaluation (analytical validation)
Zador et al. (2018)[475]	Multivariable and bayesian network analysis of outcome predictors in acute aneurysmal subarachnoid hemorrhage: review of a pure surgical series in the post-international subarachnoid aneurysm trial era	Out of PBM scope – ML used to identify neurological and functional outcome predictors after aneurysmal subarachnoid hemorrhage.
Zhang et al. (2021)[476]	Evaluation of machine learning-driven automated Kleihauer-Betke counting: a method comparison study	Out of PBM scope – ML applied to laboratory quantification of fetomaternal hemorrhage.
Zhang et al. (2023)[477]	Machine learning model-based risk prediction of severe complications after off-pump coronary artery bypass grafting	Out of PBM scope – ML model predicts general postoperative complications not relevant to PBM.
Zhang et al. (2024)[478]	Enhancing thalassemia gene carrier identification in non-anemic populations using artificial intelligence erythrocyte morphology analysis and machine learning	Out of PBM scope – AI model predicts thalassemia carrier status (genetic screening).

Zhou et al. (2014)[479]	Detection of foreign matter in transfusion solution based on gaussian background modeling and an optimized BP neural network	Out of PBM scope – ML-based computer vision system for detecting foreign matter in transfusion bottles (manufacturing quality control).
Zhu et al. (2021)[480]	Machine learning models predict coagulopathy in spontaneous intracerebral hemorrhage patients in ER	Out of PBM scope – ML models predict coagulopathy in patients with existing intracerebral bleeding (risk of worsening hemorrhage).
Zhu et al. (2021)[481]	Predicting intraventricular hemorrhage growth with a machine learning-based, radiomics-clinical model	Out of PBM scope – ML radiomics-clinical model predicts hematoma expansion in patients with intracerebral hemorrhage.
Zhu et al. (2024)[482]	Enhancing hospital course and outcome prediction in patients with traumatic brain injury: A machine learning study	Out of PBM scope - ML models predict hospital course and general outcomes (admission, mortality, neurosurgery) in traumatic brain injury.
Zürn et al. (2022)[483]	Risk assessment of red cell transfusion in congenital heart disease	No ML/DL model – retrospective analysis using LR and nonparametric statistics.

List of Abbreviations:

PBM Application

AA, Aplastic Anemia; **ACI**, Acute Cerebral Infarction; **ACS**, Acute Coronary Syndrome; **AF**, Atrial Fibrillation; **AIHA**, Autoimmune Hemolytic Anemia; **AIS**, Acute Ischemic Stroke; **AMPS**, Aqueous Multiphase Systems; **bAVM**, Brain Arteriovenous Malformation; **BMI**, Body Mass Index; **CABG**, Coronary Artery Bypass Grafting; **CAD**, Coronary Artery Disease; **CBC**, Complete Blood Count; **COVID-19**, Coronavirus Disease 2019; **CPB**, Cardiopulmonary Bypass; **CRI**, Coagulation Risk Index; **CT**, Computed Tomography; **DM**, Diabetes Mellitus; **DOAC**, Direct Oral Anticoagulant; **EC,** extracorporeal circulation; **ECMO**, Extracorporeal Membrane Oxygenation; **EHRs**, Electronic Health Records; **EKG**, Electrocardiogram; **EPO**, Erythropoietin; **ESA**, Erythropoiesis-Stimulating Agent; **ESD**, Endoscopic Submucosal Dissection; **ESRD**, End-Stage Renal Disease; **ET**, Exchange Transfusion; **GI**, Gastrointestinal; **Hb**, Hemoglobin; **HD**, Hemodialysis; **HER**, Health Electronic Record; **HPA-1**, Human Platelet Antigen-1; **HS**, Hereditary Spherocytosis; **HT**, Hemorrhagic Transformation; **ICH**, Intracerebral Hemorrhage; **ICU**, Intensive Care Unit; **IDA**, Iron Deficiency Anemia; **IARI**, Incomplete Antibody Reaction Intensity; **ITP**, Immune Thrombocytopenic Purpura; **IVH**, Intraventricular Hemorrhage; **MDS**, Myelodysplastic Syndromes; **MIDCAB**, Minimally Invasive Direct Coronary Artery Bypass; **MI**, Myocardial Infarction; **ML**, Machine Learning; **MT**, Massive Transfusion; **NIRS**, Near-Infrared Spectroscopy; **OCT**, Optical Coherence Tomography; **PAS**, Placenta Accreta Spectrum; **PCI**, Percutaneous Coronary Intervention; **PLT**, Platelets; **PPH**, Postpartum Hemorrhage; **PTonH**, Posttonsilectomy Hemorrhage; **RBC**, Red Blood Cell; **RDS**, Respiratory Distress Syndrome; **RETIC**, Reticulocytosis; **Rh**, Rhesus Factor; **SCD**, Sickle Cell Disease; **TACO**, Transfusion-Associated Circulatory Overload; **THA**, Total Hip Arthroplasty; **TKA**, Total Knee Arthroplasty; **TRALI**, Transfusion-Related Acute Lung Injury; **UBIG**, Upper Gastrointestinal Bleeding; **VTE**, Venous Thromboembolism; **VWD**, Von Willebrand Disease; **WES**, Whole Exome Sequence.

Sample Size

AA, Aplastic Anemia; **AIHA**, Autoimmune Hemolytic Anemia; **ANN**, Artificial Neural Network; **Coombs**, Direct Antiglobulin Test; **DEL**, D-EL Phenotype (a weak Rh D variant); **DM**, Diabetes Mellitus; **GI**, Gastrointestinal; **Hb AS**, Heterozygous Sickle Cell Trait; **Hb SS**, Homozygous Sickle Cell Disease; **HER**, Health Electronic Record; **HS**, Hereditary Spherocytosis; **HT**, Hemorrhagic Transformation; **ICH**, Intracerebral Hemorrhage; **IDA**, Iron Deficiency Anemia; **INR**, International Normalized Ratio; **IVH**, Intraventricular Hemorrhage; **MDS**, Myelodysplastic Syndromes; **MT**, Massive Transfusion; **NA**, Not Applicable; **PPH**,

Postpartum Hemorrhage; **PTonH**, Postonsilectomy Hemorrhage; **RBC**, Red Blood Cell; **RETIC**, Reticulocytosis; **RhD**, Rhesus D antigen; **SCD**, Sickle Cell Disease; **Treatment group assignment (1:1:1 vs. 1:1:2)**, equal units of plasma, platelets and red blood cells vs. equal plasma and platelets, but twice as many red blood cells as plasma or platelets; **VWD**, Von Willebrand Disease.

Model Variables/Top Predictors

ABI, Ankle-Brachial Index; **ABG**, Arterial Blood Gas; **ABO**, ABO blood group system; **ABS**, Assessment of Blood Consumption Score; **ACE**, Angiotensin-Converting Enzyme; **ACS**, Acute Coronary Syndrome; **ACT**, Activated Clotting Time; **AF**, Atrial Fibrillation; **AFP**, Alpha-fetoprotein; **AG**, Anion Gap; **AHTR**, Acute Hemolytic Transfusion Reaction; **AIDS**, Acquired Immune Dificiency Syndrome; **AIS**, Abbreviated Injury Scale; **ALB**, Albumin; **ALB/GLO**, albumin-to-globulin ratio; **ALP**, Alkaline Phosphatase; **ALT**, Aspartate aminotransferase; **AMPS**, Aqueous Multiphase Systems; **ANC**, Antenatal Care; **Anti-D**, Anti-D Immunoglobulin; **APGAR,** clinical assessment tool used for newborns - Appearance, Pulse, Grimace, Activity, and Respiration; **α-HBDH,** α-hydroxybutyrate dehydrogenase; **ApoA1**, apolipoprotein A1; **ApoB**, apolipoprotein B; **APTTR**, Activated Partial Thromboplastin Time Ratio; **APTT**, Activated Partial Thromboplastin Time; **ARB**, Angiotensin II Receptor Blocker; **ART**, **Assisted Reproductive Technology; ASA**, American Society of Anesthesiologists physical status classification system; **ASPECTS**, Alberta Stroke Program Early CT Score; **AST**, Aspartate Aminotransferase; **AUAW**, **Area Under the Arterial Waveform** per cardiac beat; **AVR**, Aortic Valve Replacement; **BASO**, Basophil; **bAVM**, Brain Arteriovenous Malformation; **BD**, Base Deficit; **BE**, Base Excess; **β-hCG, beta-human chorionic gonadotropin; bil**, bilirubin; **BMI**, Body Mass Index; **BP**, Blood Pressure; **BSA**, Body Surface Area; **BUN**, Blood Urea Nitrogen; **C3**, Complement C3; **C3d**, Complement Component 3d; **C4**, Complement C4; **Ca**, Calcium; **CA 19-9**, Carbohydrate Antigen 19-9; **CA-125**, Carbohydrate antigen-125; **CA-153**, Carbohydrate antigen-153; **CA-199**, Carbohydrate antigen-199; **CABG**, Coronary Artery Bypass Grafting; **CAD**, Coronary Artery Disease; **Cath**, Cardiac Catheterization; **CBOW**, Continuous Bag-of-Words; **CCI**, Charlson Comorbidity Index; **CCM**, Cerebral Cavernous Malformations; **CCS**, Canadian Cardiovascular Society; **CEA**, Carcinoembryonic antigen; **CHD**, Coronary heart disease; **ChE**, Cholinesterase; **CHF**, Congestive Heart Failure; **CIELAB**, Commission Internationale de l´Éclairage Lab or CIE **L*a* b*** represent color space; **CK**, Creatinine Kinase; **CKD**, Chronic Kidney Disease; **CK-MB**, Creatinine Kinase MB; **Cl**, Chloride; **ClCr**, Creatinine clearance; **CMOS**, Complementary Metal-Oxide-Semiconductor; **CNN**, Convolutional Neural Network; **CO_2**, Carbon Dioxide; **COPD**, Chronic Obstructive Pulmonary Disease; **COVID-19**, Coronavirus Disease 2019; **CPB**, Cardiopulmonary Bypass; **CRP**, C-Reactive Protein; **CT**, Computed Tomography; **CVD**, Cerebrovascular Disease**; Cyfra21-1**, human CYFRA21-I antigen; **CysC**, cystatin C; **DAPT**, Dual Antiplatelet Therapy; **Dbil**, Direct bilirubin; **DBP**, Diastolic Blood Pressure; **DD**, D-dimer; **DDAVP**, Desmopressin; **DHTR**, Delayed Hemolytic Transfusion Reaction; **DIC**, Disseminated Intravascular Coagulation; **DL**, Deep Learning; **DM**, Diabetes Mellitus; **DOAC**, Direct Oral Anticoagulant; **DSO**, Discocytes + Stomatocytes + Oval; **DSTR**, Delayed Serologic Transfusion Reaction; **DVT**, Deep Venous Thrombosis; **EC**, extracorporeal circulation; **ECMO**,

Extracorporeal Membrane Oxygenation; **ECOG**, Eastern Cooperative Oncology Group; **ED**, Emergency Department; **eFAST**, Extended Focused Assessment with Sonography for Trauma; **EGD**, Esophagogastroduodenoscopy; **eGFR**, estimated Glomerular Filtration Rate**; EHRs**, Electronic Health Records; **EKG**, Electrocardiogram; **eKt/V**, estimate Kt/V (K=rea clearance, t=treatment time, V=urea volume distribution); **EOS**, Eosinophil; **ER**, Emergency Room; **ESR**, Erythrocyte Sedimentation Rate; **ESA**, Erythropoiesis-Stimulating Agent; **ET**, Exchange Transfusion; **FAST**, Focused Assessment with Sonography in Trauma; **Fe**, Iron; **FFP**, Fresh frozen plasma**; FIB**, Fibrinogen; **FIB-4**, Fibrosis-4 Index; **FiO_2**, Fraction (or percentage) of oxygen; **FT3**, free triiodothyronine **FT4**; free thyroxine; **G6PD**, Glucose-6-Phosphate Dehydrogenase; **GCT**, Gel Card Technique; **GDM**, Gestational Diabetes Mellitus; **GGT**, Gamma-Glutamyl Transferase; **GHT**, Gestational Hypertension; **GI**, Gastrointestinal; *GLO, Globulin;* **GCS**, Glasgow Coma Scale; **^1H**, isotype of hydrogen with one proton; **H1**, *H1 Receptor; H2, H-2 Receptor;* **Hb**, Hemoglobin; *HbA0, Adult Hemoglobin; HbA1C, a form of glycated Hemoglobin; HbA2, minor adult Hemoglobin variant; HbF, Fetal Hemoglobin;* **HbsAb**, Hepatitis B surface antibody; **HBsAg**, Hepatitis B surface antigen; **HBV**, Hepatitis B Virus; **HCV**, Hepatitis C Virus; **HCVAb**, Hepatitis C virus antibody; **HCT**, Hematocrit; **HD**, Hemodialysis; **HDL**, high-density lipoprotein; **HF**, Heart Failure; **HIS**, Hue-Saturation-Intensity color model; **HPA-1**, Human Platelet Antigen-1; **HR**, Heart Rate; **HSCRP**, High Sensitivity C Reactive Protein; **hs-cTnT**, high-sensitivity cardiac troponin T; **HSV**, Hue, Saturation, Value; **HTR**, Hypotensive Transfusion reaction; **HUmax**, maximum Hounsfield unit; ; **IARI**, Incomplete Antibody Reaction Intensity; **Ibil**, Indirect bilirubin; **ICD-10**, International Classification of Diseases, 10th Revision; **ICH**, Intracerebral Hemorrhage; **ICU**, Intensive Care Unit; **Ig**, Immunoglobulin; *IgA*, Immunoglobulin A; *IgG*, Immunoglobulin G; *IgM*, Immunoglobulin M; *IHD, Ischemic Heart Disease;* **IL-6**, Interleukin-6; **INR**, International Normalized Ratio; **IPMN**, Intraductal Papillary Mucinous Neoplasm; **ISS**, Injury Severity Score; **ITP**, Immune Thrombocytopenic Purpura; **IUD**, Intrauterine Device; **IUFD**, Intrauterine Fetal Death; **IVC**, Inferior Vena Cava; *IVF,* In Vitro Fertilization; **IVH**, Intraventricular Hemorrhage; **K**, Potassium; **LBP**, Local Binary Patterns; **LDH**, Lactate dehydrogenase; **LDL**, low-density lipoprotein; **LMR, lymphocyte-to-monocyte ratio; LOS, Length of stay; LRRBC**, Leukoreduced Red Blood Cells; **LVEF**, Left Ventricular Ejection Fraction; **LYM**, Lymphocyte; **MAP**, Mean Arterial Pressure; **MCH**, Mean Corpuscular Hemoglobin; **MCHC**, Mean Corpuscular Hemoglobin Concentration; **MCV**, Mean Corpuscular Volume; **MELD**, Model for End-Stage Liver Disease Score; **MFI**, Modified Frailty Index; **Mg**, Magnesium; **MI**, Myocardial Infarction; **mIEF**, Microstrip Isoelectric focusing; **MO**, Monocyte; **MO-BGWO**, Multi-Objective Binary Grey Wolf Optimization; **MPV**, Mean Platelet Volume; **MRI**, Magnetic Resonance Imaging; **MSBOS**, Maximum Surgical Blood Ordering Schedule; **MT**, Massive Transfusion; **Na**, Sodium; **NCCT**, Noncontrast computed tomography; **NET**, Neuroendocrine Tumor; **NEU**, Neutrophils; **NIDBP**, Non-Invasive Mean Blood Pressure; **NIHSS**, National Institute of Health Stroke Scale; **NIMBP**, Non-Invasive Mean Blood Pressure; **NISBP**, Non-Invasive Systolic Blood Pressure; **NISS**, New Injury Severity Score; **NLP**, Natural Language Processing; **NLR**, neutrophil-to-lymphocyte ratio; **NMR**, **Nuclear Magnetic Resonance**; **NSAID**, Non-Steroidal Anti-Inflammatory; **NSTEMI**, **Non-ST-Elevation Myocardial Infarction; NT-proBNP**, N-terminal pro-B-type Natriuretic Peptide; **NYHA**, New York Heart

Association functional classification; **O_2Hb**, Oxyhemoglobin; **OcmKt/V**, Online Clearance Monitoring Kt/V; **OCT**, Optical Coherence Tomography; **P**, Phosphorous; **$P2Y_{12}RA$**, $P2Y_{12}$ Receptor Antagonist; **$PaCO_2$**, Partial pressure of carbon dioxide; **PAD**, Peripheral Arterial Disease; **PaO_2**, partial pressure of oxygen; **PAS**, Placenta Accreta Spectrum; **PCI**, Percutaneous Coronary Intervention; **PCT**, Platelecrit; **PCV**, Packed Cell Volume; **PDA**, Patent Ductus Arteriosus; **PDAC**, Pancreatic Adenocarcinoma; **PDW**, Platelet Distribution With; **PE**, Pulmonary Embolism; **pH**, potential of Hydrogen; **PLR**, Platelet-to-Lymphocyte Ratio; **PLT**, Platelet; **PNI**, Prognostic Nutritional Index; *PPG,* **Photoplethysmography**; **PPH**, Postpartum Hemorrhage; **PPI**, Proton-pump inhibitors; **Pre-BNP**, B-type Natriuretic Peptide Precursor; **PT**, Prothrombin Time; **PTA**, Prothrombin Activity; **PTCA**, Percutaneous Transluminal Coronary Angioplasty; **PTH**, Parathyroid Hormone; **PTP**, Post-transfusion Purpura; **PVD**, Peripheral Vascular Disease; **RBC**, Red Blood Cell; **RDS**, Respiratory Distress Syndrome; **RDW**, Red Blood Cell Distribution Width; **RDW-CV**, Red Cell Distribution Width – Coefficient of Variation; **RDW-SD,** Red Cell Distribution Width – Standard Deviation; **Retic**, Reticulocyte Count; **RGB**, RBG channels that represent the three primary color components—Red (R), Green (G), and Blue (B); **RGE**, Reticulocytes + Granular + Echinocytes; **Rh**, Rhesus factor; **RHCE**, Rhesus C/c and E/e Antigen Gene; **RhD**, Rhesus D antigen; **RHD**, Rh D Antigen Gene; **RLym**; Reactive Lymphocytes; **RR**, Respiratory Rate; **R-time**, Reaction Time; **rtPA**, Recombinant tissue Plasminogen Activator; **SAH**, Systemic Arterial Hypertension; **SBP**, Systolic blood pressure; **SCC Ag**, Squamous Cell Carcinoma antigen; **SI**, Shock Index; **SDRDW**, Standard Deviation of Red Blood Cell Distribution With; **SDTSD**, Standard Deviation of Platelet Distribution Width; **SIPA**, Shock Index Pediatric-Adjusted; **SLE**, Systemic Lupus Erythematosus; **SOFA**, Sepsis-related Organ Failure Assessment; **spKt/V**, single pool Kt/V; **SpO_2**, arterial oxygen saturation; **STD**, Sexually Transmitted Disease; **STEMI**, ST-Elevation Myocardial Infarction; **SSRI**, Selective Serotonin Reuptake Inhibitor; **T3**, triiodothyronine; **T4**, thyroxine; **TACO**, Transfusion-Associated Circulatory Overload; **TAD**, Transfusion-Associated Dyspnea; **TA-GVHD**, Transfusion-Associated Graft-Versus-Host Disease; **Tbil**, Total bilirubin; **TCO_2**, Total Carbon Dioxide; **TEG**, Thromboelastography parameters; **TG**, Triglyceride; **TIA**, Transient Ischemic Attack; **TIBC**, Total Iron-Binding Capacity; **TIMI**, Thrombolysis In Myocardial Infarction; **TLOC**, **Transitory Loss of Consciousness; TNM**, Tumor, Nodes, Metastasis staging system; **TOAST**, Trial of ORG 10172 in Acute Stroke Treatment; **TOLAC**, Trial of Labor after Cesarean; **TP**, Total Protein; **tPA**, tissue Plasminogen Activator; **TPO-RA**, Thrombopoietin Receptor Agonist; **TRALI**, Transfusion-Related Acute Lung Injury; **Treatment group assignment (1:1:1 vs. 1:1:2)**, equal units of plasma, platelets and red blood cells vs. equal plasma and platelets, but twice as many red blood cells as plasma or platelets; **TRF**, Transferrin; **TSAT**, Transferrin Saturation; **TSD**, Mean of Standard Deviation for Platelet and RBC Width; **TSH**, Thyroid-Stimulating Hormone; **TT**, Thrombin Time; **TyG**, triglyceride-glucose index; **TTI**, Transfusion-Transmitted Infection; **TXA**, Tranexamic Acid; **UGIB**, Upper Gastrointestinal Bleeding; **UIBC**, Unsaturated Iron-Binding Capacity; **UICC**, Union for International Cancer Control; **US**, Ultrasound; **UTI**, Urinary Tract Infection; **VHD**, Valvular Heart Disease; **VitA**, Vitamin A; **VitB12**, Vitamin B12; **VitD**, Vitamin D; **VTE**, Venous Thromboembolism; **VWF**, Von Willebrand Factor; **WBC**, White Blood Cell.

Models

AdaBoost, Adaptive Boosting; **AI-BR**, Artificial Intelligence Bleeding Risk Model; **ANFIS**, Adaptive Neuro-Fuzzy Inference System; **ANN**, Artificial Neural Network); **ARIMA**, Autoregressive Integrated Moving Average; **ARX**, Auto-Regressive with Exogenous inputs; **ARX-3**, Autoregressive Model with Exogenous Inputs (Order 3); **Bagging DT**, Bagging Decision Tree; **BBN**, Bayesian Belief Networks; **BaggingC**, Bagging Classifier; **B-CART**, Bagged Classification And Regression Trees; **BCT**, Boosted Classification Tree; **BDL**, Baseline Deep Learning; **BERT**, Bidirectional-Encoder Representations-Transformers; **Beta TC VAE,** Beta Total Correlation Variational Autoencoder**; BNB**, Bernoulli Naïve Bayes; **BN**, Bayesian Network; **BPANet**, Body-Part_anemia Network; **BR**, Bayesian Ridge; **BRF,** Balanced Random Forest; **BRR**, Bayesian Ridge Regression; **BT**, Boosted Tree; **BTR**, Boosting Tree Regression; **CART**, Classification and Regression Tree analysis; **CatBoost**, Categorical Boosting; **CBAM,** Convolutional Block Attention Module; **C-CFI**, Clinical-Coagulation Functional Indexes; **CFI**, Coagulation Function Indexes; **CoarseT**, Coarse Tree; **C-R**, Clinical-Radiomics; **C-R-CFI**, Clinical-Radiomics-Coagulation Functional Indexes; **CFN**, Cascade Forward Network; **CIRF**, Conditional Inference Random Forest; **CIT**, Conditional Inference Tree; **CNN**, Convolutional Neural Network; **CRF**, Conditional Random Fields; **CSA**, Crow Search Algorithm; **CSO**, Chicken Swarm Optimization Algorithm; **CTree**, Classification Tree; **cVAE**, Clinical Variational Autoencoder; **DAE**, Denoising Autoencoder; **DDN**, Distributed Delay Network; **DDOD**, Disentangled Dense Object Detector; **DDQN**, Double Deep Q-Network; **DDQN-PER**, Deep Q-Network Prioritized Experienced Replay; **DL**, Deep Learning; **DLNN**, Deep Learning Neural Network; **DNN**, Deep Neural Network; **DNN-**A, Deep Neural Network with an attention mechanism; **DNN-IAD,** Dirichlet Deep Neural Network; **DQN**, Deep Q-Network; **DQN-PER**, Deep Q-Network Prioritized Experienced Replay; **DRL**, Deep Reinforcement Learning; **DT**, Decision Trees; **DTC**, Decision Tree Classifier; **DTNB**, Decision Table–Naive Bayes Hybrid; **DTR**, Decision Tree Regression; **EL-HC / EL-SC** – Ensemble Learning (Hard/Soft Clustering); **Emb**, Learned Embedding; **ELM**, Extreme Learning Machine; **ELR**, **Extreme Logistic Regression**; **EN**, Elastic Net; **ENN**, Elman Neural Network; **ENLR**, Elastic Net Logistic Regression; ENP, Elastic-net penalized logistic regression; **ENPLR**, Elastic-net Penalized Logistic Regression; **ERT,** Extremely Randomized Trees; **ET**, Extra Trees; **ETC**, Extra Trees Classifier; **ETR**, Extra Trees Regressor; **EVC**, Ensemble Voting Classifier; **FDA**, Flexible Discriminant Analysis; **FIR**, Finite Impulse Response Network; **FFN**, Feedforward Network; **FFNN**, Feedforward Neural Network; **GAM**, Generalized Additive Model; **GaussianP**, Gaussian Process Classification and Regression (hybrid model); **GB,** Gradient Boosting; **GBC**, Gradient Boosting Classifier; **GBDT**, Gradient Boosting Decision Tree; **GBM**, Gradient Boosting Machine; **GBR**, Gradient Boosting Regression; **GBT**, Gradient Boosting Tree; **GLM**, Generalized Linear Model; **GNB**, Gaussian Naïve Bayes; **GP**, genetic programming with offspring selection; **GPR**, Gaussian Process Regression; **GRU**, Gated Recurrent Unit Networks; **GRU-AM**, Gated Recurrent Unit with Attention Mechanism; **GRU-D**, Gated Recurrent Unit with Decay; **GRU-GNL**, Gated Recurrent Unit with Gaussian Noise Layer; **GRU-RNN**, Gated Recurrent Network–Recurrent Neural Network; **HGB**, Histogram-Based Gradient Boosting; **HuberR**, Huber

Regression; **KNN**, K-Nearest Neighbors; **KNNR**, KNN Regression; **LASSO**, Least Absolute Shrinkage and Selection Operator; **LASSOR**, Lasso Regression; **LDA**, Linear Discriminant Analysis; **LinearR**, Linear Regression; **LGB**, Light Gradient Boost Machine; **LGBM**, Light Gradient Boosting Machine; **LGBMR**, Light Gradient Boosting Machine Regressor; **LinearSVC**, Linear Support Vector Classification; **LM-LSTM-CRF**, Language Model Long Short-Term Memory Conditional Random Field; **LMT**, Logistic Model Tree; **LPBoost**, Linear Programming Boost; **LR**, Logistic Regression; **LREN**, Logistic Regression and Elastic Net; **LSTM**, Long Short-Term Memory Networks; **LVQ**, Learning Vector Quantization; **MAML**, Model-Agnostic Meta-Learning; **MARS**, Multivariate Adaptive Regression Splines; **MGSVM**, Medium Gaussian Support Vector Machine; **ML**, Machine Learning; **MLP**, Multilayer Perceptron; **MLPC**, Multlayer Perceptron Classifier; **MLPNN**, Multilayer Perceptron Neural Network; **MLR**, Multivariate Logistic Regression; **MLinearR**, Multiple Linear Regression; **MM**, Multimodal Model; **MMoE**, Multi-Gate Mixture of Experts; **MobileNetV3+SE**, MobileNetV3 architecture used with Squeeze-and-Excitation blocks; **MPNN**, Multilayer Perceptron Neural Network; **MR**, Multiple Regression; **MTNN**, Multi-Task Neural Network; **MV**, Majority Voting; **NARX**, Neural Network Auto-Regressive with Exogenous inputs; **NB**, Naïve Bayes; **NLP**, Natural Language Processing; **NN**, Neural Networks; **NN-OSRE**, Neural Network – Orthogonal Search-Based Rule Extraction; **OBCSA-OSAE**, Oppositional Binary Crow Search Algorithm + Optimal Stacked Autoencoder Model; OneR, 1-rule classifier; **PBNN**, Pruning Bayesian Neural Network; **PCA**, Principal Component Analysis; **PD-SVR**, Profile-Dependent Support Vector Regression; **PFI**, Permutation Feature Importance; **PINN**, Physics-Informed Neural Network; **PN**, Prototypical Network; **PNN**, Probabilistic Neural Network; **PR**, Poisson Regression; **P-W CS**, Patch-Wise Crumpled State model; **QDA**, Quadrant Discriminant Analysis; **RANSAC**, Random Sample Consensus; **RBC**, Red Blood Cell; **RBF**, Radial Basis Function Neural Networks; **RBM**, Restricted Boltzmann Machines; **R-CFI**, Radiomics- Coagulation Function Indexes; **R-CNN**, Region-based Convolutional Neural Network; **RELM**, Regularized Extreme Learning Machine; **RegCOX**, Regularized Cox Regression; **ResNet**, Residual Network; **RF**, Random Forest**; RFR**, Random Forest Regression**; rKNN**, Random K-Nearest Neighbors; **RNN**, Recurrent Neural Network; **RSF**, Random Survival Forest; **RReg**, Ridge Regression; **RT**, Regression Tree; **RuleBC**, Rule Based Chamber; **RUSBoost**, Random-Undersampling Boost; **SGD**, Stochastic Gradient Descent; **SGDR**, Stochastic Gradient Descent Regression; **SGM**, Stacked Generalization Model (ensemble of multiple classifiers); **SLDA**, Sparse Linear Discriminant Analysis; **SFN,** Supervised Feed-Forward Neural Networks; **SNF-FIQ**, Supervised Feed-forward Neural Network with Fuzzy Interquartile Encoding; **SGBT,** Stochastic Gradient Boosting; **SL**, Super Learner ensemble model; **SLIC-GAT**, Simple Linear Iterative Clustering- Graph Attention Network; **SLR**, Stepwise Logistic Regression; **SMO**, Sequential Minimal Optimization; **SMOTE**, Synthetic Minority Over-sampling Technique; **SMOTE-MRS**, Synthetic Minority Over-sampling Technique – Multiresolution Sampling Technique; **STL**, Seasonal and Trend decomposition using Loess; **surgVAE,** Surgical Variational Autoencoder; **SVC**, Support Vector Classifier; **SVM**, Support Vector Machine; **SVR**, Support

Vector Regression; **TDN**, Time Delay Network; **TFIM**, TensorFlow Imbalanced; **TMLE**, Targeted Maximum Likelihood Estimation; **TRALI**, Transfusion-Related Acute Lung Injury; **URF**, Unsupervised Random Forest; **VAE,** Variational Autoencoder; **VC**, Voting Classifier; **ViT,** Vision Transformer; **WSDL**, Weaky Supervised Deep Learning; **XGB**, eXtreme Gradient Boosting; **XGBT**, eXtreme Gradient Boosting Tree.

Model Validation

ANN, Artificial Neural Network; **CV**, Cross-Validation; **DL**, Deep Learning; **EV**, External Validation; **KNN**, K-Nearest Neighbors; **LASSO**, Least Absolute Shrinkage and Selection Operator; **LSTM**, Long Short-Term Memory; **n**, Sample Size; **RBC**, Red Blood Cell; **RFE**, Recursive Feature Elimination; **RF**, Random Forest; **SVM**, Support Vector Machine; **Var**, Varices; **XGB**, eXtreme Gradient Boosting.

Key Findings

ACC-BR, American College of Cardiology CathPCI bleeding risk**; ACM, Anemia Control Model; AdaBoost,** Adaptive Boosting; **AHTR**, Acute Hemolytic Transfusion Reaction; **AI-BR**, Artificial Intelligence Bleeding Risk; **AMPS**, Aqueous Multiphase Systems; **ANN**, Artificial Neural Network; **ARIMA**, Autoregressive Integrated Moving Average; **AUC**, Area Under the Receiver Operating Characteristic Curve; **AUC-PR**, Area Under the Precision-Recall Curve; **BBN**, Bayesian Belief Networks; **BPANet**, Body-Part_anemia Network; **CART**, Classification and Regression Tree analysis; **CatBoost**, Categorical Boosting; **CBAM,** Convolutional Block Attention Module**; CI**, Confidence Interval; **CIRF**, Conditional Inference Random Forest; **CIT**, Conditional Inference Tree; **CNN**, Convolutional Neural Network; **CPB**, Cardiopulmonary Bypass; **CSA**, Crow Search Algorithm; **CT**, Computed Tomography; **DAE**, Denoising Autoencoder; **DDN**, Distributed Delay Network; **DDOD**, Disentangled Dense Object Detector; **DDQN-PER**, Deep Q-Network Prioritized Experienced Replay; **DHTR**, Delayed Hemolytic Transfusion Reaction; **DL**, Deep Learning; **DNN**, Deep Neural Network; **DNN-**A, Deep Neural Network with an attention mechanism; **DNN-IAD,** Dirichlet Deep Neural Network; **DOAC**, Direct Oral Anticoagulant; **DT**, Decision Trees; **ELM**, Extreme Learning Machine; **EN**, Elastic Net; **ENN**, Elman Neural Network; **ENPLR**, Elastic-net Penalized Logistic Regression; **EPO**, Erythropoietin; **ESA**, Erythropoiesis-Stimulating Agent; **ETC**, Extra Trees Classifier**; EV**, External Validation; **EVC**, Ensemble Voting Classifier; **EVS**, Explained Variance Score; **FFP**, Fresh frozen plasma**; FIB**, Fibrinogen; **GaussianP**, Gaussian Process Classification and Regression (hybrid model); **GB,** Gradient Boosting; **GBC**, Gradient Boosting Classifier; **GBDT**, Gradient Boosting Decision Tree; **GBM**, Gradient Boosting Machine; **GBT**, Gradient Boosting Tree; **GRASPS, score to predict risk of intracranial bleeding; GRU**, Gated Recurrent Unit Networks; **GRU-AM**, Gated Recurrent Unit with Attention Mechanism; **GRU-RNN**, Gated Recurrent Network – Recurrent Neural Network; **Hb, Hemoglobin; HD**, Hemodialysis; **ICH**, Intracerebral Hemorrhage; **ICU**, Intensive Care Unit; **IDA, Iron Deficiency Anemia; IR**, Interventional Radiology cohort; **KNN**, K-Nearest Neighbors; **LASSO**, Least Absolute Shrinkage and Selection Operator; **LASSOR**, Lasso Regression; **LGBM**, Light Gradient Boosting Machine; **LDA**, Linear Discriminant Analysis; **LinearR**, Linear Regression; **LR**,

Logistic Regression; **LSTM**, Long Short-Term Memory Networks; **LRRBC**, Leukoreduced Red Blood Cells; **MACE**, Major Averse Cardiovascular Events; **MAE**, Mean Absolute Error; **mAP@0.5, Mean Average Precision** at an **Intersection over union threshold of 0.5; MAPE**, Mean Absolute Percentage Error; **MARS**, Multivariate Adaptive Regression Splines; **MBT**, Massive Blood Transfusion; **MCV**, Mean Corpuscular Volume; **MCH**, Mean Corpuscular Hemoglobin; **MDS**, Myelodysplastic Syndromes; **MGSVM**, Medium Gaussian Support Vector Machine; **mIEF**, Microstrip Isoelectric focusing; **ML**, Machine Learning; **MLinearR**, Multiple Linear Regression; **MLP**, Multilayer Perceptron; **MLPNN**, Multilayer Perceptron Neural Network; **MLR**, Multivariate Logistic Regression; **MO-BGWO**, Multi-Objective Binary Grey Wolf Optimization; **MobileNetV3+SE**, MobileNetV3 architecture used with Squeeze-and-Excitation blocks; **MPNN**, Multilayer Perceptron Neural Network; **MSE**, Mean Square Error; **MSS, score to predict intracranial hemorrhage risk; MT**, Massive Transfusion; **NB**, Naïve Bayes; **NCDR**, National Cardiovascular Data Registry; **NCS**, Non-Cardiac Surgery cohort; **NIRS**, Near-Infrared Spectroscopy; **NN**, Neural Network; **NN-OSRE**, Neural Network – Orthogonal Search-Based Rule Extraction; **NPV**, Negative Predictive Value; **OBCSA-OSAE**, Oppositional Binary Crow Search Algorithm + Optimal Stacked Autoencoder Model; **PCA**, Principal Component Analysis; **PCC**, Pearson's Correlation Coefficient; ; **PCI**, Percutaneous Coronary Intervention; **PD-SVR**, Profile-Dependent Support Vector Regression; **PINN**, Physics-Informed Neural Network; **PLT**, Platelets; **PNN**, Probabilistic Neural Network; **PPV**, Positive Predictive Value; **PT**, prothrombin time; **QDA**, Quadrant Discriminant Analysis; **RBC**, Red Blood Cell; **RBF**, Radial Basis Function Neural Networks; **RBM**, Restricted Boltzmann Machines; **RBNLP**, Rules-based Natural Language Processing; **RegCOX**, Regularized Cox Regression; **RELM**, Regularized Extreme Learning Machine; **RF**, Random Forest; **RHCE**, Rhesus C/c and E/e Antigen Gene; **RHD**, Rh D Antigen Gene; **RHHA**, Rare Hereditary Hemolytic Anemia; **RMSE**, Root Mean Square Error; **ROIs**, Regions of Interest from the image; **RR**, Relative Risk; **RUSBoost**, Random-Undersampling Boost; **SCD**, Sickle Cell Disease; **SD**, Standard Deviation; **SEDAN, score to estimate risk of symptomatic intracerebral hemorrhage; SL**, Super Learner ensemble model; **SNF-FIQ**, Supervised Feed-forward Neural Network with Fuzzy Interquartile Encoding; **SHAP**, SHapley Additive exPlanations; **SL**, Super Learner ensemble model; **SLIC-GAT**, Simple Linear Iterative Clustering- Graph Attention Network; **SMO**, Sequential Minimal Optimization; **SMOTE**, Synthetic Minority Over-sampling Technique; **SMOTE-MRS**, Synthetic Minority Over-sampling Technique – Multiresolution Sampling Technique; **SNF-FIQ**, Supervised Feed-forward Neural Network with Fuzzy Interquartile Encoding; **STL**, Seasonal and Trend decomposition using Loess; **SVM**, Support Vector Machine; **THA**, total hip arthroplasty; **TM**, Transfusion Medicine; **TMLE**, Targeted Maximum Likelihood Estimation; **URF**, Unsupervised Random Forest; **VC**, Voting Classifier; **ViT,** Vision Transformer; **VWD**, Von Willebrand Disease; **WES**, Whole-Exome Sequencing; **WGS**, Whole-Genome Sequencing; **WSDL**, Weaky Supervised Deep Learning; **XGB**, eXtreme Gradient Boosting; **XGBT**, eXtreme Gradient Boosting Tree.

PRISMA Checklist

Section and Topic	Item #	Checklist item	Location where item is reported
**TITLE**			
Title	1	Identify the report as a systematic review.	Title page – identified as 'Systematic Review' (p. 1)
**ABSTRACT**			
Abstract	2	See the PRISMA 2020 for Abstracts checklist.	Abstract – structured summary of background, objectives, methods, results, and conclusions (p. 1)
**INTRODUCTION**			
Rationale	3	Describe the rationale for the review in the context of existing knowledge.	Section 1 – Introduction (p. 2)
Objectives	4	Provide an explicit statement of the objective(s) or question(s) the review addresses.	Section 1.3 – Aims and Objectives (p. 3)
**METHODS**			
Eligibility criteria	5	Specify the inclusion and exclusion criteria for the review and how studies were grouped for the syntheses.	Section 2.1 – Eligibility Criteria (p. 4)
Information sources	6	Specify all databases, registers, websites, organisations, reference lists and other sources searched or consulted to identify studies. Specify the date when each source was last searched or consulted.	Section 2.2 – Search Strategy (p. 4)
Search strategy	7	Present the full search strategies for all databases, registers and websites, including any filters and limits used.	Section 2.2 – Full search queries described for PubMed, Scopus, Web of Science (p. 4)
Selection process	8	Specify the methods used to decide whether a study met the inclusion criteria of the review, including how many reviewers screened each record and each report retrieved, whether they worked independently, and if applicable, details of automation tools used in the process.	Section 2.3 – Study Selection; Figure 1 – PRISMA flow diagram (p. 4–5)
Data collection process	9	Specify the methods used to collect data from reports, including how many reviewers collected data from each report, whether they worked independently, any processes for obtaining or confirming data from study investigators, and if applicable, details of automation tools used in the process.	Section 2.4 – Data Collection Process (p. 5)
Data items	10a	List and define all outcomes for which data were sought. Specify whether all results that were compatible with each outcome domain in each study were sought (e.g. for all measures, time points, analyses), and if not, the methods used to decide which results to collect.	Section 2.4 – Data items (outcomes, predictors, performance metrics) (p. 5)
	10b	List and define all other variables for which data were sought	Section 2.4 – Data items (variables, assumptions about missing data) (p. 5)

Section and Topic	Item #	Checklist item	Location where item is reported
		(e.g. participant and intervention characteristics, funding sources). Describe any assumptions made about any missing or unclear information.	
Study risk of bias assessment	11	Specify the methods used to assess risk of bias in the included studies, including details of the tool(s) used, how many reviewers assessed each study and whether they worked independently, and if applicable, details of automation tools used in the process.	Section 2.5 – Risk of Bias assessment (p. 5)
Effect measures	12	Specify for each outcome the effect measure(s) (e.g. risk ratio, mean difference) used in the synthesis or presentation of results.	Section 2.6 – Narrative synthesis; no meta-analysis performed (p. 5)
Synthesis methods	13a	Describe the processes used to decide which studies were eligible for each synthesis (e.g. tabulating the study intervention characteristics and comparing against the planned groups for each synthesis (item #5)).	Section 2.6 – Grouping of studies by PBM domain (p. 5)
	13b	Describe any methods required to prepare the data for presentation or synthesis, such as handling of missing summary statistics, or data conversions.	Section 2.6 – Data preparation (handling duplicates, selecting best-performing models) (p. 5)
	13c	Describe any methods used to tabulate or visually display results of individual studies and syntheses.	Section 2.2 and 2.6 Tabulation of study characteristics and results; Supplementary Tables S1–S5 (p. 4–5)
	13d	Describe any methods used to synthesize results and provide a rationale for the choice(s). If meta-analysis was performed, describe the model(s), method(s) to identify the presence and extent of statistical heterogeneity, and software package(s) used.	Not applicable – no quantitative synthesis or meta - analysis performed (p. 4–5)
	13e	Describe any methods used to explore possible causes of heterogeneity among study results (e.g. subgroup analysis, meta-regression).	Not applicable – no quantitative heterogeneity analysis performed (p. 4–5)
	13f	Describe any sensitivity analyses conducted to assess robustness of the synthesized results.	Not applicable – no sensitivity analysis conducted (p. 4–5)
Reporting bias assessment	14	Describe any methods used to assess risk of bias due to missing results in a synthesis (arising from reporting biases).	Section 2.5 – Qualitative assessment (p.5); Discussion – mentions reporting bias limitations (11-12)
Certainty assessment	15	Describe any methods used to assess certainty (or confidence) in the body of evidence for an outcome.	Section 2.5 and Discussion – Certainty qualitatively addressed (p. 5, 11–12)
**RESULTS**			

Section and Topic	Item #	Checklist item	Location where item is reported
Study selection	16a	Describe the results of the search and selection process, from the number of records identified in the search to the number of studies included in the review, ideally using a flow diagram.	Section 2.3 – Study selection results; Figure 1 – PRISMA flow diagram (p. 4–5)
	16b	Cite studies that might appear to meet the inclusion criteria, but which were excluded, and explain why they were excluded.	Supplementary Table S5 – Excluded studies with reasons (p. 5)
Study characteristics	17	Cite each included study and present its characteristics.	Results – Sections 3.1–3.5 and Supplementary Tables S1–S4 (p. 6–8)
Risk of bias in studies	18	Present assessments of risk of bias for each included study.	Section 2.5 – Risk of bias qualitative summary; Results overview (p. 5, 6-11)
Results of individual studies	19	For all outcomes, present, for each study: (a) summary statistics for each group (where appropriate) and (b) an effect estimate and its precision (e.g. confidence/credible interval), ideally using structured tables or plots.	Results – Sections 3.1–3.5 (domain-level synthesis, representative studies summarized) (p. 6–8)
Results of syntheses	20a	For each synthesis, briefly summarise the characteristics and risk of bias among contributing studies.	Section 3.6 – Comparative distribution of model performance (p. 8-11)
	20b	Present results of all statistical syntheses conducted. If meta-analysis was done, present for each the summary estimate and its precision (e.g. confidence/credible interval) and measures of statistical heterogeneity. If comparing groups, describe the direction of the effect.	Not applicable – no meta-analysis conducted (p. 5)
	20c	Present results of all investigations of possible causes of heterogeneity among study results.	Not applicable – no heterogeneity investigation performed (p. 5)
	20d	Present results of all sensitivity analyses conducted to assess the robustness of the synthesized results.	Not applicable – no sensitivity analysis performed (p. 5)
Reporting biases	21	Present assessments of risk of bias due to missing results (arising from reporting biases) for each synthesis assessed.	Discussion – Limitations (publication bias, retrospective designs) (p. 12–13)
Certainty of evidence	22	Present assessments of certainty (or confidence) in the body of evidence for each outcome assessed.	Discussion – Limitations and conclusions sections (p. 12–13)
**DISCUSSION**			
Discussion	23a	Provide a general interpretation of the results in the context of other evidence.	Section 4 – Discussion, overall interpretation of findings (p. 11-12)
	23b	Discuss any limitations of the evidence included in the review.	Section 4 and 5 – Discussion, limitations of included evidence (p. 11-13)
	23c	Discuss any limitations of the review processes used.	Section 5 – Limitations of the review (p. 12-13)
	23d	Discuss implications of the results for practice, policy, and future research.	Section 4 – Discussion, implications for research and practice (p. 12)
**OTHER**			

Section and Topic	Item #	Checklist item	Location where item is reported
**INFORMATION**			
Registration and protocol	24a	Provide registration information for the review, including register name and registration number, or state that the review was not registered.	Section 2.1 – Not registered in PROSPERO (p. 4)
	24b	Indicate where the review protocol can be accessed, or state that a protocol was not prepared.	Section 2.1 – No protocol was prepared (p. 4)
	24c	Describe and explain any amendments to information provided at registration or in the protocol.	Section 2.1 - Not applicable (p. 3)
Support	25	Describe sources of financial or non-financial support for the review, and the role of the funders or sponsors in the review.	Funding section – Foundation for Science and Technology (FCT, Portugal) (p. 13)
Competing interests	26	Declare any competing interests of review authors.	Conflicts of Interest section – authors declare no conflicts (p. 13)
Availability of data, code and other materials	27	Report which of the following are publicly available and where they can be found: template data collection forms; data extracted from included studies; data used for all analyses; analytic code; any other materials used in the review.	Data Availability Statement – data from published studies, Supplementary Tables S1–S5 (p. 13)
